# Supplementary material for: Does an innovative paper-based health information system (PHISICC) improve data quality and use in primary healthcare? Protocol of a multicountry, cluster randomised controlled trial in sub-Saharan African rural settings
Source: BMJ Open. 2021 Jul 29;11(7):e051823. doi: 10.1136/bmjopen-2021-051823 (PMC8323359; doi:10.1136/bmjopen-2021-051823)
Supplement: Supplementary data [file bmjopen-2021-051823supp001.pdf]

CONSORT 2010 checklist of information to include when reporting a cluster randomised trial

Section/Topic	Item No	Standard Checklist item	Extension for cluster designs	Page No *
**Title and abstract**				
	1a	Identification as a randomised trial in the title	Identification as a cluster randomised trial in the title	1
	1b	Structured summary of trial design, methods, results, and conclusions	See table 2	5
**Introduction**				
**Background and objectives**	2a	Scientific background and explanation of rationale	Rationale for using a cluster design	4
	2b	Specific objectives or hypotheses	Whether objectives pertain to the cluster level, the individual participant level or both	5
**Methods**				
**Trial design**	3a	Description of trial design (such as parallel, factorial) including allocation ratio	Definition of cluster and description of how the design features apply to the clusters	6
	3b	Important changes to methods after trial commencement (such as eligibility criteria), with reasons		Not applicable
**Participants**	4a	Eligibility criteria for participants	Eligibility criteria for clusters	6
	4b	Settings and locations where the data were collected		6
**Interventions**	5	The interventions for each group with sufficient details to allow replication, including how and when they were actually administered	Whether interventions pertain to the cluster level, the individual participant level or both	8
**Outcomes**	6a	Completely defined pre-specified primary and secondary outcome measures, including how and when they were assessed	Whether outcome measures pertain to the cluster level, the individual participant level or both	10 and Table 1
	6b	Any changes to trial outcomes after the trial commenced, with reasons		Not applicable
**Sample size**	7a	How sample size was determined	Method of calculation, number of clusters(s) (and whether equal or unequal cluster sizes are assumed), cluster size, a coefficient of intracluster correlation (ICC or *k*), and an indication of its uncertainty	12
	7b	When applicable, explanation of any interim analyses and stopping guidelines		Not yet applicable

Section/Topic	Item No	Standard Checklist item	Extension for cluster designs	Page No *
**Randomisation:**				
**Sequence generation**	8a	Method used to generate the random allocation sequence		7, 8
	8b	Type of randomisation; details of any restriction (such as blocking and block size)	Details of stratification or matching if used	Not applicable
**Allocation concealment mechanism**	9	Mechanism used to implement the random allocation sequence (such as sequentially numbered containers), describing any steps taken to conceal the sequence until interventions were assigned	Specification that allocation was based on clusters rather than individuals and whether allocation concealment (if any) was at the cluster level, the individual participant level or both	7
**Implementation**	10	Who generated the random allocation sequence, who enrolled participants, and who assigned participants to interventions	Replace by 10a, 10b and 10c	7
	10a		Who generated the random allocation sequence, who enrolled clusters, and who assigned clusters to interventions	7
	10b		Mechanism by which individual participants were included in clusters for the purposes of the trial (such as complete enumeration, random sampling)	10
	10c		From whom consent was sought (representatives of the cluster, or individual cluster members, or both), and whether consent was sought before or after randomisation	8
**Blinding**	11a	If done, who was blinded after assignment to interventions (for example, participants, care providers, those assessing outcomes) and how		8
	11b	If relevant, description of the similarity of interventions		Not applicable
**Statistical methods**	12a	Statistical methods used to compare groups for primary and secondary outcomes	How clustering was taken into account	15
	12b	Methods for additional analyses, such as subgroup analyses and adjusted analyses		15

Section/Topic	Item No	Standard Checklist item	Extension for cluster designs	Page No *
**Results**				Not yet applicable (protocol manuscript)
**Discussion**				
**Limitations**	20	Trial limitations, addressing sources of potential bias, imprecision, and, if relevant, multiplicity of analyses		18
**Generalisability**	21	Generalisability (external validity, applicability) of the trial findings	Generalisability to clusters and/or individual participants (as relevant)	18,19
**Interpretation**	22	Interpretation consistent with results, balancing benefits and harms, and considering other relevant evidence		Not yet applicable
**Other information**				
**Registration**	23	Registration number and name of trial registry		1
**Protocol**	24	Where the full trial protocol can be accessed, if available		1
**Funding**	25	Sources of funding and other support (such as supply of drugs), role of funders		20
