# Supplementary material for: Does an innovative paper-based health information system (PHISICC) improve data quality and use in primary healthcare? Protocol of a multicountry, cluster randomised controlled trial in sub-Saharan African rural settings
Source: BMJ Open. 2021 Jul 29;11(7):e051823. doi: 10.1136/bmjopen-2021-051823 (PMC8323359; doi:10.1136/bmjopen-2021-051823)
Supplement: Supplementary data [file bmjopen-2021-051823supp002.pdf]

```
#
# clustersampleSize_proportions_baseline&endline.r
# get power of cluster randomised trial for binary outcomes (baseline and
endline surveys)
# 2 groups (control & intervention)
# clustered within HF


rm(list=ls())

# if the package lme4 is not already installed (needed for regression with
random effects)
# install.packages(lme4)
require(lme4)
#install.packages("reshape")
library(reshape)


# INPUTS
numGroups<-2
numHFPerGroup<-35
numTrialsToSimulate<-100
# numTrialsToSimulate: use 10 to test that the script runs, use 100 or 1000 for
precise estimate of power



# choose input set and remove #s to run

# inputs for 'treatments with appropriate diagnosis'
 pInterv<-0.60
 pControl<-0.50
 sdHFcluster<-0.55
# for k=0.1, 0.20; for k=0.25, 0.55
 numObsPerHF<-25

# inputs for vaccination adherence
# proportions in interventions and control groups
# pInterv<-0.8
# pControl<-0.75
# sdHFcluster<-2.63
# numObsPerHF<-30


# inputs for 'more than one diagnosis'
# pInterv<-0.35
# pControl<-0.30
# sdHFcluster<-0.39
# for k=0.1, 0.16; for k=0.25, 0.39
# numObsPerHF<-60


# NB getsd is a function at the bottom of the script to turn k into sdHFcluster
(sdHFcluster is on the logit scale)
```

```
# --- simulation ----

  # SET UP DATA STRUCTURE (intervention, HF)
  totNumHF <- numHFPerGroup*numGroups
  HFList<-seq(1:totNumHF)
  interv<- rep(c(0,1),each=(totNumHF/2) )
  intervEffect<-rep( c(0,(log(pInterv/(1-pInterv)) -
log(pControl/(1-pControl))) ), each=(totNumHF/2) )

  xtemp<-cbind(interv,HFList,intervEffect)

  # SET UP STORE FOR PVALUES AND PRECISION
  storeResults<-array(-9,dim=c(numTrialsToSimulate,3))
  colnames(storeResults)<-c("pvalue","coeff","stderr")


  # LOOP THROUGH THE SIMULATIONS

  for (i in 1:numTrialsToSimulate) {

    # simulate the HF cluster effects
      HFEffect<-rnorm(totNumHF,mean=0,sd=sdHFcluster)
      xtemp2a<-cbind(xtemp, HFEffect)
      xtemp2a<-data.frame(xtemp2a)

      # get expected proportions (pre and post)
      xtemp2a$expectedprelogodds<-log(pControl/(1-pControl)) + xtemp2a$HFEffect

      xtemp2a$expectedpostlogodds<-log(pControl/(1-pControl)) +
xtemp2a$intervEffect + xtemp2a$HFEffect

xtemp2a$expectedpre<-exp(xtemp2a$expectedprelogodds)/(1+exp(xtemp2a$expectedpre
logodds))

xtemp2a$expectedpost<-exp(xtemp2a$expectedpostlogodds)/(1+exp(xtemp2a$expectedp
ostlogodds))

      # expand by the number of observations per HF
      xtemp2b<-untable(xtemp2a, num=numObsPerHF)
      numObs<-dim(xtemp2b)[1]

    # simulate individual observations from cluster mean rates
      simObsPost<-rep(0,numObs)
      simObsPre<-rep(0,numObs)
      for (j in 1:numObs) {
         simObsPost[j]<-rbinom(n=1, size=1,prob=xtemp2b$expectedpost[j])
         simObsPre[j]<-rbinom(n=1, size=1,prob=xtemp2b$expectedpre[j])
      }
      # drop variables not needed further
      xtemp2b$expectedpostlogodds<-NULL; xtemp2b$expectedprelogodds<-NULL
```

```
    # stack pre and post observations
      # get post
      xtemp3<-cbind(xtemp2b,simObsPost)
      xtemp3<-data.frame(xtemp3)
      xtemp3$simObs<-xtemp3$simObsPost
      xtemp3$simObsPost<-NULL
      xtemp3$post<-1
      # get pre
      xtemp4<-cbind(xtemp2b,simObsPre)
      xtemp4<-data.frame(xtemp4)
      xtemp4$simObs<-xtemp4$simObsPre
      xtemp4$simObsPre<-NULL
      xtemp4$post<-0
      xtemp4$interv<-0
      xtemp5<-rbind(xtemp3,xtemp4)


     # carry out analysis for individual trial
      m <- glmer(simObs ~ as.factor(interv) +  post + (1 | HFList), 
data<-xtemp5, family=binomial)

     # store result of individual trial in storeResults (p-value, coefficient 
and std error)
          out1<-summary(m)$coefficients
          storeResults[i,2]<-out1[2,1]
          storeResults[i,3]<-out1[2,2]
          storeResults[i,1]<-out1[2,4]

   print(i)

  }  # End of loop

  # calculate power
  pvalue<-storeResults[,1]
  power<-length(pvalue[pvalue<0.05])/length(pvalue)

  cat("power ", power, "\n")



# -------- run to here -----







# ------
# getsd: function to estimate between-cluster variation from k (Hayes and 
Bennet sd/mean) and input base proportion (base0p)
```

```
getsd<-function(base0p,k){
     sdcluster<-k*base0p
     clusterEffect<-rnorm(1000,mean=0,sd=sdcluster)
     expectedp<-base0p + clusterEffect
     expectedp[expectedp>1]<-0.9999
     expectedp[expectedp<0]<-0.0001
     logitexpectedp<-log((expectedp)/(1-expectedp))
     sdlog<-sd(logitexpectedp)
     cat("estimated sdlog ", sdlog, "\n")
}

# example
getsd(0.30,0.25)

getsd(0.50, 0.25)
```
