# Supplementary material for: Does an innovative paper-based health information system (PHISICC) improve data quality and use in primary healthcare? Protocol of a multicountry, cluster randomised controlled trial in sub-Saharan African rural settings
Source: BMJ Open. 2021 Jul 29;11(7):e051823. doi: 10.1136/bmjopen-2021-051823 (PMC8323359; doi:10.1136/bmjopen-2021-051823)
Supplement: Supplementary data [file bmjopen-2021-051823supp003.pdf]

```
#
# clusterSampleSize_concordance.r
# get power of cluster randomised trial
# ratios (outcome is continuous)
# fixed to 2 groups
# records and reports clustered within HF
#


# if the package lme4 is not already installed (needed for regression with
random effects)
# install.packages(lme4)
# install.packages(lmerTest)
require(lme4)
require(lmerTest)


# EXAMPLE INPUTS
numGroups<-2
numHFPerGroup<-35
numReportedPerHF<-100
# assuming equal numbers of vaccinations per HF
numTrialsToSimulate<-100
# 100 or 1000 needed for precision of the power estimate, use 10 for test runs

ratioControl<-0.7
ratioInterv<-0.8
sdHFcluster<-0.25*0.8
# sdHFcluster is on the log scale, calculated using k=0.25



# --- run simulation from here ----

  # SET UP DATA STRUCTURE (intervention, HF)
  totNumHF<-numGroups*numHFPerGroup
  HFList<-rep(seq(1:(numHFPerGroup*numGroups)),each=1)
  interv<-c( rep(c(0,1),each=(totNumHF/2)))
  intervEffect<-rep( c(0,(ratioInterv - ratioControl )), each=(totNumHF/2) )
  xtemp<-cbind(interv,HFList,intervEffect)

  # SET UP STORE FOR PVALUES AND PRECISION
  storeResults<-array(-9,dim=c(numTrialsToSimulate,3))
  colnames(storeResults)<-c("pvalue","coeff","stderr")


  # LOOP THROUGH THE SIMULATIONS

  for (i in 1:numTrialsToSimulate) {

    # simulate the HF cluster effects

      HFEffect<-rnorm(numHFPerGroup*numGroups,mean=0,sd=sdHFcluster)
      xtemp2<-cbind(xtemp, HFEffect)
```

```
     # get expected ratios (pre and post)
      expectedpreratio<-ratioControl + HFEffect
      expectedpostratio<-ratioControl + intervEffect + HFEffect
      expectedpreratio[expectedpreratio<0.0001]<-0.0001
      expectedpostratio[expectedpostratio<0.0001]<-0.0001

     # simulate individual observations as poisson rate of number reported per
1 recorded
      simObsPost<-rep(0,length(expectedpostratio))
      simObsPre<-rep(0,length(expectedpreratio))
      for (j in 1:length(expectedpostratio)) {
          simObsPost[j]<-rpois(n=1,expectedpostratio[j]*numReportedPerHF)
          simObsPre[j]<-rpois(n=1,expectedpreratio[j]*numReportedPerHF)
      }


    # stack pre and post observations
      # post
      xtemp3<-cbind(xtemp2,simObsPost)
      xtemp3<-data.frame(xtemp3)
      xtemp3$simObs<-xtemp3$simObsPost
      xtemp3$simObsPost<-NULL
      xtemp3$post<-1
      # pre
      xtemp4<-cbind(xtemp2,simObsPre)
      xtemp4<-data.frame(xtemp4)
      xtemp4$simObs<-xtemp4$simObsPre
      xtemp4$simObsPre<-NULL
      xtemp4$post<-0
      xtemp4$interv<-0
      # stack pre and post
      xtemp5<-rbind(xtemp3,xtemp4)
        xtemp5$distanceToOne<-abs(1-(xtemp5$simObs/numReportedPerHF))


      # carry out analysis for individual trial
      m <- lmer(distanceToOne ~ as.factor(interv) +  post + (1|HFList),
data=xtemp5)

      # store result of individual trial in storeResults (p-value, coefficient
and std error)
       out1<-summary(m)$coefficients
       # estimate
       storeResults[i,2]<-out1[2,1]
       # se
       storeResults[i,3]<-out1[2,2]
       # p-value
       storeResults[i,1]<-out1[2,5]

     print(i)

  }  # End of loop
```

```
  # calculate power
  pvalue<-storeResults[,1]
  power<-length(pvalue[pvalue<0.05])/length(pvalue)

  cat("power ", power, "\n")


# -------------
```
