# Supplementary material for: Does an innovative paper-based health information system (PHISICC) improve data quality and use in primary healthcare? Protocol of a multicountry, cluster randomised controlled trial in sub-Saharan African rural settings
Source: BMJ Open. 2021 Jul 29;11(7):e051823. doi: 10.1136/bmjopen-2021-051823 (PMC8323359; doi:10.1136/bmjopen-2021-051823)
Supplement: Author's manuscript [file bmjopen-2021-051823.draft_revisions.pdf]

BMJ Open is committed to open peer review. As part of this commitment we make the peer review history of every article we publish publicly available.

When an article is published we post the peer reviewers' comments and the authors' responses online. We also post the versions of the paper that were used during peer review. These are the versions that the peer review comments apply to.

The versions of the paper that follow are the versions that were submitted during the peer review process. They are not the versions of record or the final published versions. They should not be cited or distributed as the published version of this manuscript.

BMJ Open is an open access journal and the full, final, typeset and author-corrected version of record of the manuscript is available on our site with no access controls, subscription charges or pay-per-view fees (http://bmjopen.bmj.com).

If you have any questions on BMJ Open's open peer review process please email info.bmjopen@bmj.com

BMJ Open

Does an innovative paper-based health information system (PHISICC) improve data quality and use in primary health care? Protocol of a multi-country, cluster randomised controlled trial in Sub-Saharan African rural settings.

Journal:	*BMJ Open*
Manuscript ID	bmjopen-2021-051823
Article Type:	Original research
Date Submitted by the Author:	02-Apr-2021
Complete List of Authors:	Bosch-Capblanch, Xavier; Swiss Tropical and Public Health Institute<br>Oyo-Ita, Angela; University of Calabar, Department of Community Medicine<br>Muloliwa, Artur; Lurio University Faculty of Health Sciences<br>Yapi, Richard; Centre Suisse de Recherches Scientifiques en Côte d'Ivoire<br>Auer, Christian ; Swiss Tropical and Public Health Institute<br>Samba, Mamadou; Ministère de la Santé et de l'Hygiène Publique<br>Gajewski, Suzanne; Swiss Tropical and Public Health Institute<br>Ross, Amanda; Swiss Tropical and Public Health Institute<br>Krause, L Kendall; Bill & Melinda Gates Foundation<br>Ekpenyong, Nnette; University of Calabar Teaching Hospital, Department of Community Medicine<br>Nwankwo, Ogonna; University of Calabar, Department of Community Medicine<br>Njepuome, Anthonia; Swiss Tropical and Public Health Institute<br>Lee, Sofia; Swiss Tropical and Public Health Institute<br>Sacarlal, Jahit ; Eduardo Mondlane University, Faculty of Medicine<br>Madede, Tavares; Universidade Eduardo Mondlane<br>Berté, Salimata; Centre Suisse de Recherches Scientifiques en Côte d'Ivoire<br>Matsinhe, Graça; Ministério da Saúde<br>Garba, Abdullahi; National Primary Healthcare Development Agency<br>Brown, David; BCGI LLC / pivot-23.5°
Keywords:	Human resource management < HEALTH SERVICES ADMINISTRATION & MANAGEMENT, Organisation of health services < HEALTH SERVICES ADMINISTRATION & MANAGEMENT, Protocols & guidelines < HEALTH SERVICES ADMINISTRATION & MANAGEMENT, Quality in health care < HEALTH SERVICES ADMINISTRATION & MANAGEMENT, STATISTICS & RESEARCH METHODS

SCHOLARONE™
Manuscripts

I, the Submitting Author has the right to grant and does grant on behalf of all authors of the Work (as defined in the below author licence), an exclusive licence and/or a non-exclusive licence for contributions from authors who are: i) UK Crown employees; ii) where BMJ has agreed a CC-BY licence shall apply, and/or iii) in accordance with the terms applicable for US Federal Government officers or employees acting as part of their official duties; on a worldwide, perpetual, irrevocable, royalty-free basis to BMJ Publishing Group Ltd ("BMJ") its licensees and where the relevant Journal is co-owned by BMJ to the co-owners of the Journal, to publish the Work in this journal and any other BMJ products and to exploit all rights, as set out in our licence.

The Submitting Author accepts and understands that any supply made under these terms is made by BMJ to the Submitting Author unless you are acting as an employee on behalf of your employer or a postgraduate student of an affiliated institution which is paying any applicable article publishing charge ("APC") for Open Access articles. Where the Submitting Author wishes to make the Work available on an Open Access basis (and intends to pay the relevant APC), the terms of reuse of such Open Access shall be governed by a Creative Commons licence – details of these licences and which Creative Commons licence will apply to this Work are set out in our licence referred to above.

Other than as permitted in any relevant BMJ Author's Self Archiving Policies, I confirm this Work has not been accepted for publication elsewhere, is not being considered for publication elsewhere and does not duplicate material already published. I confirm all authors consent to publication of this Work and authorise the granting of this licence.

For peer review only

Title

Does an innovative paper-based health information system (PHISICC) improve data quality and use in primary health care? Protocol of a multi-country, cluster randomised controlled trial in Sub-Saharan African rural settings.

Authors

- Xavier Bosch-Capblanch: Corresponding author. Swiss Tropical and Public Health Institute. Basel, Switzerland. And University of Basel, Basel, Switzerland. x.bosch@unibas.ch
- Angela Oyo-Ita Department of Community Medicine, University of Calabar, Calabar, Nigeria; oyo_ita@yahoo.com
- Artur Manuel Muloliwa. Faculty of Health Sciences, Lúrio University, Nampula, Mozambique; muloliwa@yahoo.com.br
- Richard B Yapi. Centre Suisse de Recherches Scientifiques en Côte d'Ivoire, Abidjan, Côte d'Ivoire. And Centre d'Entomologie Médicale et Vétérinaire, Université Alassane Ouattara, Bouaké, Côte d'Ivoire. richard.yapi@csrs.ci
- Christian Auer. Swiss Tropical and Public Health Institute. Basel, Switzerland. And University of Basel, Basel, Switzerland. christian.auer@swisstph.ch
- Mamadou Samba. Ministère de la Santé et de l'Hygiène Publique, Abidjan, Côte d'Ivoire. And Université Félix Houphouet Boigny, Abidjan, Côte d'Ivoire. samba.mamadou@gmail.com
- Suzanne Gajewski. Swiss Tropical and Public Health Institute. Basel, Switzerland. And University of Basel, Basel, Switzerland. suzanne.gajewski@swisstph.ch.
- Amanda Ross. Swiss Tropical and Public Health Institute. Basel, Switzerland. And University of Basel, Basel, Switzerland. amanda.ross@swisstph.ch
- L Kendall Krause. Bill & Melinda Gates Foundation, Seattle, USA. Kendall.Krause@gatesfoundation.org
- Nnette Ekpenyong. Department of Community Medicine, University of Calabar Teaching Hospital, Calabar, Nigeria. And Department of Community Medicine University of Calabar, Calabar, Nigeria. nnekon2015@gmail.com
- Ogonna Nwankwo. Swiss Tropical and Public Health Institute, Basel, Switzerland. And University of Basel, Basel, Switzerland. And Health Policy, Management and Economics Firm, Department of Community. Medicine, University of Calabar, Calabar, Nigeria. ogonna.nwankwo@swisstph.ch
- Anthonia Ngozi Njepuome. Swiss Tropical and Public Health Institute, Abuja, Nigeria. ngonjep@yahoo.com.
- Sofia Mandjate Lee. Swiss Tropical and Public Health Institute; Manhiça, Mozambique. sofia_mandjate@yahoo.com.br.
- Jahit Sacarlal. Faculty of Medicine, Eduardo Mondlane University, Maputo, Mozambique. jahityash2002@gmail.com.
- Tavares Madede. Department of Community Health, Faculty of Medicine, Eduardo Mondlane University, Maputo, Mozambique. tmadede@gmail.com
- Salimata Berté. Centre Suisse de Recherches Scientifiques en Côte d'Ivoire, Abidjan, Côte d'Ivoire. sali.berte@csrs.ci
- Graça Matsinhe. Expanded Program on Immunization, Ministry of Health, Maputo, Mozambique. gmatsinhe@gmail.com.
- Abdullahi Bulama Garba. Director Planning, Research and Statistics, National Primary Health Care. Development Agency, Abuja, Nigeria. ab.garba@nphcda.gov.ng.
- David W Brown. Managing Principal, BCGI LLC / pivot-23.5°, North Carolina, USA. david.brown@pivot235.org

For peer review only

Abstract

Introduction

Frontline health workers in remote, rural health facilities are the first contact of the formal health sector and are confronted with life-saving clinical and public health decisions. Appropriate health information systems (HIS) support the collection and use of data, thus facilitating decision-making. However, HIS focus on reporting and are unfit to support critical decisions at the peripheral level. Since data tools are paper-based in most primary health care settings, we have produced an innovative paper-based HIS (PHISICC), embracing all health care areas in primary health care, using a Human Centred Design, co-creation approach. The PHISICC tools aid decision-making and include recording and reporting. We are carrying out a cluster-randomised controlled trial in three African countries to assess the effects of PHISICC compared with the current systems, on data use and quality, quality of care and health worker perceptions, in remote, rural settings.

Methods

We have selected study areas in rural zones of Côte d'Ivoire, Mozambique and Nigeria. Seventy health facilities in each country have been randomly allocated to using PHISICC tools or to continuing to use the regular HIS tools (35 per arm). We have selected three villages in the catchment area of each health facility to carry out surveys in 10 households each. Outcomes of interest include data quality and use, coverage of health services, health workers perceptions and other process and explanatory variables.

Discussion

We strive to contribute to producing robust evidence on health systems interventions, affecting people in remote, rural settings where the most vulnerable live. The PHISICC tools focus on decision-making rather than data and are meant to support health workers decisions as well as reporting to the higher levels of the system. Robust evidence on HIS can better find its way to high quality systematic reviews and guidance development to inform policy and practice.

Trial registration: Pan African Clinical Trials Registry - PACTR201904664660639. Registered 01/04/2019, https://pactr.samrc.ac.za/Search.aspx.

Keywords

Decision-making | Health Information Systems | Primary Health Care | Sub-Saharan Africa | Data quality | Quality of care | Human Centred Design

For peer review only

Article summary

Strengths and limitations of the study

- This research assesses the effects of paper-based health information systems, which are massively used particularly in remote, rural areas but which seriously neglected in research.
- The paper-based interventions have been developed using a human-centred design approach, with frontline health workers and designers driving the co-creation process.
- Despite the complexity of health systems interventions like this one, we have applied robust experimental methods, together with qualitative research, to assess and understand the effects of the paper-based intervention. Robust evidence on health systems is more likely to gain the credibility of policy-makers and to make it into systematic reviews, guidance development and policy and practice.
- Research targeting frontline health workers in remote, rural areas has to take place where they live and work, which poses serious obstacles in the organisation, management and monitoring of the trials.
- These obstacles, aggravated by the COVID-19 pandemic, have challenged the mobility of the research team, the availability of the intervention in one of the countries and the duration of the trials.

For peer review only

Introduction

Frontline health workers (HW) in remote, rural health facilities (HF) in many countries are the first contact with the formal health sector of the population and they are confronted with life-saving clinical and public health decisions on a daily basis. Decisions are made by exerting a balanced judgment on the information related to health care events, such as making the correct diagnoses or deciding on which vaccinations a child should receive on a given day. In order to properly handle this information, appropriate data support tools and processes are required, referred to as the health information system (HIS); or Routine HIS or Health Management Information System [1]. In reality, though, HIS are primarily designed to report aggregated health events to the higher tiers of the health systems rather than to inform decision-making at the point of care [2].

Increasing pressure by donors and governments to collect more and more data has aggravated the situation, through the proliferation of data support tools that have overloaded frontline health workers compromising their capacity to deliver good quality of care and to delivery good quality data [3], for higher level decision-making.

Promising 'quick fixes', such as the scale up of digital HIS, are taking a long time to implement and face enormous challenges related to infrastructure, equipment and services necessary to run them. Besides, research evidence on the effects of digital solutions remains patchy and inconsistent, even in high-income country settings, where complaints about computerisation of clinical care have been raised [4,5]. Hence, it is very likely that paper tools will remain a primary, if not unique, data support mechanism particularly in remote, rural HF in many countries.

PHISICC (Paper-based Health Information System in Comprehensive Care) is a multi-year, multi-country, mixed-methods research project that aims at producing and testing an innovative paper-based HIS to improve data quality and use, decision making and health outcomes, at Primary Health Care (PHC). It is being carried out in selected areas within Côte d'Ivoire, Mozambique and Nigeria. The project started in 2015, producing a systematic review on the effects of HIS interventions and a

For peer review only - http://bmjopen.bmj.com/site/about/guidelines.xhtml

framework synthesis on how HIS are understood in the literature. These were followed by studies to characterise the existing HIS in the three countries. With these bodies of evidence, we engaged into a Human Centred Design (HCD) co-creative process with frontline HW to design an innovative HIS (PHISICC).

The impact of the PHISICC HIS on data quality and use, quality of health care and HW perceptions is being assessed concurrently in rural areas in the three countries. We describe the design of the trial here, consistent with CONSORT reporting guidelines [6] and the extension for cluster randomised controlled trials (CRCT) [7]; see Additional file 1.

Methods

Aim

The aim of the trial is to address the research question: what are the effects of an innovative paper-based HIS (PHISICC) on data use and quality, quality of health and HW perceptions compared with the current HIS, in rural PHC settings?

Patient and public involvement

There was no public or patient involvement in this research because the intervention being assessed in these trials target health care providers and decision-makers, rather than patients or the public in general. We have involved health systems stakeholders and frontline health workers. Ministries of Health at several levels participated in the preparation of the research proposal (personal consultations), in the characterisation of health information systems that preceded the trials (countries workshops), and throughout all project components (additional workshops, newsletters and personal communication). Frontline health workers in the three countries have co-created the intervention (i.e. paper based tools) through workshops, personal feedback and piloting under real live conditions. Some of them are part of the research team and co-authoring this manuscript.

Study design

The study is a CRCT in each of the three countries. In each setting, 70 health facilities are randomised to intervention or control (35 per arm). The intervention arm HF use the new PHISICC tools (substituting the usual HIS tools) and the control arm HF use the regular HIS tools. The trial is implemented in the real life contexts of HF carrying out their usual duties.

The CRCT are implemented in the real life contexts of HF carrying out their usual duties. The trials started between the end of 2019 and beginning of 2020, depending on the country, when the intervention was installed and the baseline surveys carried out; and will last till mid-2021.

Study areas

Ministries of Health (MOH) officials in several countries were contacted before submitting the proposal to the funding agency in order to explore the willingness to engage in a project focusing on paper-based tools. Officials in several countries rejected the offer on the grounds of upcoming digitalisation plans of the HIS in the country. We partnered with MOH that found the research relevant to their context in three countries.

In each country, the eligibility criteria of study areas were that they had to belong to the operational area of research partners; contain a large enough number of health facilities and their catchment population; include vulnerable population (e.g. with low vaccination coverage, high childhood mortality); and be comparatively neglected in terms of infrastructure and services. We excluded areas with concurrent research or other types of activities that could conflict with the CRCT (such as the co-existence of another health-related study, massive developments in infrastructure or activities involving migration of the population, such as temporary work sites or changes in working sites) and areas with threats to safety or security that could jeopardise research activities.

The study areas are located in Adzopé, Agboville, Tiassalé and Sikensi districts (Côte d'Ivoire); in Funhalouro, Govuro, Homoine, Inhambane, Inharrime, Inhassoro, Mabote, Maxixe and Panda

For peer review only - http://bmjopen.bmj.com/site/about/guidelines.xhtml

(Inhambane province, Mozambique); and in Yala Local Government Authority (Cross-River State, Nigeria).

Eligibility of health facilities

The intervention is implemented at the HF level. The eligibility criteria of the HF were that they had to be located in the study areas, belong to the governmental health sector and their main activity should be the delivery of PHC services. HF were excluded if they had specialised clinical services, inpatients, physicians providing care or with plans for staff turn-over involving intervention and control HF.

A 'master list' of eligible health facilities was prepared based on information provided by the MOH across all study areas. We aimed at selecting 70 of the eligible HF in each country, using simple random techniques in R [8].

Allocation and blinding

Allocation of the 70 HF per country into the intervention and control arms took place in a formal event, gathering research partners and MOH officials to offer transparency and promote study ownership by local and national authorities. Equally sized, folded pieces of paper with the names and codes of included HF written on them were introduced in an opaque receptacle where they were manually and blindly mixed. A second receptacle contained two equally sized pieces of paper, one with the word 'intervention' and another one with the word 'control'. A selected person in the meeting, not belonging to the research team, extracted one piece of paper at a time to reach half the number of included HF. Then, a paper was extracted from the second receptacle to assign those HF to the intervention or control arms. The rest of the papers were extracted as well to verify completeness and no duplication of names, and those HF assigned to the other arm.

Once HF were selected, all villages or settlements for each health facility catchment area were listed and three in each catchment area were selected. In practice, we selected all villages because the numbers were below (in Côte d'Ivoire) or just above (in Nigeria) the needs. For each village, we used

For peer review only

Google satellite maps to identify and geo-locate every visible roof. Where there were many houses per village (roughly, more than fifty or so), a researcher would mark four points in the map slightly beyond the northernmost, southernmost, easternmost and westernmost roofs seen and 30 random points were selected within that square. From the mapped points, 10 per village (with 10 more acting as reserve) were randomly selected and marked on another map used in the field for data collectors to approach households. Where technical problems impeded this approach in a given village, a field supervisor would rotate a bottle on the floor towards the centre of the village and would select at random 10 households in the direction pointed by the bottle, from the outer limit of the village till the centre [9].

Blinding is only feasible for the research team members carrying out the CRCT data collection and the analyses of the CRCT findings. The intervention (i.e. paper tools) are by design very different from the existing system and it is not possible to blind participants or principal researchers.

We already had the agreement of the MOH and selected HF compliant with the inclusion criteria were provided with the intervention shortly after completing the baseline data collection. Therefore, recruitment as such took place at the same time of the allocation of HF into intervention and control arms.

The intervention

The PHISICC paper-based intervention is a full set of paper-based tools to support decision-making by frontline HW. These are the only tools to be used by HW in the intervention arm. The PHISICC tools encompass the whole system (i.e. recording and reporting) and all clinical and public health care areas and are characterised by: a common visual language (e.g. spaces for digits and text), standardised formats across health care areas; support to critical data items (e.g. respiratory rate in infants); graphic artefacts to distinguish severity degrees of signs or symptoms; documentation of diagnoses and treatment decisions; and aides memoires, among others.

For peer review only

The PHISICC tools have been developed over 18 to 20 months prior to the CRCT, using a Human Centred Design approach [10]. A strength of the Human Centred Design approach is its ability to unlock the user's perspective so that designers can build solutions that are fully reality-based and work well. Co-creation groups were formed in each country with researchers, staff from partner institutions and healthcare workers, led by a team of professional designers. Based on co-creation, participatory processes, and Human Centred Design principles, many iterations took place between co-creation groups and end-users of the tools, the frontline HW, till reaching a design that considered and addressed the main issues raised by HW (i.e. usability, clarity, size of tools). The PHISICC tools have been produced in French for Côte d'Ivoire, in Portuguese for Mozambique and English for Nigeria, which are the official languages used in the health systems in the three countries; using the official logo of the MOHs. Health care areas covered include: family planning, antenatal care, including tetanus toxoid vaccination, delivery, post-natal care, vaccination, sick child, adults outpatient consultation, tuberculosis diagnosis and treatment, and HIV. Referral forms were also designed.

The PHISICC tools have three sub-components: registers, tallies and reports. Registers are formed by seven DIN-A3 and one DIN-A4 (for referrals) book covering all health care areas except for tuberculosis treatment, for which DIN-A3 cards where used. Register books have 100, 200 or 400 pages depending on the country and health care area. They are used to record individual clients' data for each health care event, either of clinical or public health nature. Some register books have clinical notes at the very beginning, as 'aide memoires', and an example of a filled-in form, to assist HW when doubting how to proceed.

Tallies are DIN-A3 single sheets which contain a list of the indicators to be transferred to higher levels of the health system, with a series of small ovals, grouped in fives, to mark with tally sticks with a pen. In contrast to the current systems that have no tallies or only for vaccination, tallies were created for all health care areas. In the middle-right side of the tally, a column accommodates cells aligned with the ovals to insert the count for each indicator; and in the far right of the sheet there is

For peer review only

a replica of the count column, separated with a perforated line, which is detached and sent, as part of the monthly report to the higher level in the health system.

During three or four days, HW were trained on HIS before the start of the trial. In the intervention arm they were trained on the PHISICC tools; and the control arm received a refresher training about the regular tools, during the same number of days.

Additionally, given that the regular tools already contained information on past vaccination history of children still to complete their vaccination schedule, we created a mechanism to retrieve data of children's vaccination status to transcribe into the new vaccination register book in the intervention arm ('system transition').

Tools were endorsed by MOH, printed in local printing companies and distributed to HW at the end of the training sessions. A digital spreadsheet was created to monitor consumption and order additional tools to cover health facility needs during the life of the trial.

Outcomes

There are five primary outcomes (Table 1). Vaccination adherence is defined as the total number of vaccine doses given during the trial period in the correct time interval to children over the total number of vaccine doses that should have been given during the same period. Antenatal care visits uptake will also be considered depending on the expected number of pregnancies in the study areas. Both are used as proxies for health outcomes in terms of protection against disease [11] and prevention of pregnancy complications [12]. Data concordance is defined as the level of agreement of HIS indicators between (i) records, (ii) tallies and (iii) reports [3]. In terms of data use for decision making, we will estimate the diagnostics scope in the sick child (i.e. number of different diagnoses per child; and treatment appropriateness (i.e. number of prescribed treatments that are supported by a documented diagnosis). Health workers satisfaction will be assessed using a standardised questionnaire [13,14,15]. While the intervention targets HF, some of the outcomes are measured at the level of HF, and some from patients clustered within HF catchment areas.

For peer review only

Secondary outcomes are classified under the following domains: data quality, data user, mortality, HW experience, clients experience and resource consumption:

- Data quality
    - Completeness of recording and reporting in specific forms; i.e. prevalence of unduly missing data items; partograph used;
    - accuracy of recorded figures in comparison to real events (e.g. physical counting of commodities, such as number of 500mg Paracetamol tablets as recorded versus number of 500mg Paracetamol tablets as counted;
    - timeliness of reporting, as documented by time stamps in forms;
    - loss of data or data which does not reach the next upper administrative level.
- Data use
    - in terms of knowledge (e.g. vaccines due based on date of birth; weight for length assessments);
    - cases of different conditions properly treated in (e.g. diarrhoea cases given oral rehydration therapy according to national guidelines; pneumonia cases given appropriate antibiotic according to national guidelines;
    - public health decisions: availability of lost to follow up lists or plans for vaccination, tuberculosis and or HIV/AIDS treatment control;
    - occurrence of stock outs of essential drugs
- Overall under-5s mortality and under-5s mortality excluding peri-natal mortality [16].
- Health workers 'human experience' and satisfaction
- District health information officers' 'human experience'
- Clients 'human experience' and satisfaction

For peer review only

- Resources consumption (e.g. time use, costs)
    - intervention costs: tools, training, start-up;
    - time used for recording and reporting (e.g. time-motion study) [17];
    - cost-effectiveness per unit of additional improvement in outcomes of interest.

In addition, we will consider 'explanatory outcomes' that will help to understand how the measured effects have taken place and why. We will look at the details of the interplay between the intervention, the system, the users and the context. Process indicators will be based on the documented activities that have taken place, from the conception of the intervention, up to its implementation, monitoring and evaluation. Process indicators may include: intervention set up and implementation, monitoring of the use of the intervention, special activities targeted at vulnerable populations, district reactions related to the intervention, handling of data coming from the new system, sustainability based on costs information and perceptions, alignment with national health policies and donor priorities. We will also explore health care services characteristics looking at generic indicators from health facilities, such human resources profiles and relations with the communities, population characteristics and system and context characteristics captured in early stages of the project, where data are available.

For peer review only

Sample size calculations

The required sample sizes for each primary outcome were determined using simulation. This allowed us to account for levels of clustering (Table 1). We used the regression models detailed in the data section to analyse the simulated trials and estimate the power. The simulation code was written in R.

For each country, we required the probability of a type I error (rejecting the null hypothesis when it is actually true) [illegible] to be less than 0.05 and a power of 80%.

The sampling frames are the study areas in each country, which include the health facilities and households in their catchment areas.

For vaccination adherence, using a sample size of 35 HF per arm, we would have 80% power in each country to detect as significant a difference between a proportion of due vaccines given from 75% in the control to 85% in the intervention arms, assuming one child per household, 30 households per HF and a between-HF variation equivalent to a k of 0.1, where k is equal to the standard deviation divided by the mean. The value of k is unknown, but was chosen in line with general observations by Hayes and Bennet [18].

For data quality outcomes, with 35 HF per arm we would be able to detect as significant a difference from a ratio of 0.7 (reported : recorded) vaccinations in the control arm to 0.8 with the intervention with 80% power, assuming 100 recorded vaccinations per HF and a standard deviation of 0.1 in the ratios between HF.

In terms of diagnostic scope, we would be able to detect an increase in the proportion of child-visits with more than one diagnosis from 30% to 35% with 80% power with 35 HF per arm, 60 records per HF and assuming a k of 0.1 [**Error! Bookmark not defined.**].

We would be able to detect as significant an increase from 50% of treatments having a corresponding appropriate diagnosis to 60% with 80% power assuming 35 HF per arm, 1 treatment per child, 25 children per HF and variation between HF corresponding to k = 0.1 [**Error! Bookmark not defined.**].

For the outcome related to health workers' satisfaction, we would be able to detect as significant an increase from 50% of health workers satisfied to 90%, with 80% power assuming 35 HF, three health workers per HF and a variation between HF equivalent to k = 0.1.

In summary, in each country we require 35 HF per arm, three HW per HF, 100 vaccination records per HF, 60 sick child records per health facility and 30 children per health facility catchment area.

For peer review only

Data collection and management

Data collection took place at baseline and will take place again at the end of the study. Data is collected from health facilities, from the households in the catchment areas of the included health facilities and also from district offices.

For data quality and data use outcomes, HF registers, tallies and reports will be scrutinised. For population based outcomes, we carry out household surveys at baseline and at end-line. We use standard approaches for these types of surveys [19]. Households are visited, the research project is briefly introduced and consent requested.. Ideally, mothers of alive children or women in child-bearing age were interviewed in order to obtain information on living children (i.e. vaccination history) and death events, respectively, using home-based records if available and accessible. Patients' satisfaction will be assessed using the PSQ-18 satisfaction questionnaire [20,21,22]. Essentially, the tool enables practitioners to investigate the extent to which their health care service meets the perceived needs of their client group and pinpoint areas for improvement [22]. The interview will be conducted with consenting patients as close to their care encounter as possible [23]. Data tools are translated into the official languages of the study countries and pilot tested for consistent meaning and relevance to the setting. Data collectors are also able to communicate in local languages. The Satisfaction of Employees in Health Care (SEHC) survey is a validated tool to assess staff satisfaction. It was first developed and validated in a low-income country (Ethiopia) [24] and later successfully validated in a high-income country (USA) [25].

We use a mix of paper and electronic data (ODK [26]) collection tools. Data collectors are trained to minimise error. Tools are piloted before implementing. ODK data is regularly stored and sent to secure servers, as soon as data collectors reach their office base. Data from paper tools is double entered and compared and sent to secure servers. Each data collection tool has its corresponding electronic database that is cleaned and submitted to the analyses. All data is anonymised at the point of data collection or as soon as possible in the data management process. Data is labelled with an

arm code (e.g. 'A' or 'B') without any further information allowing to disclose which data items belong to the intervention or to the control arms, ensuring blinding during data analyses.

Quality will be assured through several mechanisms: piloting of data collection tools; thorough training of field workers; checking missing data related; double, independent data entry from papers into digital databases; early descriptive analyses to detect potential outliers; fieldworkers tracking and supervision.

Data analysis

The analysis will be carried out for each country separately, and based on intention-to-treat.

At baseline, data on population and health facility characteristics (i.e. basic demographic characteristics of population and health workers, professional profile of health workers, health facility size and services) will be produced and presented. If large imbalances are detected at baseline, this information can be used to adjust the effect estimate comparisons [27,28].

The analyses vary for the different primary outcomes due to the unit of measurement and levels of clustering, the type of variable, and whether measurements were taken at baseline and endpoint or endpoint only. We use regression models to allow us to estimate the effect of the outcome while flexibly accounting for these issues and allowing adjustment for potential confounders.

Logistic regression will be used for the binary variables: vaccine adherence is measured by determining whether each vaccine due was received, and treatment appropriateness by whether each treatment was correctly prescribed. Data concordance and diagnostic scope are count variables and may be analysed with Poisson regression, depending on their distribution. The regression model for HW satisfaction will depend on how it is distributed.

The outcomes have different levels of clustering (children or consultations, HW, HF). We will account for these levels of clustering by including random effects in the regression models.

Four of the primary outcomes are measured at baseline and end-line. The effect of the intervention will be estimated using an interaction term between arm and survey in the regression models: ie is

For peer review only

the change in the outcome between baseline and follow-up in the intervention arm different to the change between baseline and follow-up in the control arm. The effect of HW satisfaction, measured only at end-line, will be estimated as the difference between the intervention and control arm.

All estimates for the effect of the intervention will be presented with 95% confidence intervals. The analyses will be carried out using R [29].

Measures to minimise bias

Statistical analyses will be carried out blindly, without knowledge of what health facilities or population in the catchment area belong to the intervention or control groups. Only when the analysis code is considered as definitive and fixed, will results be shared with the wider investigators team and the arms for health facilities and population will be disclosed.

Outcome measurement bias may take place where data from the HIS, which is the focus of the intervention, is used to measure outcomes. However, we will minimise this by assessing population based outcomes at household level.

Contamination (i.e. the intervention affects individuals or units assigned to the control arm) may happen via the exchanges between health workers from health facilities in both arms; for example: in monthly district data quality meetings, managerial meetings; or through inputs from supervisors who influence control health facilities with intervention tips encountered in health facilities of the intervention arms. One mechanism to address this issue is using a district-based cluster randomisation scheme. However, we consider that (i) contamination, despite increasing the awareness of health works in control health facilities, will hardly influence the decision making mechanisms that the HIS intervention focuses on; and (ii) randomisation at the level of district poses additional challenges that are not worth the marginal benefit of reducing a doubtful contamination [30].

The spill over effect (i.e. benefits of the intervention extend beyond their direct recipients) [31] may take place in higher levels of the health systems; e.g. districts data managers and programme

For peer review only

managers may experience the benefits of better structured and more timely data produced in health facilities in the intervention arms. The trial will have no capacity to quantitatively account for spill overs at higher levels of the system, due to the limited number of higher level administrative areas that will be involved in the trial. However, through process indicators, we will consider potential benefits and harms of the intervention at higher levels of the system.

A challenge is the Hawthorne effect (i.e. observer effect). Both health workers in the intervention and in the control sites will have an awareness of being observed as data collection activities will be at the same level of intensity in the two arms. Therefore, there should be no differential effect.

Analyses will be based on the intention-to-treat. It is important to closely monitor if the intervention HFs consistently use the new HIS tools and approaches. The data collection team and the trial monitoring team will check if old forms are still being used in the intervention health facilities. However, we do not expect health facilities to migrate between intervention and control arms, or vice versa, due to feasibility issues. On the other hand, some household members in a given catchment area may decide to seek for health care in a health facility belonging to another trial arm. In these cases, households will be analysed as belonging to the original trial arm.

Discussion

This is one of the very few studies assessing the effects of health systems interventions using experimental study designs [32]. Most of the experimental studies on HIS are circumscribed to specific health care areas (e.g. tuberculosis, vaccination, cardiovascular disease) and very few have a system-wide approach (e.g. PHC) [32]. This is the only experimental study we are aware of, focusing on paper-based HIS.

To date, some modifications to the protocol have taken place. In Côte d'Ivoire, we decided to select study areas close to the research institution base on logistics and practical reasons, instead of selecting an area in the north of the country, where poorer health indicators have been described. In Mozambique, the low density of HF per population implied extremely vast distances between HF and

For peer review only - http://bmjopen.bmj.com/site/about/guidelines.xhtml

this, coupled with the rainy season, made the trial unfeasible in the originally selected Nampula province. After consultations, we decided to move the trial to the province of Inhambane and cancel the household survey. The allocation of HF to the intervention and control arms was completed using random number generation.

Experimental studies for health systems interventions are sometimes dismissed because of their limited capacity to provide reliable explanations of complex health system issues [33]. While we acknowledge these limitations, there is also a need for more robust evidence on the effects of these types of health system interventions [34] and there are also good examples of experimental studies reporting findings that can make it to the policy arena [35]. When embarking on this research, we considered the type of evidence required to contribute to systematic reviews [36], guidance development [37] and eventually recommendations for policy and practice [38]. Furthermore, we have embedded the CRCT in a comprehensive research context which includes systematic reviews of the literature and qualitative research, and we are also looking at explanatory outcomes within the CRCT itself. We believe that this approach will provide a more comprehensive picture of what has happened with the PHISICC tools used by HW and why.

We acknowledge the challenges of carrying out research on health care provided to remote, rural communities (in this case in Sub-Saharan Africa). However, it is only in these remote areas where research about their specific problems and needs can take place. Challenges included long distances, poor conditions of roads, unreliable communications and limited food and accommodation services, all of them to be proactively handled to keep the quality of work and the morale of researchers and collaborators.

The engagement and ownership of partners within this research has also been instrumental in order to plan and implement the CRCT. The intervention actually targets a governmental sub-system (the HIS) for which we required more than permission but also endorsement and active support. We achieved this level of collaboration by ensuring the participation of key stakeholders in key phases of

For peer review only

the whole project, from inception till the implementation of the last phases, through frequent communication and workshops.

We are aware of the current trends towards digitalization of HIS. However, WHO recommendations on the matter are weak since the underlying evidence to support these recommendations is inconsistent [39]. The principles and methodological approaches in PHISICC can be applied to the development of any technological solution, being on paper, digital or mixed. PHISICC, is not mainly about technologies to support data, but rather about a change in paradigm where life-saving decisions by frontline health workers are at the centre of the intervention; facilitating as well the information requirements of higher levels in the health system.

We expect that the results of the trials, both quantitative and qualitative, will be able to inform policies on how to make HIS responsive to providers' decision-making needs, particularly in health services where the most vulnerable live.

Authors' contributions

XBC, AOI, AM, RBY and CAU prepared the proposal for the funding agency, conceived the study and produced the data collection tools. SG ensured the regulatory, ethical and trial monitoring components. AR developed the analytical approaches and made the sample size calculations. RBY, MS and SB adapted the protocol to the context of Côte d'Ivoire and managed the administrative and ethical approvals in the country; AOI, NE, OK, ANN and ABB likewise in Nigeria; AM, SML and GM, in Nampula province (Mozambique); JS and TM adapted the protocol and acquired ethical and administrative clearances for Inhambane province (Mozambique). DB is chair of the PHISICC Technical Advisory Group (TAG) and has coordinated multiple formal and informal inputs. LKK and DB have advised on the adequacy of the study protocol within the overall PHISICC proposal and TAG advices. All country teams participated in PHISICC workshops and ensured that the protocol was suitable to countries realities; developed data collection tools and training materials. They are

For peer review only

responsible for the implementation of the trial in each country. XBC drafted the first version of the manuscript. All authors commented on several versions of the manuscript.

Acknowledgements

Members of the PHISICC Technical Advisory Group, chaired by David Brown: Blanche Anya (WHO AFRO), Abdallah Bchir (former Gavi), Marta Gacic-Dobo (WHO HQ), Richard Greffé (AIGA), Pamela Mitula (WHO AFRO), Sandy Oliver (UCL Institute) and Chris Wolf (BGMF).

Research collaborators: Momade Ali, Celso Belo, Bassirou Bonfoh, Lisa Diallo, John Ferreira, Bernard Guessanbi, Caitlin Jarrett, Inza Koné, Felix Malé, Kouadio M'bra, David O'Donnell, Damaris Rodríguez (Sonder Collective), Melanie Wendland and Meike Zuske (Swiss TPH).

Stakeholders from Côte d'Ivoire, Mozambique and Nigeria, participating in the consultation processes.

Data sharing statement

We report on a protocol of three Cluster Randomised Controlled Trials and therefore no data is available yet. On completion of the trials, access to data will be available from the national research institutions in Côte d'Ivoire, Mozambique and Nigeria, in publications and in funding agency. Data will be made available via an online data repository. Access will be granted following a review of requests by SwissTPH contract officer.

Ethical approvals in Côte d'Ivoire, Mozambique and NIgeria

- Comité National Ethique des Sciences de la Vie et de la Santé (CNESVS), reference: 024-19/MSHP/CNESVS-kp (Côte d'Ivoire)

- Comité Institucional de Bioética para Saúde da Universidade Lúrio, reference: 16.2/Julho/CBISUL/19 (Mozambique)

- Secretary, Government of Cross River State of Nigeria, Ministry of Health, Calabar Health Research Ethics Committee, reference: CRS/MH/HREC/018/Vol. V1/151 (Nigeria)

- Ethikkommission Nordwest- und Zentralschweiz (EKNZ), reference: 2018-01059 (Switzerland).

Funding

Funder by the Bill & Melinda Gates Foundation, grant number INV-010193 / OPP1135947.

For peer review only

Abbreviations

BMGF Bill & Melinda Gates Foundation

CIV Côte d'Ivoire

CRCT Cluster Randomised Controlled Trial

sd Standard deviation

HF Health Facility

HW Health worker

MOZ Mozambique

NGA Nigeria

WHO World Health Organisation

Additional files

- Additional file 1: CONSORT statement checklist.

Competing interests

No, there are no competing interests for any author.

For peer review only

References

1 WHO. Monitoring results with health facility information systems: A technical consultation Glion-sur-Montreux, Switzerland, 11–12 June 2014. WHO 2014.

2 WHO. Health facility & community data toolkit. WHO 2014.

3 Bosch-Capblanch X, Ronveaux O, Doyle V, Remedios V, Bchir A. Accuracy and quality of immunization information systems in forty-one low income countries. Trop Med Int Health. 2009 Jan;14(1):2-10. doi: 10.1111/j.1365-3156.2008.02181.x. PMID: 19152556.

4 Loder E. Promises, promises. BMJ. 2017 Mar 23;356:j1446. doi: 10.1136/bmj.j1446. PMID: 28336513.

5 Wenger N, Méan M, Castioni J, Marques-Vidal P, Waeber G, Garnier A. Allocation of Internal Medicine Resident Time in a Swiss Hospital: A Time and Motion Study of Day and Evening Shifts. Ann Intern Med. 2017 Apr 18;166(8):579-586. doi: 10.7326/M16-2238. Epub 2017 Jan 31. PMID: 28135724.

6 Schulz KF, Altman DG, Moher D, for the CONSORT Group. CONSORT 2010 Statement: updated guidelines for reporting parallel group randomised trials.

7 Campbell MK, Piaggio G, Elbourne DR, Altman DG; for the CONSORT Group. Consort 2010 statement: extension to cluster randomised trials. BMJ. 2012;345:e5661.

8 R Core Team. R: A Language and Environment for Statistical Computing [Internet]. Vienna, Austria; 2020. Available from: https://www.R-project.org/

9 Bostoen K, Chalabi Z. Optimization of household survey sampling without sample frames. Int J Epidemiol. 2006 Jun;35(3):751-5. doi: 10.1093/ije/dyl019. Epub 2006 Feb 15. PMID: 16481364.

10 Johnson, Sandhu, Tyler, "The Next Step for Human-Centered Design in Global Public Health" Stanford Social Innovation Review, Oct. 22, 2019.

11 WHO. Global Vaccine Action Plan 2011-2020. WHO 2013.

12 Carroli G, Villar J, Piaggio G, Khan-Neelofur D, Gülmezoglu M, Mugford M, Lumbiganon P, Farnot U, Bersgjø P; WHO Antenatal Care Trial Research Group. WHO systematic review of randomised controlled trials of routine antenatal care. Lancet. 2001 May 19;357(9268):1565-70. doi: 10.1016/S0140-6736(00)04723-1. PMID: 11377643.

13 Alpern R, Canavan ME, Thompson JT, McNatt Z, Tatek D, Lindfield T, Bradley EH. Development of a brief instrument for assessing healthcare employee satisfaction in a low-income setting. PLoS One.

For peer review only

2013 Nov 5;8(11):e79053. doi: 10.1371/journal.pone.0079053. PMID: 24223878; PMCID: PMC3818514.

14Doll WJ and Torkzadeh G. The measurement of end-user computing satisfaction. MIS Quarterly 1988;12(2):259–74; 19.

15 Ilias A, Yasoa MR, Razaka MZA and Rahmana RA. The study of end-user computing satisfaction (EUCS) on computerised accounting system (CAS) among Labuan F.T. Government sectors: a case study in the responsibility centres. Labuan e-Journal of Muamalat and Society 2007;1:1–13.

16 Arifeen SE, Hoque DM, Akter T, Rahman M, Hoque ME, Begum K, Chowdhury EK, Khan R, Blum LS, Ahmed S, Hossain MA, Siddik A, Begum N, Sadeq-ur Rahman Q, Haque TM, Billah SM, Islam M, Rumi RA, Law E, Al-Helal ZA, Baqui AH, Schellenberg J, Adam T, Moulton LH, Habicht JP, Scherpbier RW, Victora CG, Bryce J, Black RE. Effect of the Integrated Management of Childhood Illness strategy on childhood mortality and nutrition in a rural area in Bangladesh: a cluster randomised trial. Lancet. 2009 Aug 1;374(9687):393-403. doi: 10.1016/S0140-6736(09)60828-X. PubMed PMID: 19647607.

17 Tilahun H, Fekadu B, Abdisa H, Canavan M, Linnander E, Bradley EH, Berman P. Ethiopia's health extension workers use of work time on duty: time and motion study. Health Policy Plan. 2017 Apr 1;32(3):320-328. doi: 10.1093/heapol/czw129. PubMed PMID: 27658649.

18 Hayes RJ, Bennett S. Simple sample size calculation for cluster-randomized trials. Int J Epidemiol. 1999 Apr;28(2):319-26.

19 The DHS program. Demographic and Health surveys. https://dhsprogram.com/.

20 Thayaparan, A. a. (2013). The Patient Satisfaction Questionnaire Short Form (PSQ-18) as an adaptable, reliable, and validated tool for use in various settings. Med Educ Online, 18.

21 The Health Foundation. (2013, June). Measuring patient experience (no.18). Retrieved Nov 6, 2018, from www.health.org.uk: https://www.health.org.uk/publication/measuring-patient-experience.

22 Grogan, S. C. (2000). Validation of a questionnaire measuring patient satisfaction with general practitioner services. Quality in Health Care, 9, 210-215.

23 LaVela, S. L. (2014). Evaluation and measurement of patient experience. Patient Experience Journal, 1(1), Article 5.

24Alpern, R. C. (2013, November). Development of a Brief Instrument for Assessing Healthcare Employee Satisfaction in a Low-Income Setting. PLOS ONE, 8(11), e79053.

For peer review only

25Chang, E. C. (2017). Measuring job satisfaction among healthcare staff in the United States: a confirmatory factor analysis of the Satisfaction of Employees in health Care (SEHC) survey. International Journal for Quality in health Care, 29(2), 262-268.

26 Open Data Kit. https://opendatakit.org/. Accessed 5th February 2021.

27Pocock S, et al. Subgroup analysis, covariate adjustment and baseline comparisons in clinical trial reporting: current practice and problems. Stat Med. 2002;21:2917-30.

28The CONSORT Group. CONSORT Statement [Internet]; item 15.

29 R Core Team. R: A language and environment for statistical computing. R Foundation for Statistical Computing, Vienna, Austria. URL https://www.R-project.org/.

30Torgerson David J. Contamination in trials: is cluster randomisation the answer? BMJ 2001; 322:355.

31 Benjamin-Chung J, Arnold BF, Berger D, Luby SP, Miguel E, Colford JM Jr, Hubbard AE. Spillover effects in epidemiology: parameters, study designs and methodological considerations. Int J Epidemiol. 2018 Feb 1;47(1):332-347. doi: 10.1093/ije/dyx201. PubMed PMID: 29106568; PubMed Central PMCID: PMC5837695.

32 Leon N, Balakrishna Y, Hohlfeld A, Odendaal WA, Schmidt BM, Zweigenthal V, Anstey Watkins J, Daniels K. Routine Health Information System (RHIS) improvements for strengthened health system management. Cochrane Database Syst Rev. 2020 Aug 13;8:CD012012. doi: 10.1002/14651858.CD012012.pub2. PMID: 32803893.

33 Allanson ER, Tunçalp Ö, Vogel JP, Khan DN, Oladapo OT, Long Q, Gülmezoglu AM. Implementation of effective practices in health facilities: a systematic review of cluster randomised trials. BMJ Glob Health. 2017 Jul 20;2(2):e000266. doi: 10.1136/bmjgh-2016-000266. PMID: 29081997; PMCID: PMC5656132.

34 Brown DW, Bosch-Capblanch X, Shimp L. Where Do We Go From Here? Defining an Agenda for Home-Based Records Research and Action Considering the 2018 WHO Guidelines. Glob Health Sci Pract. 2019 Mar 29;7(1):6-11. doi: 10.9745/GHSP-D-18-00431. PMID: 30877139; PMCID: PMC6538131.

35 Joos O, Silva R, Amouzou A, Moulton LH, Perin J, Bryce J, et al. Evaluation of a mhealth data quality intervention to improve documentation of pregnancy outcomes by health surveillance assistants in Malawi: a cluster randomized trial. PloS One 2016;11(1):e0145238. PMID: 26731401. DOI: https://doi.org/10.1371/journal.pone.0145238

For peer review only

36 Langlois EV, Ranson MK, Bärnighausen T, Bosch-Capblanch X, Daniels K, El-Jardali F, Ghaffar A, Grimshaw J, Haines A, Lavis JN, Lewin S, Meng Q, Oliver S, Pantoja T, Straus S, Shemilt I, Tovey D, Tugwell P, Waddington H, Wilson M, Yuan B, Røttingen JA. Advancing the field of health systems research synthesis. Syst Rev. 2015 Jul 10;4:90. doi: 10.1186/s13643-015-0080-9. PMID: 26159806; PMCID: PMC4498528.

37 Bosch-Capblanch X, Lavis JN, Lewin S, Atun R, Røttingen JA, Dröschel D, Beck L, Abalos E, El-Jardali F, Gilson L, Oliver S, Wyss K, Tugwell P, Kulier R, Pang T, Haines A. Guidance for evidence-informed policies about health systems: rationale for and challenges of guidance development. PLoS Med. 2012;9(3):e1001185. doi: 10.1371/journal.pmed.1001185. Epub 2012 Mar 6. PMID: 22412356; PMCID: PMC3295823.

38 WHO. WHO recommendations on home-based records for maternal, newborn and child health. WHO 2018.

39 WHO. Recommendations on digital interventions for health system strengthening. WHO guideline. WHO 2019.

For peer review only

Tables and Figures

Table 1. Outcomes and parameters used to estimate the sample sizes.

	Outcome name	Subjects	Definition	Baseline estimate	Expected change	Comments
1	Vaccination adherence	Children under-1 in the households	Number of vaccines given in the previous calendar year over the number of vaccine due in the same period	75 given per 100 due	Increase of 10 per 100	Vaccines are clustered within children, and children within HFs
2	Data concordance	Recording tools in health facilities	Number of health care events (e.g. vaccinations, antenatal care consultations) recounted in the previous calendar year versus the number of health care events reported in the same time period	7 recounted for each 10 reported [3]	Increase of 2 recounted	A single estimate can be obtained in each HF or by time periods (no clustering)
3	Diagnostic scope	Records of sick child consultations	Number of diagnosis in each sick child consultation during the previous calendar year	1 or 2 per child	30% to 35% with more than 1 diagnosis	Individual consultations are clustered within HF
4	Treatment appropriateness	Records of sick child consultations	Number of treatments correctly prescribed in each sick child consultation during the previous calendar year	Half appropriate over all consultations	Increase to three quarters appropriateness	Individual consultations are clustered within HF
5	Health workforce satisfaction	Health workers	Degree (score) of satisfaction across all health facilities in each arm, with the intervention	5 out of 10	9 out of 10	Maybe two or three health workers can be approached in each health facility

Figure 1. CONSORT diagram: trial flow chart.

Separate file.

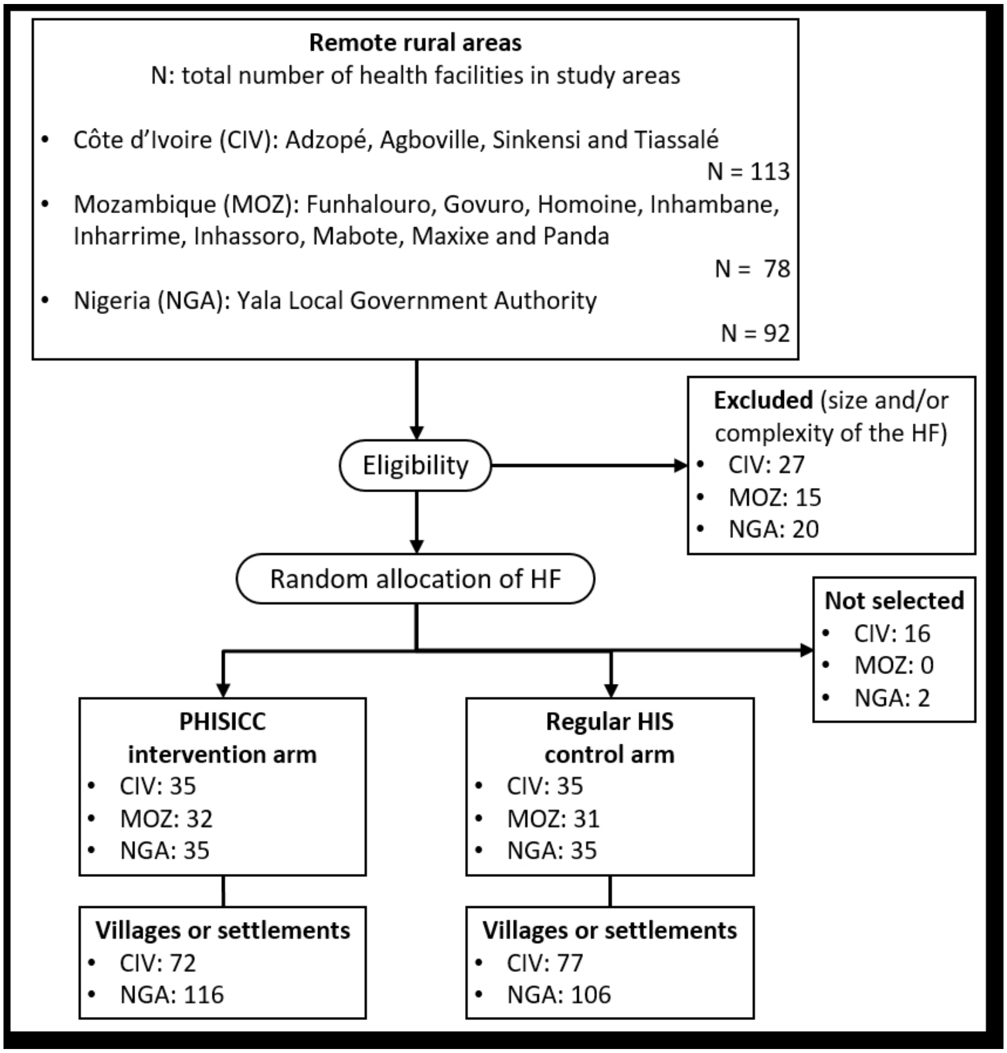

144x150mm (150 x 150 DPI)

Table 1: CONSORT 2010 checklist of information to include when reporting a cluster randomised trial

Section/Topic	Item No	Standard Checklist item	Extension for cluster designs	Page No *
**Title and abstract**				
	1a	Identification as a randomised trial in the title	Identification as a cluster randomised trial in the title	1
	1b	Structured summary of trial design, methods, results, and conclusions	See table 2	5
**Introduction**				
**Background and objectives**	2a	Scientific background and explanation of rationale	Rationale for using a cluster design	4
	2b	Specific objectives or hypotheses	Whether objectives pertain to the cluster level, the individual participant level or both	5
**Methods**				
**Trial design**	3a	Description of trial design (such as parallel, factorial) including allocation ratio	Definition of cluster and description of how the design features apply to the clusters	6
	3b	Important changes to methods after trial commencement (such as eligibility criteria), with reasons		Not applicable
**Participants**	4a	Eligibility criteria for participants	Eligibility criteria for clusters	6
	4b	Settings and locations where the data were collected		6
**Interventions**	5	The interventions for each group with sufficient details to allow replication, including how and when they were actually administered	Whether interventions pertain to the cluster level, the individual participant level or both	8
**Outcomes**	6a	Completely defined pre-specified primary and secondary outcome measures, including how and when they were assessed	Whether outcome measures pertain to the cluster level, the individual participant level or both	10 and Table 1
	6b	Any changes to trial outcomes after the trial commenced, with reasons		Not applicable
**Sample size**	7a	How sample size was determined	Method of calculation, number of clusters(s) (and whether equal or unequal cluster sizes are assumed), cluster size, a coefficient of intracluster correlation (ICC or *k*), and an indication of its uncertainty	12
	7b	When applicable, explanation of any interim analyses and stopping guidelines		Not yet applicable

For peer review only

Section/Topic	Item No	Standard Checklist item	Extension for cluster designs	Page No *
**Randomisation:**				
**Sequence generation**	8a	Method used to generate the random allocation sequence		7, 8
	8b	Type of randomisation; details of any restriction (such as blocking and block size)	Details of stratification or matching if used	Not applicblae
**Allocation concealment mechanism**	9	Mechanism used to implement the random allocation sequence (such as sequentially numbered containers), describing any steps taken to conceal the sequence until interventions were assigned	Specification that allocation was based on clusters rather than individuals and whether allocation concealment (if any) was at the cluster level, the individual participant level or both	7
**Implementation**	10	Who generated the random allocation sequence, who enrolled participants, and who assigned participants to interventions	Replace by 10a, 10b and 10c	7
	10a		Who generated the random allocation sequence, who enrolled clusters, and who assigned clusters to interventions	7
	10b		Mechanism by which individual participants were included in clusters for the purposes of the trial (such as complete enumeration, random sampling)	10
	10c		From whom consent was sought (representatives of the cluster, or individual cluster members, or both), and whether consent was sought before or after randomisation	8
**Blinding**	11a	If done, who was blinded after assignment to interventions (for example, participants, care providers, those assessing outcomes) and how		8
	11b	If relevant, description of the similarity of interventions		Not applicable
**Statistical methods**	12a	Statistical methods used to compare groups for primary and secondary outcomes	How clustering was taken into account	15
	12b	Methods for additional analyses, such as subgroup analyses and adjusted analyses		15

For peer review only

Section/Topic	Item No	Standard Checklist item	Extension for cluster designs	Page No *
**Results**				Not yet applicable (protocol manuscript)
**Discussion**				
**Limitations**	20	Trial limitations, addressing sources of potential bias, imprecision, and, if relevant, multiplicity of analyses		18
**Generalisability**	21	Generalisability (external validity, applicability) of the trial findings	Generalisability to clusters and/or individual participants (as relevant)	18,19
**Interpretation**	22	Interpretation consistent with results, balancing benefits and harms, and considering other relevant evidence		Not yet applicable
**Other information**				
**Registration**	23	Registration number and name of trial registry		1
**Protocol**	24	Where the full trial protocol can be accessed, if available		1
**Funding**	25	Sources of funding and other support (such as supply of drugs), role of funders		20

** Page numbers: as seen in the document "draft_Proof_hi.pdf" (which has 34 pages)*

For peer review only

Does an innovative paper-based health information system (PHISICC) improve data quality and use in primary health care? Protocol of a multi-country, cluster randomised controlled trial in Sub-Saharan African rural settings.

Journal:	*BMJ Open*
Manuscript ID	bmjopen-2021-051823.R1
Article Type:	Protocol
Date Submitted by the Author:	02-Jun-2021
Complete List of Authors:	Bosch-Capblanch, Xavier; Swiss Tropical and Public Health Institute; University of Basel<br>Oyo-Ita, Angela; University of Calabar, Department of Community Medicine<br>Muloliwa, Artur; Lurio University Faculty of Health Sciences<br>Yapi, Richard; Centre Suisse de Recherches Scientifiques en Côte d'Ivoire<br>Auer, Christian ; Swiss Tropical and Public Health Institute; University of Basel<br>Samba, Mamadou; Ministère de la Santé et de l'Hygiène Publique<br>Gajewski, Suzanne; Swiss Tropical and Public Health Institute; University of Basel<br>Ross, Amanda; Swiss Tropical and Public Health Institute; University of Basel<br>Krause, L Kendall; Bill & Melinda Gates Foundation<br>Ekpenyong, Nnette; University of Calabar Teaching Hospital, Department of Community Medicine<br>Nwankwo, Ogonna; University of Calabar, Department of Community Medicine<br>Njepuome, Anthonia; Swiss Tropical and Public Health Institute<br>Lee, Sofia; Swiss Tropical and Public Health Institute<br>Sacarlal, Jahit ; Eduardo Mondlane University, Faculty of Medicine<br>Madede, Tavares; Universidade Eduardo Mondlane<br>Berté, Salimata; Centre Suisse de Recherches Scientifiques en Côte d'Ivoire<br>Matsinhe, Graça; Ministério da Saúde<br>Garba, Abdullahi; National Primary Healthcare Development Agency<br>Brown, David; BCGI LLC / pivot-23.5°
**Primary Subject Heading**:	Health services research
Secondary Subject Heading:	Global health, Health policy, Research methods
Keywords:	Human resource management < HEALTH SERVICES ADMINISTRATION & MANAGEMENT, Organisation of health services < HEALTH SERVICES ADMINISTRATION & MANAGEMENT, Protocols & guidelines < HEALTH SERVICES ADMINISTRATION & MANAGEMENT, Quality in health care <

	HEALTH SERVICES ADMINISTRATION & MANAGEMENT, STATISTICS & RESEARCH METHODS, PRIMARY CARE

SCHOLARONE™ Manuscripts

I, the Submitting Author has the right to grant and does grant on behalf of all authors of the Work (as defined in the below author licence), an exclusive licence and/or a non-exclusive licence for contributions from authors who are: i) UK Crown employees; ii) where BMJ has agreed a CC-BY licence shall apply, and/or iii) in accordance with the terms applicable for US Federal Government officers or employees acting as part of their official duties; on a worldwide, perpetual, irrevocable, royalty-free basis to BMJ Publishing Group Ltd ("BMJ") its licensees and where the relevant Journal is co-owned by BMJ to the co-owners of the Journal, to publish the Work in this journal and any other BMJ products and to exploit all rights, as set out in our licence.

The Submitting Author accepts and understands that any supply made under these terms is made by BMJ to the Submitting Author unless you are acting as an employee on behalf of your employer or a postgraduate student of an affiliated institution which is paying any applicable article publishing charge ("APC") for Open Access articles. Where the Submitting Author wishes to make the Work available on an Open Access basis (and intends to pay the relevant APC), the terms of reuse of such Open Access shall be governed by a Creative Commons licence – details of these licences and which Creative Commons licence will apply to this Work are set out in our licence referred to above.

Other than as permitted in any relevant BMJ Author's Self Archiving Policies, I confirm this Work has not been accepted for publication elsewhere, is not being considered for publication elsewhere and does not duplicate material already published. I confirm all authors consent to publication of this Work and authorise the granting of this licence.

For peer review only

Title

Does an innovative paper-based health information system (PHISICC) improve data quality and use in primary health care? Protocol of a multi-country, cluster randomised controlled trial in Sub-Saharan African rural settings.

Authors

- Xavier Bosch-Capblanch. Corresponding author. Swiss Tropical and Public Health Institute. Basel, Switzerland. And University of Basel, Basel, Switzerland. x.bosch@unibas.ch
- Angela Oyo-Ita. Department of Community Medicine, University of Calabar, Calabar, Nigeria. oyo_ita@yahoo.com
- Artur Manuel Muloliwa. Faculty of Health Sciences, Lúrio University, Nampula, Mozambique. muloliwa@yahoo.com.br
- Richard B Yapi. Centre Suisse de Recherches Scientifiques en Côte d'Ivoire, Abidjan, Côte d'Ivoire. And Centre d'Entomologie Médicale et Vétérinaire, Université Alassane Ouattara, Bouaké, Côte d'Ivoire. richard.yapi@csrs.ci
- Christian Auer. Swiss Tropical and Public Health Institute. Basel, Switzerland. And University of Basel, Basel, Switzerland. christian.auer@swisstph.ch
- Mamadou Samba. Ministère de la Santé et de l'Hygiène Publique, Abidjan, Côte d'Ivoire. And Université Félix Houphouet Boigny, Abidjan, Côte d'Ivoire. samba.mamadou@gmail.com
- Suzanne Gajewski. Swiss Tropical and Public Health Institute. Basel, Switzerland. And University of Basel, Basel, Switzerland. suzanne.gajewski@swisstph.ch
- Amanda Ross. Swiss Tropical and Public Health Institute. Basel, Switzerland. And University of Basel, Basel, Switzerland. amanda.ross@swisstph.ch
- L Kendall Krause. Bill & Melinda Gates Foundation, Seattle, USA. Kendall.Krause@gatesfoundation.org
- Nnette Ekpenyong. Department of Community Medicine, University of Calabar Teaching Hospital, Calabar, Nigeria. And Department of Community Medicine University of Calabar, Calabar, Nigeria. nnekon2015@gmail.com
- Ogonna Nwankwo. Swiss Tropical and Public Health Institute, Basel, Switzerland. And University of Basel, Basel, Switzerland. And Health Policy, Management and Economics Firm, Department of Community. Medicine, University of Calabar, Calabar, Nigeria. ogonna.nwankwo@swisstph.ch
- Anthonia Ngozi Njepuome. Swiss Tropical and Public Health Institute, Abuja, Nigeria. ngonjep@yahoo.com
- Sofia Mandjate Lee. Swiss Tropical and Public Health Institute; Manhiça, Mozambique. sofia_mandjate@yahoo.com.br
- Jahit Sacarlal. Faculty of Medicine, Eduardo Mondlane University, Maputo, Mozambique. jahityash2002@gmail.com
- Tavares Madede. Department of Community Health, Faculty of Medicine, Eduardo Mondlane University, Maputo, Mozambique. tmadede@gmail.com
- Salimata Berté. Centre Suisse de Recherches Scientifiques en Côte d'Ivoire, Abidjan, Côte d'Ivoire. sali.berte@csrs.ci
- Graça Matsinhe. Expanded Program on Immunization, Ministry of Health, Maputo, Mozambique. gmatsinhe@gmail.com.
- Abdullahi Bulama Garba. Director Planning, Research and Statistics, National Primary Health Care Development Agency, Abuja, Nigeria. ab.garba@nphcda.gov.ng.
- David W Brown. Managing Principal, BCGI LLC / pivot-23.5°, North Carolina, USA. david.brown@pivot235.org

For peer review only

Abstract

Introduction

Frontline health workers in remote health facilities are the first contact of the formal health sector and are confronted with life-saving decisions. Health information systems (HIS) support the collection and use of data. However, HIS focus on reporting and are unfit to support decisions. Since data tools are paper-based in most primary health care settings, we have produced an innovative paper-based HIS (PHISICC) using a Human Centred Design approach. We are carrying out a cluster-randomised controlled trial in three African countries to assess the effects of PHISICC compared with the current systems.

Methods and analysis

Study areas are in rural zones of Côte d'Ivoire, Mozambique and Nigeria. Seventy health facilities in each country have been randomly allocated to using PHISICC tools or to continuing to use the regular HIS tools. We have randomly selected households in the catchment areas of each health facility to collect outcomes' data. The baseline survey has been carried out in two of the three countries, the end-line survey is planned for mid-2021. Primary outcomes include data quality and use and coverage of health services and health workers satisfaction; secondary outcomes are additional data quality and use parameters, childhood mortality and additional health workers and clients experience with the system. Just prior to the implementation of the trial we had to relocate the studies in Mozambique and Côte d'Ivoire due to unforeseen logistical issues. The effects of the intervention will be estimated using regression models and accounting for clustering using random effects.

Ethics and dissemination

Ethics committees in Côte d'Ivoire, Mozambique and Nigeria approved the trials. We plan to disseminate our findings, data and research materials among researchers and policy makers. We aim at having our findings included in systematic reviews on health systems interventions and future guidance development on the matter.

Registration

Pan African Clinical Trials Registry - PACTR201904664660639. Registered 01/04/2019, https://pactr.samrc.ac.za/Search.aspx.

For peer review only

Article summary

Strengths and limitations of the study

- These trials assess the effects of improving paper-based health information systems, which are greatly used particularly in remote, rural areas but which are neglected in research.
- The paper-based interventions have been developed using a Human Centred Design approach, with frontline health workers and designers driving the co-creation process.
- Despite the complexity of health systems interventions, we have applied robust experimental methods, together with qualitative research, to assess and understand the effects of the paper-based intervention. Robust evidence on health systems is more likely to gain the credibility of policy-makers and to make it into systematic reviews, guidance development and policy and practice.
- Research targeting frontline health workers in remote, rural areas has to take place where they live and work, which poses serious obstacles in the organisation, management and monitoring of the trials.
- These obstacles, aggravated by the COVID-19 pandemic, have challenged the mobility of the research team, the availability of the intervention in one of the countries and the duration of the trials.

For peer review only

Introduction

Frontline health workers (HW) in remote, rural health facilities (HF) in many countries are the first contact with the formal health sector of the population and they are confronted with life-saving clinical and public health decisions on a daily basis. Decisions are made by exerting a balanced judgment on the information related to health care events, such as making the correct diagnoses or deciding on which vaccinations a child should receive on a given day. In order to properly handle this information, appropriate data support tools and processes are required, referred to as the health information system (HIS); or Routine HIS or Health Management Information System [1]. In reality, though, HIS are primarily designed to report aggregated health events to the higher tiers of the health systems rather than to inform decision-making at the point of care [2].

Increasing pressure by donors and governments to collect more and more data has aggravated the situation, through the proliferation of data support tools that have overloaded frontline health workers compromising their capacity to deliver good quality of care and to delivery good quality data [3], for higher level decision-making.

Promising 'quick fixes', such as the scale up of digital HIS, are taking a long time to implement and face enormous challenges related to infrastructure, equipment and services necessary to run them. Besides, research evidence on the effects of digital solutions remains patchy and inconsistent, even in high-income country settings, where complaints about computerisation of clinical care have been raised [4,5]. Hence, it is very likely that paper tools will remain a primary, if not unique, data support mechanism particularly in remote, rural HF in many countries.

PHISICC (Paper-based Health Information System in Comprehensive Care) is a multi-year, multi-country, mixed-methods research project that aims at producing and testing an innovative paper-based HIS to improve data quality and use, decision making and health outcomes, at Primary Health Care (PHC). It is being carried out in selected areas within Côte d'Ivoire, Mozambique and Nigeria. The project started in 2015, producing a systematic review on the effects of HIS interventions [6,7]

For peer review only - http://bmjopen.bmj.com/site/about/guidelines.xhtml

and a framework synthesis on how HIS are understood in the literature in order to learn from past experiences in HIS developments. This global evidence was coupled with studies to characterise the existing HIS in the three countries, to understand how health workers interact with the HIS and to identify entry points for HIS design improvements. With these bodies the research team was well equipped to engage into a Human Centred Design (HCD) co-creative process with frontline HW to design an innovative HIS (PHISICC). See Figure 1 for an illustration of the structure, processes and evidence flow within PHISICC.

The impact of the PHISICC HIS on data quality and use, quality of health care and HW perceptions is being assessed concurrently in rural areas in the three countries. We describe the design of the trial here, consistent with CONSORT reporting guidelines [8] and the extension for cluster randomised controlled trials (CRCT) [9]; see Additional file 1.

Methods

Aim

The aim of the trial is to address the research question: what are the effects of an innovative paper-based HIS (PHISICC) on data use and quality, quality of health and HW perceptions compared with the current HIS, in rural PHC settings?

Patient and public involvement

There was no public or patient involvement in the design of the study or selection of study areas because the intervention being assessed in these trials target health care providers and decision-makers, rather than patients or the public in general. Population in the catchment area of selected health facilities, potentially using their services, were only approached in order to assess the studies outcomes.

On the other hand, we have involved health systems stakeholders and frontline health workers. Ministries of Health at several levels participated in the preparation of the research proposal

For peer review only

(personal consultations), in the characterisation of health information systems that preceded the trials (countries workshops), and throughout all project components (additional workshops, newsletters and personal communication). Frontline health workers in the three countries have co-created the intervention (i.e. paper based tools) through workshops, personal feedback and piloting under real live conditions. Some of them are part of the research team and co-authoring this manuscript.

Study design

The study is a CRCT in each of the three countries. In each setting, 70 health facilities are randomised to intervention or control (35 per arm). The intervention arm HF use the new PHISICC tools (substituting the usual HIS tools) and the control arm HF use the regular HIS tools. The trial is implemented in the real life contexts of HF carrying out their usual duties.

The trials started between the end of 2019 and beginning of 2020, depending on the country, when the intervention was installed and the baseline surveys carried out. Data collection will last until mid-2021.

Study areas

Ministries of Health (MOH) officials in several countries were contacted before submitting the proposal to the funding agency in order to explore the willingness to engage in a project focusing on paper-based tools. Officials in several countries rejected the offer on the grounds of upcoming digitalisation plans of the HIS in the country. We partnered with MOH that found the research relevant to their context in three countries.

In each country, the eligibility criteria of study areas were that they had to belong to the operational area of research partners; contain a large enough number of health facilities and their catchment population; include vulnerable population (e.g. with low vaccination coverage, high childhood mortality); and be comparatively neglected in terms of infrastructure and services. We excluded areas with concurrent research or other types of activities that could conflict with the CRCT (such as

For peer review only

the co-existence of another health-related study, massive developments in infrastructure or activities involving migration of the population, such as temporary work sites or changes in working sites) and areas with threats to safety or security that could jeopardise research activities.

The study areas are located in Adzopé, Agboville, Tiassalé and Sikensi districts (Côte d'Ivoire); in Funhalouro, Govuro, Homoine, Inhambane, Inharrime, Inhassoro, Mabote, Maxixe and Panda (Inhambane province, Mozambique); and in Yala Local Government Authority (Cross River State, Nigeria).

Eligibility of health facilities

The intervention is implemented at the HF level. The eligibility criteria of the HF were that they had to be located in the study areas, belong to the governmental health sector and their main activity should be the delivery of PHC services. HF were excluded if they had specialised clinical services, inpatients, physicians providing care or with plans for staff turn-over involving intervention and control HF.

A 'master list' of eligible health facilities was prepared based on information provided by the MOH across all study areas. We aimed at selecting 70 of the eligible HF in each country, using simple random techniques in R [10]. See in Figure 2 the selection and allocation trial flow chart.

Allocation and blinding

Allocation of the 70 HF per country into the intervention and control arms took place in a formal event, gathering research partners and MOH officials to offer transparency and promote study ownership by local and national authorities. Equally sized, folded pieces of paper with the names and codes of included HF written on them were introduced in an opaque receptacle where they were manually and blindly mixed. A second receptacle contained two equally sized pieces of paper, one with the word 'intervention' and another one with the word 'control'. A selected person in the meeting, not belonging to the research team, extracted one piece of paper at a time to reach half the number of included HF. Then, a paper was extracted from the second receptacle to assign those HF

For peer review only

to the intervention or control arms. The rest of the papers were extracted as well to verify completeness and no duplication of names, and those HF assigned to the other arm.

Once HF were selected, all villages or settlements for each health facility catchment area were listed and three in each catchment area were selected. In practice, we selected all villages because the numbers were below (in Côte d'Ivoire) or just above (in Nigeria) the needs. For each village, we used Google satellite maps to identify and geo-locate every visible roof. Where there were many houses per village (roughly, more than fifty or so), a researcher would mark four points in the map slightly beyond the northernmost, southernmost, easternmost and westernmost roofs seen and 30 random points were selected within that square. From the mapped points, 10 per village (with 10 more acting as reserve) were randomly selected and marked on another map used in the field for data collectors to approach households. Where technical problems impeded this approach in a given village, a field supervisor would rotate a bottle on the floor towards the centre of the village and would select at random 10 households in the direction pointed by the bottle, from the outer limit of the village till the centre [11].

Blinding is only feasible for the research team members carrying out the CRCT data collection and the analyses of the CRCT findings. The intervention (i.e. paper tools) are by design very different from the existing system and it is not possible to blind participants or principal researchers.

We already had the agreement of the MOH and selected HF compliant with the inclusion criteria were provided with the intervention shortly after completing the baseline data collection. Therefore, recruitment as such took place at the same time of the allocation of HF into intervention and control arms.

The intervention

The PHISICC paper-based intervention is a full set of paper-based tools to support decision-making by frontline HW. These are the only tools to be used by HW in the intervention arm. The PHISICC tools encompass the whole system (i.e. recording and reporting) and all clinical and public health care

For peer review only

areas and are characterised by: a common visual language (e.g. spaces for digits and text), standardised formats across health care areas; support to critical data items (e.g. respiratory rate in infants); graphic artefacts to distinguish severity degrees of signs or symptoms; documentation of diagnoses and treatment decisions; and aides memoires, among others.

The PHISICC tools have been developed from May 2017 till June 2019, including production, using a Human Centred Design approach [12]. A strength of the Human Centred Design approach is its ability to unlock the user's perspective so that designers can build solutions that are fully reality-based and work well. Co-creation groups were formed in each country with researchers, staff from partner institutions and healthcare workers, led by a team of professional designers. Research team members supervised and coordinated exclusively the feedback on the contents of the tools, to ensure compliance with each country clinical guidelines. At the outset of the process, the design focused on three health care areas (i.e. antenatal care, vaccination and sick child) and slowly extended the new visual language to other health care areas. Initial workshops served to brainstorm on problems and potential design solutions, without any other rule than being comprehensive and not rejecting a single idea. Designers, then, formalised some of the most promising solutions and a first round of exchanges within the co-creation team was used to address misinterpretations or inconsistencies. There were two in-the-field testing rounds in Mozambique, two in Côte d'Ivoire and three in Nigeria and uncountable exchanges through teleconferences and email, in-between. The prototypes were considered final when no errors were detected, were compliant with data needs in each country and comments from the field could not be accommodated in the design concept or there was no consensus on minor or formal issues being raised.

The PHISICC tools have been produced in French for Côte d'Ivoire, in Portuguese for Mozambique and in English for Nigeria, which are the official languages used in the health systems in the three countries. They include the official logo of the MOHs. Health care areas covered include: family planning, antenatal care, including tetanus toxoid vaccination, delivery, post-natal care, vaccination,

For peer review only

sick child, adults outpatient consultation, tuberculosis diagnosis and treatment, and HIV. Referral forms were also designed.

The PHISICC tools have three sub-components: registers, tallies and reports. Registers are formed by seven DIN-A3 and one DIN-A4 (for referrals) book covering all health care areas except for tuberculosis treatment, for which DIN-A3 cards where used. Register books have 100, 200 or 400 pages depending on the country and health care area. They are used to record individual clients' data for each health care event, either of clinical or public health nature. Some register books have clinical notes at the very beginning, as 'aide memoires', and an example of a filled-in form, to assist HW when doubting how to proceed.

Tallies are DIN-A3 single sheets which contain a list of the indicators to be transferred to higher levels of the health system, with a series of small ovals, grouped in fives, to mark with tally sticks with a pen. In contrast to the current systems that have no tallies or only for vaccination, tallies were created for all health care areas. In the middle-right side of the tally, a column accommodates cells aligned with the ovals to insert the count for each indicator; and in the far right of the sheet there is a replica of the count column, separated with a perforated line, which is detached and sent, as part of the monthly report to the higher level in the health system.

While current HIS tools are consistently organised in tabular formats and books, where each clinical event is recorded in a row and each variable (e.g. age, gender, HIV status, diagnosis) in a column, PHISICC tools incorporated several innovations; in summary: a visual language to guide the clinical decisions of health workers based on severity (i.e. if it is recorded that a child has convulsions, a visual artefact indicates severity), more space for clinical data (e.g. vital signs), inclusion of all critical information to assess patients (e.g. obstetric history, gestational age, fundus height, breath rate in infants), consolidation of information of all antenatal care visits for a single pregnant woman in the same page, among many other formal and contents improvements, including improved aesthetics. We aimed at creating "a system" (not just some tools) focusing on decision making by frontline health workers. The epidemiological and public health contexts in the three countries are similar, as

For peer review only - http://bmjopen.bmj.com/site/about/guidelines.xhtml

confirmed by the similarities in the existing HIS between the three. The visual language and the recording forms where common to the three countries because clinical decisions are common to the three; although forms allowed for specific tests or treatments. The reporting component was adapted to each country set of indicators, although the visual language and reporting processes were harmonised.

During three or four days, HW were trained on HIS before the start of the trial. In the intervention arm they were trained on the PHISICC tools; and the control arm received a refresher training about the regular tools, during the same number of days.

Additionally, given that the regular tools already contained information on past vaccination history of children still to complete their vaccination schedule, we created a mechanism to retrieve data of children's vaccination status to transcribe into the new vaccination register book in the intervention arm ('system transition').

Tools were endorsed by MOH, printed in local printing companies and distributed to HW at the end of the training sessions. A digital spreadsheet was created to monitor consumption and order additional tools to cover health facility needs during the life of the trial.

Outcomes

There are five primary outcomes (Table 1). Vaccination adherence is defined as the total number of vaccine doses given in the correct time interval to children in households in the health facilities catchment villages of those over the total number of vaccine doses that should have been given during the same period. Antenatal care visits uptake will also be considered depending on the expected number of pregnancies in the study areas. Both are used as proxies for health outcomes in terms of protection against disease [13] and prevention of pregnancy complications [14] and are assessed in a random sample of households in the health facilities catchment areas. Data concordance is defined as the level of agreement of HIS indicators between (i) records of health care events (re-counts), (ii) tallies (re-counts) and (iii) reports (aggregated data to higher levels of the

For peer review only

system) [3]. The appropriateness of treatment will be measured using the diagnostics scope in the sick child (i.e. number of different diagnoses per sick child consultation); and treatment appropriateness (i.e. number of prescribed treatments that are supported by a documented diagnosis). These outcomes will be assessed in a random sample of records and corresponding reports during the last four months of the study period. Health workers satisfaction will be assessed in all health workers in included health facilities using a standardised questionnaire [15,16,17]. While the intervention targets HF, some of the outcomes are measured at the level of HF, and some from patients clustered within HF catchment areas.

Secondary outcomes are classified under the following domains: data quality, data use, mortality, HW experience, client experience and resource consumption:

- Data quality, assessed in a sample of records
    - Completeness of recording and reporting in specific forms; i.e. prevalence of unduly missing data items; partograph used;
    - accuracy of recorded figures in comparison to real events (e.g. physical counting of commodities, such as number of 500mg Paracetamol tablets as recorded versus number of 500mg Paracetamol tablets as counted);
    - timeliness of reporting, as documented by time stamps in forms;
    - loss of data or data which does not reach the next upper administrative level.

For peer review only

- Data use, assessed in a sample of records
  - in terms of knowledge (e.g. vaccines due based on date of birth; weight for length assessments);
  - cases of different conditions properly treated in (e.g. diarrhoea cases given oral rehydration therapy according to national guidelines; pneumonia cases given appropriate antibiotic according to national guidelines;
  - public health decisions: availability of lost to follow up lists or plans for vaccination, tuberculosis and or HIV/AIDS treatment control;
  - occurrence of stock outs of essential drugs.
- Overall under-5s mortality and under-5s mortality excluding peri-natal mortality [18], in a sample of households in health facilities catchment areas.
- Health workers' 'human experience' and satisfaction (all health workers).
- District health information officers' 'human experience' (selected health care programme managers).
- Clients' 'human experience' and satisfaction, in a sample of households in health facilities catchment areas.
- Resources consumption (e.g. time use, costs)
  - intervention costs: tools, training, start-up;
  - time used for recording and reporting (e.g. time-motion study) [19];
  - cost-effectiveness per unit of additional improvement in outcomes of interest.

It is worthwhile to note that outcomes that do not relate to data quality and use will be assessed using additional data collection tools (e.g. survey questionnaires), which are the same for intervention and control health facilities. Hence, the effects of the intervention cannot be attributed to the changes in performance of the paper tools routinely used to record health care events in intervention and control health facilities, which are different by design.

For peer review only

In addition, we will consider 'explanatory outcomes' that will help to understand how the measured effects have taken place and why. We will look at the details of the interplay between the intervention, the system, the users and the context. Process indicators will be based on the documented activities that have taken place, from the conception of the intervention, up to its implementation, monitoring and evaluation. Process indicators may include: intervention set up and implementation, monitoring of the use of the intervention, special activities targeted at vulnerable populations, district reactions related to the intervention, handling of data coming from the new system, sustainability based on costs information and perceptions, alignment with national health policies and donor priorities. We will also explore health care services characteristics looking at generic indicators from health facilities, such human resources profiles and relations with the communities, population characteristics and system and context characteristics captured in early stages of the project, where data are available.

Sample size calculations

The required sample sizes for each primary outcome were determined using simulation to incorporate the clustering easily (Table 1) and to take the baseline and end-line surveys into account. Briefly, we simulated 1000 trials with variation between them caused by drawing different samples from the same distributions. We then used the regression models detailed in the data section to analyse each of the simulated trials and estimate the power as the proportion of trials which detected the effect of the intervention as significant. The simulation code was written in R (supplementary files 1 and 2).

For each country, we require the probability of α, a type I error (rejecting the null hypothesis when it is actually true) to be less 0.05 and the power to be at least 80%.

For vaccination adherence, using a sample size of 35 HF per arm, we would have 80% power in each country to detect as significant a difference between a proportion of due vaccines given from 75% in the control to 85% in the intervention arm, assuming one child per household, 30 households per HF

and a between-HF variation equivalent to a k of 0.25, where k is equal to the standard deviation divided by the mean. The value of k is unknown, but was chosen in line with general observations by Hayes and Bennet [20].

For data quality outcomes, with 35 HF per arm we would be able to detect as significant a difference from a ratio of 0.7 (reported : recorded) vaccinations in the control arm to 0.8 with the intervention with 80% power, assuming 100 recorded vaccinations per HF and a standard deviation of 0.25 in the ratios between HF.

In terms of diagnostic scope, we would be able to detect an increase in the proportion of child-visits with more than one diagnosis from 30% to 35% with 80% power with 35 HF per arm, 60 records per HF and assuming a k of 0.25.

We would be able to detect as significant an increase from 50% of treatments having a corresponding appropriate diagnosis to 60% with 80% power assuming 35 HF per arm, 1 treatment per child, 25 children per HF and variation between HF corresponding to k = 0.25.

For the outcome related to health workers' satisfaction, we would be able to detect as significant an increase from 50% of health workers satisfied to 75%, with 80% power assuming 35 HF, three health workers per HF and a variation between HF equivalent to k = 0.25. Since this variable is measured in the end-line survey only, we used the formula in Hayes and Bennet [20].

In summary, in each country we require 35 HF per arm, three HW per HF, 100 vaccination records per HF, 60 sick child records per health facility and 30 children per health facility catchment area.

Data collection and management

Data collection took place at baseline and will take place again at the end of the study. Data is collected from health facilities, from the households in the catchment areas of the included health facilities and also from district offices.

For data quality and data use outcomes, HF registers, tallies and reports will be scrutinised. For population based outcomes, we carry out household surveys at baseline and at end-line. We use

For peer review only

standard approaches for these types of surveys [21]. Households are visited, the research project is briefly introduced and consent requested. Ideally, mothers of alive children or women in child-bearing age were interviewed in order to obtain information on living children (i.e. vaccination history) and death events, respectively, using home-based records if available and accessible. Patients' satisfaction will be assessed using the PSQ-18 satisfaction questionnaire [22,23,24]. Essentially, the tool enables practitioners to investigate the extent to which their health care service meets the perceived needs of their client group and pinpoint areas for improvement [24]. The interview will be conducted with consenting patients as close to their care encounter as possible [25]. Data tools are translated into the official languages of the study countries and pilot tested for consistent meaning and relevance to the setting. Data collectors are also able to communicate in local languages. The Satisfaction of Employees in Health Care (SEHC) survey is a validated tool to assess staff satisfaction. It was first developed and validated in a low-income country (Ethiopia) [26] and later successfully validated in a high-income country (USA) [27].

We use a mix of paper and electronic data (ODK [28]) collection tools. Data collectors are trained to minimise error. Tools are piloted before implementing. ODK data is regularly stored and sent to secure servers, as soon as data collectors reach their office base. Data from paper tools is double entered and compared and sent to secure servers. Each data collection tool has its corresponding electronic database that is cleaned and submitted to the analyses. All data is anonymised at the point of data collection or as soon as possible in the data management process. Data is labelled with an arm code (e.g. 'A' or 'B') without any further information allowing to disclose which data items belong to the intervention or to the control arms, ensuring blinding during data analyses.

Quality will be assured through several mechanisms: piloting of data collection tools; thorough training of field workers; checking missing data related; double, independent data entry from papers into digital databases; early descriptive analyses to detect potential outliers; fieldworkers tracking and supervision.

For peer review only

Data analysis

The analysis will be carried out for each country separately, and based on intention-to-treat.

Baseline population and health facility characteristics (i.e. basic demographic characteristics of population and health workers, professional profile of health workers, health facility size and services) will be summarised. If large imbalances are observed at baseline, the variables can be used to adjust the effect estimate comparisons [29,30].

The analyses vary for the different primary outcomes due to the unit of measurement and levels of clustering, the type of variable, and whether measurements were taken at baseline and endpoint or endpoint only. We use regression models to allow us to estimate the effect of the outcome while flexibly accounting for these issues and allowing adjustment for potential confounders.

Logistic regression will be used for the binary variables: vaccine adherence is measured by determining whether each vaccine due was received, and treatment appropriateness by whether each treatment was correctly prescribed. Data concordance and diagnostic scope are count variables and may be analysed with Poisson regression, depending on their distribution. The regression model for HW satisfaction will depend on how it is distributed.

The outcomes have different levels of clustering (children or consultations, HW, HF). We will account for these levels of clustering by including random effects in the regression models.

Four of the primary outcomes are measured at baseline and end-line. The effect of the intervention will be estimated using an interaction term between arm and survey in the regression models: i.e. is the change in the outcome between baseline and follow-up in the intervention arm different to the change between baseline and follow-up in the control arm? The effect of HW satisfaction, measured only at end-line, will be estimated as the difference between the intervention and control arm.

All estimates for the effect of the intervention will be presented with 95% confidence intervals. The analyses will be carried out using R [31].

For peer review only

Measures to minimise bias

Statistical analyses will be carried out blindly, without knowledge of what health facilities or population in the catchment area belong to the intervention or control groups. Only when the analysis code is considered as definitive and fixed, will results be shared with the wider investigators team and the arms for health facilities and population will be disclosed.

Outcome measurement bias may take place where data from the HIS, which is the focus of the intervention, is used to measure outcomes. However, we will minimise this by assessing population based outcomes at household level.

Contamination (i.e. the intervention affects individuals or units assigned to the control arm) may happen via the exchanges between health workers from health facilities in both arms; for example: in monthly district data quality meetings, managerial meetings; or through inputs from supervisors who influence control health facilities with intervention tips encountered in health facilities of the intervention arms. One mechanism to address this issue is using a district-based cluster randomisation scheme. However, we consider that (i) contamination, despite increasing the awareness of health works in control health facilities, will hardly influence the decision making mechanisms that the HIS intervention focuses on; and (ii) randomisation at the level of district poses additional challenges that are not worth the marginal benefit of reducing a doubtful contamination [32].

The spill-over effect (i.e. benefits of the intervention extend beyond their direct recipients) [33] may take place in higher levels of the health systems; e.g. district data managers and programme managers may experience the benefits of better structured and more timely data produced in health facilities in the intervention arms. The trial will have no capacity to quantitatively account for spill overs at higher levels of the system, due to the limited number of higher level administrative areas that will be involved in the trial. However, through process indicators, we will consider potential benefits and harms of the intervention at higher levels of the system.

For peer review only

A challenge is the Hawthorne effect (i.e. observer effect). Both health workers in the intervention and in the control sites will have an awareness of being observed as data collection activities will be at the same level of intensity in the two arms. Therefore, there should be no differential effect.

Analyses will be based on the intention-to-treat. It is important to closely monitor if the intervention HFs consistently use the new HIS tools and approaches. The data collection team and the trial monitoring team will check if old forms are still being used in the intervention health facilities. However, we do not expect health facilities to migrate between intervention and control arms, or vice versa, due to feasibility issues. On the other hand, some household members in a given catchment area may decide to seek for health care in a health facility belonging to another trial arm. In these cases, households will be analysed as belonging to the original trial arm.

Ethics and dissemination

Ethics committees in Côte d'Ivoire, Mozambique and Nigeria approved the study in their respective countries. To date, some modifications to the protocol have taken place. In Côte d'Ivoire, we decided to select study areas close to the research institution base on logistics and practical reasons, instead of selecting an area in the north of the country, where poorer health indicators have been described. In Mozambique, the low density of HF per population implied extremely vast distances between HF and this, coupled with the rainy season, made the trial unfeasible in the originally selected Nampula province. After consultations, we decided to move the trial to the province of Inhambane and cancel the household survey. The allocation of HF to the intervention and control arms was completed using random number generation.

We plan to disseminate the findings of the trials as one of the few examples of studies assessing the effects of health information systems interventions using experimental study designs [34]. Most of the experimental studies on HIS are circumscribed to specific health care areas (e.g. tuberculosis, vaccination, cardiovascular disease) and very few have a system-wide approach (e.g. PHC) [34]. Experimental studies for health systems interventions are sometimes dismissed because of their

For peer review only

limited capacity to provide reliable explanations of complex health system issues [35]. While we acknowledge these limitations, there is also a need for more robust evidence on the effects of these types of health system interventions [36] and there are also good examples of experimental studies reporting findings that can make it to the policy arena [37]. When embarking on this research, we considered from the outset the type of evidence required to be disseminated and included into systematic reviews [38], guidance development [39] and eventually recommendations for policy and practice [40].

We acknowledge the challenges of carrying out research on health care provided to remote, rural communities (in this case in Sub-Saharan Africa). However, it is only in these remote areas where research about their specific problems and needs can take place. Challenges included long distances, poor conditions of roads, unreliable communications and limited food and accommodation services, all of them to be proactively handled to keep the quality of work and the morale of researchers and collaborators. We expect that the dissemination of findings in meetings, conferences and publications will contribute to a better understanding of what it takes to make research in challenging contexts.

The engagement and ownership of partners within this research has also been instrumental in order to plan and implement the CRCT. The intervention actually targets a governmental sub-system (the HIS) for which we required more than permission but also endorsement and active support. We achieved this level of collaboration by ensuring the participation of key stakeholders in key phases of the whole project, from inception till the implementation of the last phases, through frequent communication and workshops. The PHISICC programme includes targeted activities to keep decision-makers engaged and we are planning to share the findings through workshops as well as online and face-to-face events to disseminate the lessons learned from the trial and the whole research.

For peer review only

We also expect that the dissemination of our findings among partners and competitors will contribute to the current debates on the digitalisation of health information systems. WHO recommendations on the matter are weak since the underlying evidence to support these recommendations is inconsistent [41]. The principles and methodological approaches in PHISICC can be applied to the development of any technological solution, being on paper, digital or mixed.

Finally, we expect that the results of the trials, both quantitative and qualitative, will be able to inform policies on how to make HIS responsive to providers' decision-making needs, particularly in health services where the most vulnerable live.

For peer review only

Authors' contributions

XBC, AOI, AM, RBY and CAU prepared the proposal for the funding agency, conceived the study and produced the data collection tools. SG ensured the regulatory, ethical and trial monitoring components. AR developed the analytical approaches and made the sample size calculations. RBY, MS and SB adapted the protocol to the context of Côte d'Ivoire and managed the administrative and ethical approvals in the country; AOI, NE, ON, ANN and ABG likewise in Nigeria; AM, SML and GM, in Nampula province (Mozambique); JS and TM adapted the protocol and acquired ethical and administrative clearances for Inhambane province (Mozambique). DB is chair of the PHISICC Technical Advisory Group (TAG) and has coordinated multiple formal and informal inputs. LKK and DB have advised on the adequacy of the study protocol within the overall PHISICC proposal and TAG advices. All country teams participated in PHISICC workshops and ensured that the protocol was suitable to countries realities; developed data collection tools and training materials. They are responsible for the implementation of the trial in each country. XBC drafted the first version of the manuscript. All authors commented on several versions of the manuscript.

Acknowledgements

Members of the PHISICC Technical Advisory Group, chaired by David Brown: Blanche Anya (WHO AFRO), Abdallah Bchir (former Gavi), Marta Gacic-Dobo (WHO HQ), Richard Greffé (AIGA), Pamela Mitula (WHO AFRO), Sandy Oliver (UCL Institute) and Chris Wolf (BMGF).

Research collaborators: Momade Ali, Celso Belo, Bassirou Bonfoh, Lisa Diallo, John Ferreira, Bernard Guessanbi, Caitlin Jarrett, Inza Koné, Felix Malé, Kouadio M'bra, David O'Donnell, Damaris Rodríguez (Sonder Collective), Melanie Wendland and Meike Zuske (Swiss TPH).

Stakeholders from Côte d'Ivoire, Mozambique and Nigeria, participating in the consultation processes.

For peer review only

Data sharing statement

We report on a protocol of three Cluster Randomised Controlled Trials and therefore no data is available yet. On completion of the trials, access to data will be available from the national research institutions in Côte d'Ivoire, Mozambique and Nigeria, in publications and in the funding agency. Individual participants' data on vaccination and antenatal care outcomes as well as health workers and users perceptions will be anonymised and made available via an online data repository for any purpose and access will be granted following a review of requests by the Swiss TPH contract officer. Data, with DOIs, will be made available during the second semester of 2021.

Available documents include study protocols, analytical plan, informed consent forms and analytical code.

Ethical approvals in Côte d'Ivoire, Mozambique and Nigeria

- Comité National Ethique des Sciences de la Vie et de la Santé (CNESVS), reference: 024-19/MSHP/CNESVS-kp (Côte d'Ivoire)

- Comité Institucional de Bioética para Saúde da Universidade Lúrio, reference: 16.2/Julho/CBISUL/19 (Mozambique)

- Secretary, Government of Cross River State of Nigeria, Ministry of Health, Calabar Health Research Ethics Committee, reference: CRS/MH/HREC/018/Vol. V1/151 (Nigeria)

- Ethikkommission Nordwest- und Zentralschweiz (EKNZ), reference: 2018-01059 (Switzerland).

Funding

Funded by the Bill & Melinda Gates Foundation, grant number INV-010193 / OPP1135947.

For peer review only

Abbreviations

BMGF Bill & Melinda Gates Foundation

CRCT Cluster Randomised Controlled Trial

sd Standard deviation

HF Health Facility

HW Health worker

WHO World Health Organisation

Additional files

- Additional file 1: CONSORT statement checklist.

Competing interests

No, there are no competing interests for any author.

For peer review only

References

1 WHO. Monitoring results with health facility information systems: A technical consultation Glion-sur-Montreux, Switzerland, 11–12 June 2014. WHO 2014.

2 WHO. Health facility & community data toolkit. WHO 2014.

3 Bosch-Capblanch X, Ronveaux O, Doyle V, Remedios V, Bchir A. Accuracy and quality of immunization information systems in forty-one low income countries. Trop Med Int Health. 2009 Jan;14(1):2-10. doi: 10.1111/j.1365-3156.2008.02181.x. PMID: 19152556.

4 Loder E. Promises, promises. BMJ. 2017 Mar 23;356:j1446. doi: 10.1136/bmj.j1446. PMID: 28336513.

5 Wenger N, Méan M, Castioni J, Marques-Vidal P, Waeber G, Garnier A. Allocation of Internal Medicine Resident Time in a Swiss Hospital: A Time and Motion Study of Day and Evening Shifts. Ann Intern Med. 2017 Apr 18;166(8):579-586. doi: 10.7326/M16-2238. Epub 2017 Jan 31. PMID: 28135724.

6 Bosch-Capblanch X, Zuske M, Auer C, Amadasoun S. Effects of interventions to improve 'health information use systems' on the quality and use of information. National Institute for Health Research. PROSPERO - https://www.crd.york.ac.uk/prospero/display_record.php?RecordID=35218.

7 Bosch-Capblanch X, Zuske M, Auer C. Making information support systems relevant to decision-makers: an effectiveness systematic review and a framework synthesis. Abstracts of the 25th Cochrane Colloquium, Edinburgh, UK. Cochrane Database of Systematic Reviews 2018;(9 Suppl 1). https://doi.org/10.1002/14651858.CD201801.

8 Schulz KF, Altman DG, Moher D, for the CONSORT Group. CONSORT 2010 Statement: updated guidelines for reporting parallel group randomised trials.

9 Campbell MK, Piaggio G, Elbourne DR, Altman DG; for the CONSORT Group. Consort 2010 statement: extension to cluster randomised trials. BMJ. 2012;345:e5661.

10 R Core Team. R: A Language and Environment for Statistical Computing [Internet]. Vienna, Austria; 2020. Available from: https://www.R-project.org/

11 Bostoen K, Chalabi Z. Optimization of household survey sampling without sample frames. Int J Epidemiol. 2006 Jun;35(3):751-5. doi: 10.1093/ije/dyl019. Epub 2006 Feb 15. PMID: 16481364.

12 Johnson, Sandhu, Tyler, "The Next Step for Human-Centered Design in Global Public Health" Stanford Social Innovation Review, Oct. 22, 2019.

13 WHO. Global Vaccine Action Plan 2011-2020. WHO 2013.

For peer review only

14 Carroli G, Villar J, Piaggio G, Khan-Neelofur D, Gülmezoglu M, Mugford M, Lumbiganon P, Farnot U, Bersgjø P; WHO Antenatal Care Trial Research Group. WHO systematic review of randomised controlled trials of routine antenatal care. Lancet. 2001 May 19;357(9268):1565-70. doi: 10.1016/S0140-6736(00)04723-1. PMID: 11377643.

15 Alpern R, Canavan ME, Thompson JT, McNatt Z, Tatek D, Lindfield T, Bradley EH. Development of a brief instrument for assessing healthcare employee satisfaction in a low-income setting. PLoS One. 2013 Nov 5;8(11):e79053. doi: 10.1371/journal.pone.0079053. PMID: 24223878; PMCID: PMC3818514.

16 Doll WJ and Torkzadeh G. The measurement of end-user computing satisfaction. MIS Quarterly 1988;12(2):259–74; 19.

17 Ilias A, Yasoa MR, Razaka MZA and Rahmana RA. The study of end-user computing satisfaction (EUCS) on computerised accounting system (CAS) among Labuan F.T. Government sectors: a case study in the responsibility centres. Labuan e-Journal of Muamalat and Society 2007;1:1–13.

18 Arifeen SE, Hoque DM, Akter T, Rahman M, Hoque ME, Begum K, Chowdhury EK, Khan R, Blum LS, Ahmed S, Hossain MA, Siddik A, Begum N, Sadeq-ur Rahman Q, Haque TM, Billah SM, Islam M, Rumi RA, Law E, Al-Helal ZA, Baqui AH, Schellenberg J, Adam T, Moulton LH, Habicht JP, Scherpbier RW, Victora CG, Bryce J, Black RE. Effect of the Integrated Management of Childhood Illness strategy on childhood mortality and nutrition in a rural area in Bangladesh: a cluster randomised trial. Lancet. 2009 Aug 1;374(9687):393-403. doi: 10.1016/S0140-6736(09)60828-X. PubMed PMID: 19647607.

19 Tilahun H, Fekadu B, Abdisa H, Canavan M, Linnander E, Bradley EH, Berman P. Ethiopia's health extension workers use of work time on duty: time and motion study. Health Policy Plan. 2017 Apr 1;32(3):320-328. doi: 10.1093/heapol/czw129. PubMed PMID: 27658649.

20 Hayes RJ, Bennett S. Simple sample size calculation for cluster-randomized trials. Int J Epidemiol. 1999 Apr;28(2):319-26.

21 The DHS program. Demographic and Health surveys. https://dhsprogram.com/.

22 Thayaparan, A. a. (2013). The Patient Satisfaction Questionnaire Short Form (PSQ-18) as an adaptable, reliable, and validated tool for use in various settings. Med Educ Online, 18.

23 The Health Foundation. (2013, June). Measuring patient experience (no.18). Retrieved Nov 6, 2018, from www.health.org.uk: https://www.health.org.uk/publication/measuring-patient-experience.

For peer review only

24 Grogan, S. C. (2000). Validation of a questionnaire measuring patient satisfaction with general practitioner services. Quality in Health Care, 9, 210-215.

25 LaVela, S. L. (2014). Evaluation and measurement of patient experience. Patient Experience Journal, 1(1), Article 5.

26 Alpern, R. C. (2013, November). Development of a Brief Instrument for Assessing Healthcare Employee Satisfaction in a Low-Income Setting. PLOS ONE, 8(11), e79053.

27 Chang, E. C. (2017). Measuring job satisfaction among healthcare staff in the United States: a confirmatory factor analysis of the Satisfaction of Employees in health Care (SEHC) survey. International Journal for Quality in health Care, 29(2), 262-268.

28 Open Data Kit. https://opendatakit.org/. Accessed 5th February 2021.

29 Pocock S, et al. Subgroup analysis, covariate adjustment and baseline comparisons in clinical trial reporting: current practice and problems. Stat Med. 2002;21:2917-30.

30 The CONSORT Group. CONSORT Statement [Internet]; item 15.

31 R Core Team. R: A language and environment for statistical computing. R Foundation for Statistical Computing, Vienna, Austria. URL https://www.R-project.org/.

32 Torgerson David J. Contamination in trials: is cluster randomisation the answer? BMJ 2001; 322:355.

33 Benjamin-Chung J, Arnold BF, Berger D, Luby SP, Miguel E, Colford JM Jr, Hubbard AE. Spillover effects in epidemiology: parameters, study designs and methodological considerations. Int J Epidemiol. 2018 Feb 1;47(1):332-347. doi: 10.1093/ije/dyx201. PubMed PMID: 29106568; PubMed Central PMCID: PMC5837695.

34 Leon N, Balakrishna Y, Hohlfeld A, Odendaal WA, Schmidt BM, Zweigenthal V, Anstey Watkins J, Daniels K. Routine Health Information System (RHIS) improvements for strengthened health system management. Cochrane Database Syst Rev. 2020 Aug 13;8:CD012012. doi: 10.1002/14651858.CD012012.pub2. PMID: 32803893.

35 Allanson ER, Tunçalp Ö, Vogel JP, Khan DN, Oladapo OT, Long Q, Gülmezoglu AM. Implementation of effective practices in health facilities: a systematic review of cluster randomised trials. BMJ Glob Health. 2017 Jul 20;2(2):e000266. doi: 10.1136/bmjgh-2016-000266. PMID: 29081997; PMCID: PMC5656132.

For peer review only

36 Brown DW, Bosch-Capblanch X, Shimp L. Where Do We Go From Here? Defining an Agenda for Home-Based Records Research and Action Considering the 2018 WHO Guidelines. Glob Health Sci Pract. 2019 Mar 29;7(1):6-11. doi: 10.9745/GHSP-D-18-00431. PMID: 30877139; PMCID: PMC6538131.

37 Joos O, Silva R, Amouzou A, Moulton LH, Perin J, Bryce J, et al. Evaluation of a mhealth data quality intervention to improve documentation of pregnancy outcomes by health surveillance assistants in Malawi: a cluster randomized trial. PloS One 2016;11(1):e0145238. PMID: 26731401. DOI: https://doi.org/10.1371/journal.pone.0145238

38 Langlois EV, Ranson MK, Bärnighausen T, Bosch-Capblanch X, Daniels K, El-Jardali F, Ghaffar A, Grimshaw J, Haines A, Lavis JN, Lewin S, Meng Q, Oliver S, Pantoja T, Straus S, Shemilt I, Tovey D, Tugwell P, Waddington H, Wilson M, Yuan B, Røttingen JA. Advancing the field of health systems research synthesis. Syst Rev. 2015 Jul 10;4:90. doi: 10.1186/s13643-015-0080-9. PMID: 26159806; PMCID: PMC4498528.

39 Bosch-Capblanch X, Lavis JN, Lewin S, Atun R, Røttingen JA, Dröschel D, Beck L, Abalos E, El-Jardali F, Gilson L, Oliver S, Wyss K, Tugwell P, Kulier R, Pang T, Haines A. Guidance for evidence-informed policies about health systems: rationale for and challenges of guidance development. PLoS Med. 2012;9(3):e1001185. doi: 10.1371/journal.pmed.1001185. Epub 2012 Mar 6. PMID: 22412356; PMCID: PMC3295823.

40 WHO. WHO recommendations on home-based records for maternal, newborn and child health. WHO 2018.

41 WHO. Recommendations on digital interventions for health system strengthening. WHO guideline. WHO 2019.

For peer review only

Tables and Figures

Table 1. Outcomes and parameters used to estimate the sample sizes.

	Outcome name	Subjects	Definition	Baseline estimate	Expected change	Comments
1	Vaccination adherence	Children under-1 in (sample of households in catchment areas)	Number of vaccines given in the previous calendar year over the number of vaccine due in the same period	75 given per 100 due	Increase of 10 per 100	Vaccines are clustered within children, and children within HFs
2	Data concordance	Recording tools in health facilities (samples of records)	Number of health care events (e.g. vaccinations, antenatal care consultations) recounted in the previous calendar year versus the number of health care events reported in the same time period	7 recounted for each 10 reported [3]	Increase of 1 recounted	A single estimate can be obtained in each HF or by time periods (no clustering)
3	Diagnostic scope	Records of sick child consultations (samples of records)	Number of diagnosis in each sick child consultation during the previous calendar year	30% with more than 1 diagnosis	35% with more than 1 diagnosis	Individual consultations are clustered within HF
4	Treatment appropriateness	Records of sick child consultations (samples of records)	Number of treatments correctly prescribed in each sick child consultation during the previous calendar year	Half appropriate over all consultation s	Increase to 60%	Individual consultations are clustered within HF (one treatment per child)
5	Health workforce satisfaction	Health workers (all health workers form include health facilities)	Degree (score) of satisfaction across all health facilities in each arm, with the intervention	50% satisfied	75% satisfied	Maybe two or three health workers can be approached in each health facility

Figure 1. PHISICC research programme structure, processes, deliverables and flow of evidence.

Footnote to Figure 1. WS: work stream. Timelines are approximate.

For peer review only

Figure 2. CONSORT diagram: trial flow chart.

Separate file.

For peer review only

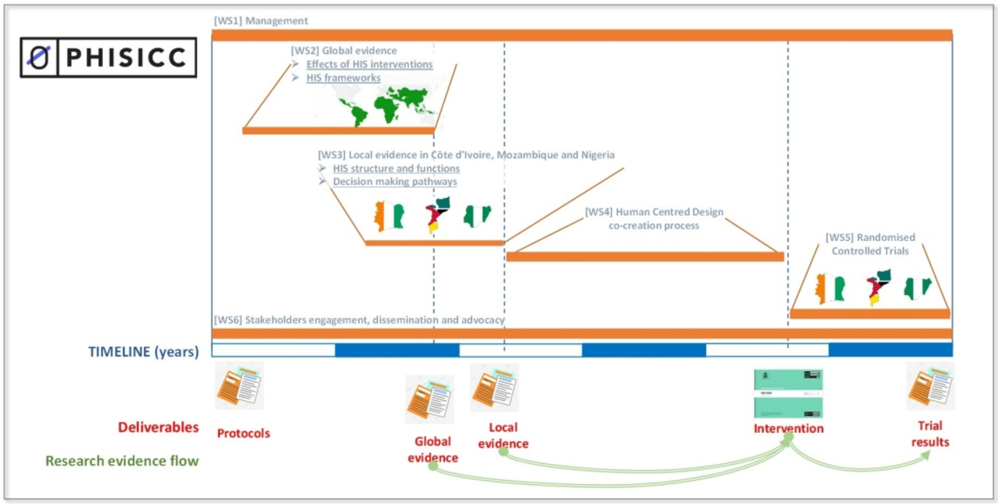

Figure 1. PHISICC research programme structure, processes, deliverables and flow of evidence.

312x157mm (150 x 150 DPI)

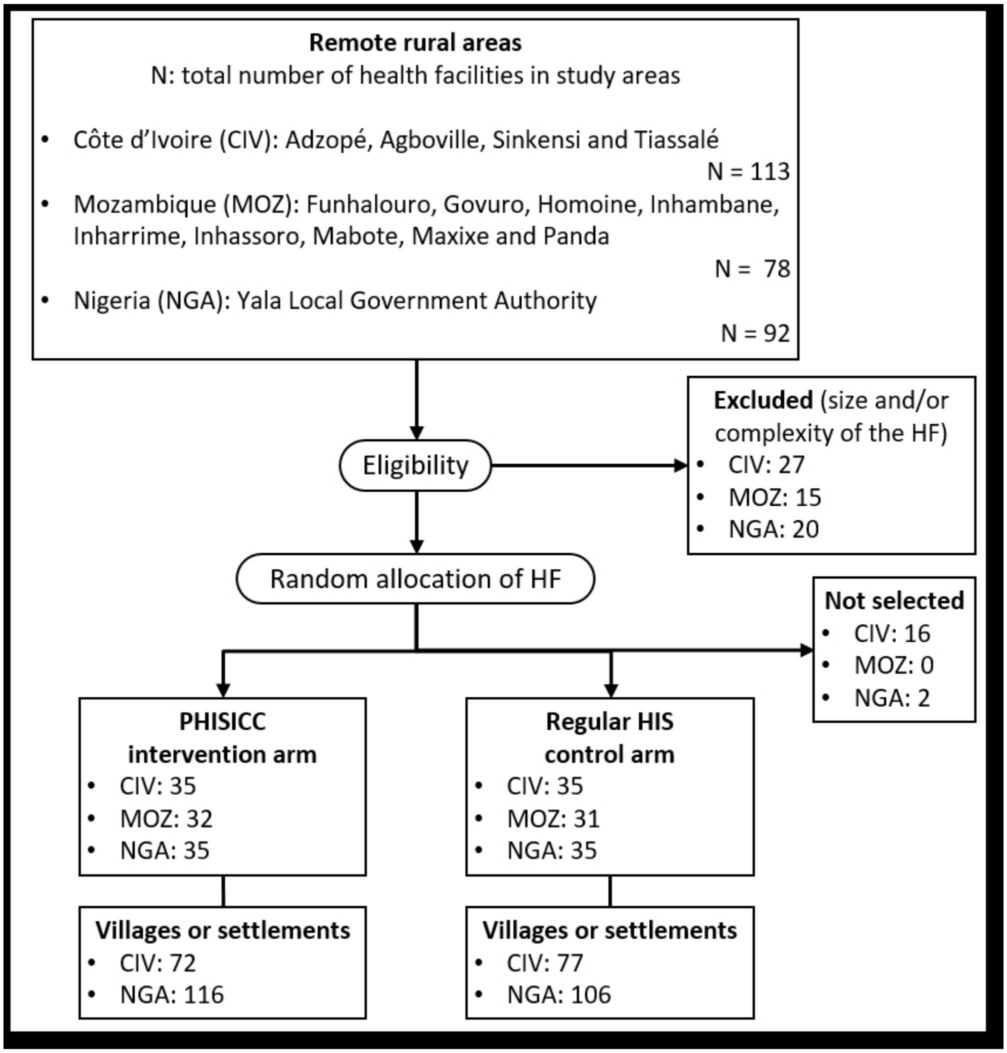

Figure 12. CONSORT diagram: trial flow chart.

144x150mm (150 x 150 DPI)

```
#
# clustersampleSize_proportions_baseline&endline.r
# get power of cluster randomised trial for binary outcomes (baseline and
endline surveys)
# 2 groups (control & intervention)
# clustered within HF


rm(list=ls())

# if the package lme4 is not already installed (needed for regression with
random effects)
# install.packages(lme4)
require(lme4)
#install.packages("reshape")
library(reshape)


# INPUTS
numGroups<-2
numHFPerGroup<-35
numTrialsToSimulate<-100
# numTrialsToSimulate: use 10 to test that the script runs, use 100 or 1000 for
precise estimate of power



# choose input set and remove #s to run

# inputs for 'treatments with appropriate diagnosis'
 pInterv<-0.60
 pControl<-0.50
 sdHFcluster<-0.55
# for k=0.1, 0.20; for k=0.25, 0.55
 numObsPerHF<-25

# inputs for vaccination adherence
# proportions in interventions and control groups
# pInterv<-0.8
# pControl<-0.75
# sdHFcluster<-2.63
# numObsPerHF<-30


# inputs for 'more than one diagnosis'
# pInterv<-0.35
# pControl<-0.30
# sdHFcluster<-0.39
# for k=0.1, 0.16; for k=0.25, 0.39
# numObsPerHF<-60


# NB getsd is a function at the bottom of the script to turn k into sdHFcluster
(sdHFcluster is on the logit scale)
```

For peer review only

```
# --- simulation ----

  # SET UP DATA STRUCTURE (intervention, HF)
  totNumHF <- numHFPerGroup*numGroups
  HFList<-seq(1:totNumHF)
  interv<- rep(c(0,1),each=(totNumHF/2) )
  intervEffect<-rep( c(0,(log(pInterv/(1-pInterv)) -
log(pControl/(1-pControl))) ), each=(totNumHF/2) )

  xtemp<-cbind(interv,HFList,intervEffect)

  # SET UP STORE FOR PVALUES AND PRECISION
  storeResults<-array(-9,dim=c(numTrialsToSimulate,3))
  colnames(storeResults)<-c("pvalue","coeff","stderr")


  # LOOP THROUGH THE SIMULATIONS

  for (i in 1:numTrialsToSimulate) {

    # simulate the HF cluster effects
      HFEffect<-rnorm(totNumHF,mean=0,sd=sdHFcluster)
      xtemp2a<-cbind(xtemp, HFEffect)
      xtemp2a<-data.frame(xtemp2a)

      # get expected proportions (pre and post)
      xtemp2a$expectedprelogodds<-log(pControl/(1-pControl)) + xtemp2a$HFEffect

      xtemp2a$expectedpostlogodds<-log(pControl/(1-pControl)) +
xtemp2a$intervEffect + xtemp2a$HFEffect

xtemp2a$expectedpre<-exp(xtemp2a$expectedprelogodds)/(1+exp(xtemp2a$expectedpre
logodds))

xtemp2a$expectedpost<-exp(xtemp2a$expectedpostlogodds)/(1+exp(xtemp2a$expectedp
ostlogodds))

      # expand by the number of observations per HF
      xtemp2b<-untable(xtemp2a, num=numObsPerHF)
      numObs<-dim(xtemp2b)[1]

    # simulate individual observations from cluster mean rates
      simObsPost<-rep(0,numObs)
      simObsPre<-rep(0,numObs)
      for (j in 1:numObs) {
         simObsPost[j]<-rbinom(n=1, size=1,prob=xtemp2b$expectedpost[j])
         simObsPre[j]<-rbinom(n=1, size=1,prob=xtemp2b$expectedpre[j])
      }
      # drop variables not needed further
      xtemp2b$expectedpostlogodds<-NULL; xtemp2b$expectedprelogodds<-NULL
```

For peer review only

```
    # stack pre and post observations
      # get post
      xtemp3<-cbind(xtemp2b,simObsPost)
      xtemp3<-data.frame(xtemp3)
      xtemp3$simObs<-xtemp3$simObsPost
      xtemp3$simObsPost<-NULL
      xtemp3$post<-1
      # get pre
      xtemp4<-cbind(xtemp2b,simObsPre)
      xtemp4<-data.frame(xtemp4)
      xtemp4$simObs<-xtemp4$simObsPre
      xtemp4$simObsPre<-NULL
      xtemp4$post<-0
      xtemp4$interv<-0
      xtemp5<-rbind(xtemp3,xtemp4)


     # carry out analysis for individual trial
      m <- glmer(simObs ~ as.factor(interv) +  post + (1 | HFList),
data<-xtemp5, family=binomial)

     # store result of individual trial in storeResults (p-value, coefficient
and std error)
          out1<-summary(m)$coefficients
          storeResults[i,2]<-out1[2,1]
          storeResults[i,3]<-out1[2,2]
          storeResults[i,1]<-out1[2,4]

   print(i)

  }  # End of loop

  # calculate power
  pvalue<-storeResults[,1]
  power<-length(pvalue[pvalue<0.05])/length(pvalue)

  cat("power ", power, "\n")



# -------- run to here -----







# ------
# getsd: function to estimate between-cluster variation from k (Hayes and
Bennet sd/mean) and input base proportion (base0p)
```

For peer review only

```
getsd<-function(base0p,k){
     sdcluster<-k*base0p
     clusterEffect<-rnorm(1000,mean=0,sd=sdcluster)
     expectedp<-base0p + clusterEffect
     expectedp[expectedp>1]<-0.9999
     expectedp[expectedp<0]<-0.0001
     logitexpectedp<-log((expectedp)/(1-expectedp))
     sdlog<-sd(logitexpectedp)
     cat("estimated sdlog ", sdlog, "\n")
}

# example
getsd(0.30,0.25)

getsd(0.50, 0.25)
```

For peer review only

```
#
# clusterSampleSize_concordance.r
# get power of cluster randomised trial
# ratios (outcome is continuous)
# fixed to 2 groups
# records and reports clustered within HF
#


# if the package lme4 is not already installed (needed for regression with
random effects)
# install.packages(lme4)
# install.packages(lmerTest)
require(lme4)
require(lmerTest)


# EXAMPLE INPUTS
numGroups<-2
numHFPerGroup<-35
numReportedPerHF<-100
# assuming equal numbers of vaccinations per HF
numTrialsToSimulate<-100
# 100 or 1000 needed for precision of the power estimate, use 10 for test runs

ratioControl<-0.7
ratioInterv<-0.8
sdHFcluster<-0.25*0.8
# sdHFcluster is on the log scale, calculated using k=0.25



# --- run simulation from here ----

  # SET UP DATA STRUCTURE (intervention, HF)
  totNumHF<-numGroups*numHFPerGroup
  HFList<-rep(seq(1:(numHFPerGroup*numGroups)),each=1)
  interv<-c( rep(c(0,1),each=(totNumHF/2)))
  intervEffect<-rep( c(0,(ratioInterv - ratioControl )), each=(totNumHF/2) )
  xtemp<-cbind(interv,HFList,intervEffect)

  # SET UP STORE FOR PVALUES AND PRECISION
  storeResults<-array(-9,dim=c(numTrialsToSimulate,3))
  colnames(storeResults)<-c("pvalue","coeff","stderr")


  # LOOP THROUGH THE SIMULATIONS

  for (i in 1:numTrialsToSimulate) {

    # simulate the HF cluster effects

      HFEffect<-rnorm(numHFPerGroup*numGroups,mean=0,sd=sdHFcluster)
      xtemp2<-cbind(xtemp, HFEffect)
```

For peer review only

```
      # get expected ratios (pre and post)
       expectedpreratio<-ratioControl + HFEffect
       expectedpostratio<-ratioControl + intervEffect + HFEffect
       expectedpreratio[expectedpreratio<0.0001]<-0.0001
       expectedpostratio[expectedpostratio<0.0001]<-0.0001

      # simulate individual observations as poisson rate of number reported per 
1 recorded
       simObsPost<-rep(0,length(expectedpostratio))
       simObsPre<-rep(0,length(expectedpreratio))
       for (j in 1:length(expectedpostratio)) {
           simObsPost[j]<-rpois(n=1,expectedpostratio[j]*numReportedPerHF)
           simObsPre[j]<-rpois(n=1,expectedpreratio[j]*numReportedPerHF)
       }


     # stack pre and post observations
       # post
       xtemp3<-cbind(xtemp2,simObsPost)
       xtemp3<-data.frame(xtemp3)
       xtemp3$simObs<-xtemp3$simObsPost
       xtemp3$simObsPost<-NULL
       xtemp3$post<-1
       # pre
       xtemp4<-cbind(xtemp2,simObsPre)
       xtemp4<-data.frame(xtemp4)
       xtemp4$simObs<-xtemp4$simObsPre
       xtemp4$simObsPre<-NULL
       xtemp4$post<-0
       xtemp4$interv<-0
       # stack pre and post
       xtemp5<-rbind(xtemp3,xtemp4)
         xtemp5$distanceToOne<-abs(1-(xtemp5$simObs/numReportedPerHF))


       # carry out analysis for individual trial
       m <- lmer(distanceToOne ~ as.factor(interv) +  post + (1|HFList), 
data=xtemp5)

       # store result of individual trial in storeResults (p-value, coefficient 
and std error)
        out1<-summary(m)$coefficients
        # estimate
        storeResults[i,2]<-out1[2,1]
        # se
        storeResults[i,3]<-out1[2,2]
        # p-value
        storeResults[i,1]<-out1[2,5]

      print(i)

   }  # End of loop
```

For peer review only

```
  # calculate power
  pvalue<-storeResults[,1]
  power<-length(pvalue[pvalue<0.05])/length(pvalue)

  cat("power ", power, "\n")


# -------------
```

For peer review only

Table 1: CONSORT 2010 checklist of information to include when reporting a cluster randomised trial

Section/Topic	Item No	Standard Checklist item	Extension for cluster designs	Page No *
**Title and abstract**				
	1a	Identification as a randomised trial in the title	Identification as a cluster randomised trial in the title	1
	1b	Structured summary of trial design, methods, results, and conclusions	See table 2	5
**Introduction**				
**Background and objectives**	2a	Scientific background and explanation of rationale	Rationale for using a cluster design	4
	2b	Specific objectives or hypotheses	Whether objectives pertain to the cluster level, the individual participant level or both	5
**Methods**				
**Trial design**	3a	Description of trial design (such as parallel, factorial) including allocation ratio	Definition of cluster and description of how the design features apply to the clusters	6
	3b	Important changes to methods after trial commencement (such as eligibility criteria), with reasons		Not applicable
**Participants**	4a	Eligibility criteria for participants	Eligibility criteria for clusters	6
	4b	Settings and locations where the data were collected		6
**Interventions**	5	The interventions for each group with sufficient details to allow replication, including how and when they were actually administered	Whether interventions pertain to the cluster level, the individual participant level or both	8
**Outcomes**	6a	Completely defined pre-specified primary and secondary outcome measures, including how and when they were assessed	Whether outcome measures pertain to the cluster level, the individual participant level or both	10 and Table 1
	6b	Any changes to trial outcomes after the trial commenced, with reasons		Not applicable
**Sample size**	7a	How sample size was determined	Method of calculation, number of clusters(s) (and whether equal or unequal cluster sizes are assumed), cluster size, a coefficient of intracluster correlation (ICC or *k*), and an indication of its uncertainty	12
	7b	When applicable, explanation of any interim analyses and stopping guidelines		Not yet applicable

Section/Topic	Item No	Standard Checklist item	Extension for cluster designs	Page No *
**Randomisation:**				
**Sequence generation**	8a	Method used to generate the random allocation sequence		7, 8
	8b	Type of randomisation; details of any restriction (such as blocking and block size)	Details of stratification or matching if used	Not applicblae
**Allocation concealment mechanism**	9	Mechanism used to implement the random allocation sequence (such as sequentially numbered containers), describing any steps taken to conceal the sequence until interventions were assigned	Specification that allocation was based on clusters rather than individuals and whether allocation concealment (if any) was at the cluster level, the individual participant level or both	7
**Implementation**	10	Who generated the random allocation sequence, who enrolled participants, and who assigned participants to interventions	Replace by 10a, 10b and 10c	7
	10a		Who generated the random allocation sequence, who enrolled clusters, and who assigned clusters to interventions	7
	10b		Mechanism by which individual participants were included in clusters for the purposes of the trial (such as complete enumeration, random sampling)	10
	10c		From whom consent was sought (representatives of the cluster, or individual cluster members, or both), and whether consent was sought before or after randomisation	8
**Blinding**	11a	If done, who was blinded after assignment to interventions (for example, participants, care providers, those assessing outcomes) and how		8
	11b	If relevant, description of the similarity of interventions		Not applicable
**Statistical methods**	12a	Statistical methods used to compare groups for primary and secondary outcomes	How clustering was taken into account	15
	12b	Methods for additional analyses, such as subgroup analyses and adjusted analyses		15

For peer review only

Section/Topic	Item No	Standard Checklist item	Extension for cluster designs	Page No *
**Results**				Not yet applicable (protocol manuscript)
**Discussion**				
**Limitations**	20	Trial limitations, addressing sources of potential bias, imprecision, and, if relevant, multiplicity of analyses		18
**Generalisability**	21	Generalisability (external validity, applicability) of the trial findings	Generalisability to clusters and/or individual participants (as relevant)	18,19
**Interpretation**	22	Interpretation consistent with results, balancing benefits and harms, and considering other relevant evidence		Not yet applicable
**Other information**				
**Registration**	23	Registration number and name of trial registry		1
**Protocol**	24	Where the full trial protocol can be accessed, if available		1
**Funding**	25	Sources of funding and other support (such as supply of drugs), role of funders		20

** Page numbers: as seen in the document "draft_Proof_hi.pdf" (which has 34 pages)*

For peer review only

CONSORT 2010 checklist of information to include when reporting a cluster randomised trial

Section/Topic	Item No	Standard Checklist item	Extension for cluster designs	Page No *
**Title and abstract**				
	1a	Identification as a randomised trial in the title	Identification as a cluster randomised trial in the title	1
	1b	Structured summary of trial design, methods, results, and conclusions	See table 2	5
**Introduction**				
**Background and objectives**	2a	Scientific background and explanation of rationale	Rationale for using a cluster design	4
	2b	Specific objectives or hypotheses	Whether objectives pertain to the cluster level, the individual participant level or both	5
**Methods**				
**Trial design**	3a	Description of trial design (such as parallel, factorial) including allocation ratio	Definition of cluster and description of how the design features apply to the clusters	6
	3b	Important changes to methods after trial commencement (such as eligibility criteria), with reasons		Not applicable
**Participants**	4a	Eligibility criteria for participants	Eligibility criteria for clusters	6
	4b	Settings and locations where the data were collected		6
**Interventions**	5	The interventions for each group with sufficient details to allow replication, including how and when they were actually administered	Whether interventions pertain to the cluster level, the individual participant level or both	8
**Outcomes**	6a	Completely defined pre-specified primary and secondary outcome measures, including how and when they were assessed	Whether outcome measures pertain to the cluster level, the individual participant level or both	10 and Table 1
	6b	Any changes to trial outcomes after the trial commenced, with reasons		Not applicable
**Sample size**	7a	How sample size was determined	Method of calculation, number of clusters(s) (and whether equal or unequal cluster sizes are assumed), cluster size, a coefficient of intracluster correlation (ICC or *k*), and an indication of its uncertainty	12
	7b	When applicable, explanation of any interim analyses and stopping guidelines		Not yet applicable

Section/Topic	Item No	Standard Checklist item	Extension for cluster designs	Page No *
**Randomisation:**				
**Sequence generation**	8a	Method used to generate the random allocation sequence		7, 8
	8b	Type of randomisation; details of any restriction (such as blocking and block size)	Details of stratification or matching if used	Not applicable
**Allocation concealment mechanism**	9	Mechanism used to implement the random allocation sequence (such as sequentially numbered containers), describing any steps taken to conceal the sequence until interventions were assigned	Specification that allocation was based on clusters rather than individuals and whether allocation concealment (if any) was at the cluster level, the individual participant level or both	7
**Implementation**	10	Who generated the random allocation sequence, who enrolled participants, and who assigned participants to interventions	Replace by 10a, 10b and 10c	7
	10a		Who generated the random allocation sequence, who enrolled clusters, and who assigned clusters to interventions	7
	10b		Mechanism by which individual participants were included in clusters for the purposes of the trial (such as complete enumeration, random sampling)	10
	10c		From whom consent was sought (representatives of the cluster, or individual cluster members, or both), and whether consent was sought before or after randomisation	8
**Blinding**	11a	If done, who was blinded after assignment to interventions (for example, participants, care providers, those assessing outcomes) and how		8
	11b	If relevant, description of the similarity of interventions		Not applicable
**Statistical methods**	12a	Statistical methods used to compare groups for primary and secondary outcomes	How clustering was taken into account	15
	12b	Methods for additional analyses, such as subgroup analyses and adjusted analyses		15

For peer review only

Section/Topic	Item No	Standard Checklist item	Extension for cluster designs	Page No *
**Results**				Not yet applicable (protocol manuscript)
**Discussion**				
**Limitations**	20	Trial limitations, addressing sources of potential bias, imprecision, and, if relevant, multiplicity of analyses		18
**Generalisability**	21	Generalisability (external validity, applicability) of the trial findings	Generalisability to clusters and/or individual participants (as relevant)	18,19
**Interpretation**	22	Interpretation consistent with results, balancing benefits and harms, and considering other relevant evidence		Not yet applicable
**Other information**				
**Registration**	23	Registration number and name of trial registry		1
**Protocol**	24	Where the full trial protocol can be accessed, if available		1
**Funding**	25	Sources of funding and other support (such as supply of drugs), role of funders		20

For peer review only

BMJ Open

Does an innovative paper-based health information system (PHISICC) improve data quality and use in primary health care? Protocol of a multi-country, cluster randomised controlled trial in Sub-Saharan African rural settings.

Journal:	*BMJ Open*
Manuscript ID	bmjopen-2021-051823.R2
Article Type:	Protocol
Date Submitted by the Author:	02-Jul-2021
Complete List of Authors:	Bosch-Capblanch, Xavier; Swiss Tropical and Public Health Institute; University of Basel<br>Oyo-Ita, Angela; University of Calabar, Department of Community Medicine<br>Muloliwa, Artur; Lurio University Faculty of Health Sciences<br>Yapi, Richard; Centre Suisse de Recherches Scientifiques en Côte d'Ivoire<br>Auer, Christian ; Swiss Tropical and Public Health Institute; University of Basel<br>Samba, Mamadou; Ministère de la Santé et de l'Hygiène Publique<br>Gajewski, Suzanne; Swiss Tropical and Public Health Institute; University of Basel<br>Ross, Amanda; Swiss Tropical and Public Health Institute; University of Basel<br>Krause, L Kendall; Bill & Melinda Gates Foundation<br>Ekpenyong, Nnette; University of Calabar Teaching Hospital, Department of Community Medicine<br>Nwankwo, Ogonna; University of Calabar, Department of Community Medicine<br>Njepuome, Anthonia; Swiss Tropical and Public Health Institute<br>Lee, Sofia; Swiss Tropical and Public Health Institute<br>Sacarlal, Jahit ; Eduardo Mondlane University, Faculty of Medicine<br>Madede, Tavares; Universidade Eduardo Mondlane<br>Berté, Salimata; Centre Suisse de Recherches Scientifiques en Côte d'Ivoire<br>Matsinhe, Graça; Ministério da Saúde<br>Garba, Abdullahi; National Primary Healthcare Development Agency<br>Brown, David; BCGI LLC / pivot-23.5°
**Primary Subject Heading**:	Health services research
Secondary Subject Heading:	Global health, Health policy, Research methods
Keywords:	Human resource management < HEALTH SERVICES ADMINISTRATION & MANAGEMENT, Organisation of health services < HEALTH SERVICES ADMINISTRATION & MANAGEMENT, Protocols & guidelines < HEALTH SERVICES ADMINISTRATION & MANAGEMENT, Quality in health care <

	HEALTH SERVICES ADMINISTRATION & MANAGEMENT, STATISTICS & RESEARCH METHODS, PRIMARY CARE

SCHOLARONE™ Manuscripts

I, the Submitting Author has the right to grant and does grant on behalf of all authors of the Work (as defined in the below author licence), an exclusive licence and/or a non-exclusive licence for contributions from authors who are: i) UK Crown employees; ii) where BMJ has agreed a CC-BY licence shall apply, and/or iii) in accordance with the terms applicable for US Federal Government officers or employees acting as part of their official duties; on a worldwide, perpetual, irrevocable, royalty-free basis to BMJ Publishing Group Ltd ("BMJ") its licensees and where the relevant Journal is co-owned by BMJ to the co-owners of the Journal, to publish the Work in this journal and any other BMJ products and to exploit all rights, as set out in our licence.

The Submitting Author accepts and understands that any supply made under these terms is made by BMJ to the Submitting Author unless you are acting as an employee on behalf of your employer or a postgraduate student of an affiliated institution which is paying any applicable article publishing charge ("APC") for Open Access articles. Where the Submitting Author wishes to make the Work available on an Open Access basis (and intends to pay the relevant APC), the terms of reuse of such Open Access shall be governed by a Creative Commons licence – details of these licences and which Creative Commons licence will apply to this Work are set out in our licence referred to above.

Other than as permitted in any relevant BMJ Author's Self Archiving Policies, I confirm this Work has not been accepted for publication elsewhere, is not being considered for publication elsewhere and does not duplicate material already published. I confirm all authors consent to publication of this Work and authorise the granting of this licence.

For peer review only

Title

Does an innovative paper-based health information system (PHISICC) improve data quality and use in primary health care? Protocol of a multi-country, cluster randomised controlled trial in Sub-Saharan African rural settings.

Authors

- Xavier Bosch-Capblanch. Corresponding author. Swiss Tropical and Public Health Institute. Basel, Switzerland. And University of Basel, Basel, Switzerland. x.bosch@unibas.ch
- Angela Oyo-Ita. Department of Community Medicine, University of Calabar, Calabar, Nigeria. oyo_ita@yahoo.com
- Artur Manuel Muloliwa. Faculty of Health Sciences, Lúrio University, Nampula, Mozambique. muloliwa@yahoo.com.br
- Richard B Yapi. Centre Suisse de Recherches Scientifiques en Côte d'Ivoire, Abidjan, Côte d'Ivoire. And Centre d'Entomologie Médicale et Vétérinaire, Université Alassane Ouattara, Bouaké, Côte d'Ivoire. richard.yapi@csrs.ci
- Christian Auer. Swiss Tropical and Public Health Institute. Basel, Switzerland. And University of Basel, Basel, Switzerland. christian.auer@swisstph.ch
- Mamadou Samba. Ministère de la Santé et de l'Hygiène Publique, Abidjan, Côte d'Ivoire. And Université Félix Houphouet Boigny, Abidjan, Côte d'Ivoire. samba.mamadou@gmail.com
- Suzanne Gajewski. Swiss Tropical and Public Health Institute. Basel, Switzerland. And University of Basel, Basel, Switzerland. suzanne.gajewski@swisstph.ch
- Amanda Ross. Swiss Tropical and Public Health Institute. Basel, Switzerland. And University of Basel, Basel, Switzerland. amanda.ross@swisstph.ch
- L Kendall Krause. Bill & Melinda Gates Foundation, Seattle, USA. Kendall.Krause@gatesfoundation.org
- Nnette Ekpenyong. Department of Community Medicine, University of Calabar Teaching Hospital, Calabar, Nigeria. And Department of Community Medicine University of Calabar, Calabar, Nigeria. nnekon2015@gmail.com
- Ogonna Nwankwo. Swiss Tropical and Public Health Institute, Basel, Switzerland. And University of Basel, Basel, Switzerland. And Health Policy, Management and Economics Firm, Department of Community. Medicine, University of Calabar, Calabar, Nigeria. ogonna.nwankwo@swisstph.ch
- Anthonia Ngozi Njepuome. Swiss Tropical and Public Health Institute, Abuja, Nigeria. ngonjep@yahoo.com
- Sofia Mandjate Lee. Swiss Tropical and Public Health Institute; Manhiça, Mozambique. sofia_mandjate@yahoo.com.br
- Jahit Sacarlal. Faculty of Medicine, Eduardo Mondlane University, Maputo, Mozambique. jahityash2002@gmail.com
- Tavares Madede. Department of Community Health, Faculty of Medicine, Eduardo Mondlane University, Maputo, Mozambique. tmadede@gmail.com
- Salimata Berté. Centre Suisse de Recherches Scientifiques en Côte d'Ivoire, Abidjan, Côte d'Ivoire. sali.berte@csrs.ci
- Graça Matsinhe. Expanded Program on Immunization, Ministry of Health, Maputo, Mozambique. gmatsinhe@gmail.com.
- Abdullahi Bulama Garba. Director Planning, Research and Statistics, National Primary Health Care Development Agency, Abuja, Nigeria. ab.garba@nphcda.gov.ng.
- David W Brown. Managing Principal, BCGI LLC / pivot-23.5°, North Carolina, USA. david.brown@pivot235.org

For peer review only

Abstract

Introduction

Frontline health workers in remote health facilities are the first contact of the formal health sector and are confronted with life-saving decisions. Health information systems (HIS) support the collection and use of data. However, HIS focus on reporting and are unfit to support decisions. Since data tools are paper-based in most primary health care settings, we have produced an innovative paper-based HIS (PHISICC) using a Human Centred Design approach. We are carrying out a cluster-randomised controlled trial in three African countries to assess the effects of PHISICC compared with the current systems.

Methods and analysis

Study areas are in rural zones of Côte d'Ivoire, Mozambique and Nigeria. Seventy health facilities in each country have been randomly allocated to using PHISICC tools or to continuing to use the regular HIS tools. We have randomly selected households in the catchment areas of each health facility to collect outcomes' data. The baseline survey has been carried out in two of the three countries, the end-line survey is planned for mid-2021. Primary outcomes include data quality and use and coverage of health services and health workers satisfaction; secondary outcomes are additional data quality and use parameters, childhood mortality and additional health workers and clients experience with the system. Just prior to the implementation of the trial we had to relocate the studies in Mozambique and Côte d'Ivoire due to unforeseen logistical issues. The effects of the intervention will be estimated using regression models and accounting for clustering using random effects.

Ethics and dissemination

Ethics committees in Côte d'Ivoire, Mozambique and Nigeria approved the trials. We plan to disseminate our findings, data and research materials among researchers and policy makers. We aim at having our findings included in systematic reviews on health systems interventions and future guidance development on the matter.

Registration

Pan African Clinical Trials Registry - PACTR201904664660639. Registered 01/04/2019, https://pactr.samrc.ac.za/Search.aspx.

Article summary

Strengths and limitations of the study

- These trials assess the effects of improving paper-based health information systems, which are greatly used particularly in remote, rural areas but which are neglected in research.
- The paper-based interventions have been developed using a Human Centred Design approach, with frontline health workers and designers driving the co-creation process.
- Despite the complexity of health systems interventions, we have applied robust experimental methods, together with qualitative research, to assess and understand the effects of the paper-based intervention. Robust evidence on health systems is more likely to gain the credibility of policy-makers and to make it into systematic reviews, guidance development and policy and practice.
- Research targeting frontline health workers in remote, rural areas has to take place where they live and work, which poses serious obstacles in the organisation, management and monitoring of the trials.
- These obstacles, aggravated by the COVID-19 pandemic, have challenged the mobility of the research team, the availability of the intervention in one of the countries and the duration of the trials.

For peer review only

Introduction

Frontline health workers (HW) in remote, rural health facilities (HF) in many countries are the first contact with the formal health sector of the population and they are confronted with life-saving clinical and public health decisions on a daily basis. Decisions are made by exerting a balanced judgment on the information related to health care events, such as making the correct diagnoses or deciding on which vaccinations a child should receive on a given day. In order to properly handle this information, appropriate data support tools and processes are required, referred to as the health information system (HIS); or Routine HIS or Health Management Information System [1]. In reality, though, HIS are primarily designed to report aggregated health events to the higher tiers of the health systems rather than to inform decision-making at the point of care [2].

Increasing pressure by donors and governments to collect more and more data has aggravated the situation, through the proliferation of data support tools that have overloaded frontline health workers compromising their capacity to deliver good quality of care and to delivery good quality data [3], for higher level decision-making.

Promising 'quick fixes', such as the scale up of digital HIS, are taking a long time to implement and face enormous challenges related to infrastructure, equipment and services necessary to run them. Besides, research evidence on the effects of digital solutions remains patchy and inconsistent, even in high-income country settings, where complaints about computerisation of clinical care have been raised [4,5]. Hence, it is very likely that paper tools will remain a primary, if not unique, data support mechanism particularly in remote, rural HF in many countries.

PHISICC (Paper-based Health Information System in Comprehensive Care) is a multi-year, multi-country, mixed-methods research project that aims at producing and testing an innovative paper-based HIS to improve data quality and use, decision making and health outcomes, at Primary Health Care (PHC). It is being carried out in selected areas within Côte d'Ivoire, Mozambique and Nigeria. The project started in 2015, producing a systematic review on the effects of HIS interventions [6,7]

and a framework synthesis on how HIS are understood in the literature in order to learn from past experiences in HIS developments. This global evidence was coupled with studies to characterise the existing HIS in the three countries, to understand how health workers interact with the HIS and to identify entry points for HIS design improvements. With these bodies the research team was well equipped to engage into a Human Centred Design (HCD) co-creative process with frontline HW to design an innovative HIS (PHISICC). See Figure 1 for an illustration of the structure, processes and evidence flow within PHISICC.

The impact of the PHISICC HIS on data quality and use, quality of health care and HW perceptions is being assessed concurrently in rural areas in the three countries. We describe the design of the trial here, consistent with CONSORT reporting guidelines [8] and the extension for cluster randomised controlled trials (CRCT) [9]; see Additional file 1.

Methods

Aim

The aim of the trial is to address the research question: what are the effects of an innovative paper-based HIS (PHISICC) on data use and quality, quality of health and HW perceptions compared with the current HIS, in rural PHC settings?

Patient and public involvement

There was no public or patient involvement in the design of the study or selection of study areas because the intervention being assessed in these trials target health care providers and decision-makers, rather than patients or the public in general. Population in the catchment area of selected health facilities, potentially using their services, were only approached in order to assess the studies outcomes.

On the other hand, we have involved health systems stakeholders and frontline health workers. Ministries of Health at several levels participated in the preparation of the research proposal

For peer review only

(personal consultations), in the characterisation of health information systems that preceded the trials (countries workshops), and throughout all project components (additional workshops, newsletters and personal communication). Frontline health workers in the three countries have co-created the intervention (i.e. paper based tools) through workshops, personal feedback and piloting under real live conditions. Some of them are part of the research team and co-authoring this manuscript.

Study design

The study is a CRCT in each of the three countries. In each setting, 70 health facilities are randomised to intervention or control (35 per arm). The intervention arm HF use the new PHISICC tools (substituting the usual HIS tools) and the control arm HF use the regular HIS tools. The trial is implemented in the real life contexts of HF carrying out their usual duties.

The trials started between the end of 2019 and beginning of 2020, depending on the country, when the intervention was installed and the baseline surveys carried out. Data collection will last until mid-2021.

Study areas

Ministries of Health (MOH) officials in several countries were contacted before submitting the proposal to the funding agency in order to explore the willingness to engage in a project focusing on paper-based tools. Officials in several countries rejected the offer on the grounds of upcoming digitalisation plans of the HIS in the country. We partnered with MOH that found the research relevant to their context in three countries.

In each country, the eligibility criteria of study areas were that they had to belong to the operational area of research partners; contain a large enough number of health facilities and their catchment population; include vulnerable population (e.g. with low vaccination coverage, high childhood mortality); and be comparatively neglected in terms of infrastructure and services. We excluded areas with concurrent research or other types of activities that could conflict with the CRCT (such as

For peer review only

the co-existence of another health-related study, massive developments in infrastructure or activities involving migration of the population, such as temporary work sites or changes in working sites) and areas with threats to safety or security that could jeopardise research activities.

The study areas are located in Adzopé, Agboville, Tiassalé and Sikensi districts (Côte d'Ivoire); in Funhalouro, Govuro, Homoine, Inhambane, Inharrime, Inhassoro, Mabote, Maxixe and Panda (Inhambane province, Mozambique); and in Yala Local Government Authority (Cross River State, Nigeria).

Eligibility of health facilities

The intervention is implemented at the HF level. The eligibility criteria of the HF were that they had to be located in the study areas, belong to the governmental health sector and their main activity should be the delivery of PHC services. HF were excluded if they had specialised clinical services, inpatients, physicians providing care or with plans for staff turn-over involving intervention and control HF.

A 'master list' of eligible health facilities was prepared based on information provided by the MOH across all study areas. We aimed at selecting 70 of the eligible HF in each country, using simple random techniques in R [10]. See in Figure 2 the selection and allocation trial flow chart.

Allocation and blinding

Allocation of the 70 HF per country into the intervention and control arms took place in a formal event, gathering research partners and MOH officials to offer transparency and promote study ownership by local and national authorities. Equally sized, folded pieces of paper with the names and codes of included HF written on them were introduced in an opaque receptacle where they were manually and blindly mixed. A second receptacle contained two equally sized pieces of paper, one with the word 'intervention' and another one with the word 'control'. A selected person in the meeting, not belonging to the research team, extracted one piece of paper at a time to reach half the number of included HF. Then, a paper was extracted from the second receptacle to assign those HF

For peer review only

to the intervention or control arms. The rest of the papers were extracted as well to verify completeness and no duplication of names, and those HF assigned to the other arm.

Once HF were selected, all villages or settlements for each health facility catchment area were listed and three in each catchment area were selected. In practice, we selected all villages because the numbers were below (in Côte d'Ivoire) or just above (in Nigeria) the needs. For each village, we used Google satellite maps to identify and geo-locate every visible roof. Where there were many houses per village (roughly, more than fifty or so), a researcher would mark four points in the map slightly beyond the northernmost, southernmost, easternmost and westernmost roofs seen and 30 random points were selected within that square. From the mapped points, 10 per village (with 10 more acting as reserve) were randomly selected and marked on another map used in the field for data collectors to approach households. Where technical problems impeded this approach in a given village, a field supervisor would rotate a bottle on the floor towards the centre of the village and would select at random 10 households in the direction pointed by the bottle, from the outer limit of the village till the centre [11].

Blinding is only feasible for the research team members carrying out the CRCT data collection and the analyses of the CRCT findings. The intervention (i.e. paper tools) are by design very different from the existing system and it is not possible to blind participants or principal researchers.

We already had the agreement of the MOH and selected HF compliant with the inclusion criteria were provided with the intervention shortly after completing the baseline data collection. Therefore, recruitment as such took place at the same time of the allocation of HF into intervention and control arms.

The intervention

The PHISICC paper-based intervention is a full set of paper-based tools to support decision-making by frontline HW. These are the only tools to be used by HW in the intervention arm. The PHISICC tools encompass the whole system (i.e. recording and reporting) and all clinical and public health care

For peer review only - http://bmjopen.bmj.com/site/about/guidelines.xhtml

areas and are characterised by a common visual language (e.g. spaces for digits and text), and standardised formats across health care areas. To support frontline health workers decision-making, the PHISICC tools incorporate specific places to explicitly record critical data items (e.g. respiratory rate in infants), graphic artefacts to distinguish severity degrees of signs or symptoms (i.e. small square for 'normality', diamond for 'attention' and bold diamond for 'critical severity'); space to document diagnoses and treatment decisions; and aides memoires in the first page of register books..

The PHISICC tools have been developed from May 2017 till June 2019, including production, using a Human Centred Design approach [12]. A strength of the Human Centred Design approach is its ability to unlock the user's perspective so that designers can build solutions that are fully reality-based and work well. Co-creation groups were formed in each country with researchers, staff from partner institutions and healthcare workers, led by a team of professional designers. Research team members supervised and coordinated exclusively the feedback on the contents of the tools, to ensure compliance with each country clinical guidelines. At the outset of the process, the design focused on three health care areas (i.e. antenatal care, vaccination and sick child) and slowly extended the new visual language to other health care areas. Initial workshops served to brainstorm on problems and potential design solutions, without any other rule than being comprehensive and not rejecting a single idea. Designers, then, formalised some of the most promising solutions and a first round of exchanges within the co-creation team was used to address misinterpretations or inconsistencies. There were two in-the-field testing rounds in Mozambique, two in Côte d'Ivoire and three in Nigeria and uncountable exchanges through teleconferences and email, in-between. The prototypes were considered final when no errors were detected, were compliant with data needs in each country and comments from the field could not be accommodated in the design concept or there was no consensus on minor or formal issues being raised.

The PHISICC tools have been produced in French for Côte d'Ivoire, in Portuguese for Mozambique and in English for Nigeria, which are the official languages used in the health systems in the three

For peer review only

countries. They include the official logo of the MOHs. Health care areas covered include: family planning, antenatal care, including tetanus toxoid vaccination, delivery, post-natal care, vaccination, sick child, adults outpatient consultation, tuberculosis diagnosis and treatment, and HIV. Referral forms were also designed.

The PHISICC tools have three sub-components: registers, tallies and reports. Registers are formed by seven DIN-A3 and one DIN-A4 (for referrals) book covering all health care areas except for tuberculosis treatment, for which DIN-A3 cards where used. Register books have 100, 200 or 400 pages depending on the country and health care area. They are used to record individual clients' data for each health care event, either of clinical or public health nature. Some register books have clinical notes at the very beginning, as 'aide memoires', and an example of a filled-in form, to assist HW when doubting how to proceed.

Tallies are DIN-A3 single sheets which contain a list of the indicators to be transferred to higher levels of the health system, with a series of small ovals, grouped in fives, to mark with tally sticks with a pen. In contrast to the current systems that have no tallies or only for vaccination, tallies were created for all health care areas. In the middle-right side of the tally, a column accommodates cells aligned with the ovals to insert the count for each indicator; and in the far right of the sheet there is a replica of the count column, separated with a perforated line, which is detached and sent, as part of the monthly report to the higher level in the health system.

While current HIS tools are consistently organised in tabular formats and books, where each clinical event is recorded in a row and each variable (e.g. age, gender, HIV status, diagnosis) in a column, PHISICC tools incorporated several innovations; in summary: a visual language to guide the clinical decisions of health workers based on severity (i.e. if it is recorded that a child has convulsions, a visual artefact indicates severity), more space for clinical data (e.g. vital signs), inclusion of all critical information to assess patients (e.g. obstetric history, gestational age, fundus height, breath rate in infants), consolidation of information of all antenatal care visits for a single pregnant woman in the

For peer review only

same page, among many other formal and contents improvements, including improved aesthetics. A systematic comparison of the new (intervention) and current (control) tools is provided in Table 1.

We aimed at creating "a system" (not just some tools) focusing on decision making by frontline health workers. The epidemiological and public health contexts in the three countries are similar, as confirmed by the similarities in the existing HIS between the three. The visual language and the recording forms where common to the three countries because clinical decisions are common to the three; although forms allowed for specific tests or treatments. The reporting component was adapted to each country set of indicators, although the visual language and reporting processes were harmonised.

During three or four days, HW were trained on HIS before the start of the trial. In the intervention arm they were trained on the PHISICC tools; and the control arm received a refresher training about the regular tools, during the same number of days.

Additionally, given that the regular tools already contained information on past vaccination history of children still to complete their vaccination schedule, we created a mechanism to retrieve data of children's vaccination status to transcribe into the new vaccination register book in the intervention arm ('system transition').

Tools were endorsed by MOH, printed in local printing companies and distributed to HW at the end of the training sessions. A digital spreadsheet was created to monitor consumption and order additional tools to cover health facility needs during the life of the trial.

Outcomes

There are five primary outcomes (

For peer review only - http://bmjopen.bmj.com/site/about/guidelines.xhtml

Table 2). Vaccination adherence is defined as the total number of vaccine doses given in the correct time interval to children in households in the health facilities catchment villages of those over the total number of vaccine doses that should have been given during the same period. Antenatal care visits uptake will also be considered depending on the expected number of pregnancies in the study areas. Both are used as proxies for health outcomes in terms of protection against disease [13] and prevention of pregnancy complications [14] and are assessed in a random sample of households in the health facilities catchment areas. Data concordance is defined as the level of agreement of HIS indicators between (i) records of health care events (re-counts), (ii) tallies (re-counts) and (iii) reports (aggregated data to higher levels of the system) [3]. Decision-making will be assessed considering the diagnostics scope in the sick child (i.e. number of different diagnoses per sick child consultation) and treatment appropriateness (i.e. number of prescribed treatments that are supported by a documented diagnosis). These outcomes will be assessed in a random sample of records and corresponding reports during the last four months of the study period. Health workers satisfaction will be assessed in all health workers in included health facilities using a standardised questionnaire [15,16,17]. While the intervention targets HF, some of the outcomes are measured at the level of HF, and some from patients clustered within HF catchment areas.

Secondary outcomes are classified under the following domains: data quality, data use, mortality, HW experience, client experience and resource consumption:

- Data quality, assessed in a sample of records
  - Completeness of recording and reporting in specific forms; i.e. prevalence of unduly missing data items; partograph used;
  - accuracy of recorded figures in comparison to real events (e.g. physical counting of commodities, such as number of 500mg Paracetamol tablets as recorded versus number of 500mg Paracetamol tablets as counted);
  - timeliness of reporting, as documented by time stamps in forms;
  - loss of data or data which does not reach the next upper administrative level.

- Data use, assessed in a sample of records
  - in terms of knowledge (e.g. vaccines due based on date of birth; weight for length assessments);
  - cases of different conditions properly treated in (e.g. diarrhoea cases given oral rehydration therapy according to national guidelines; pneumonia cases given appropriate antibiotic according to national guidelines;
  - public health decisions: availability of lost to follow up lists or plans for vaccination, tuberculosis and or HIV/AIDS treatment control;
  - occurrence of stock outs of essential drugs.
- Overall under-5s mortality and under-5s mortality excluding peri-natal mortality [18], in a sample of households in health facilities catchment areas.
- Health workers' 'human experience' and satisfaction (all health workers).
- District health information officers' 'human experience' (selected health care programme managers).
- Clients' 'human experience' and satisfaction, in a sample of households in health facilities catchment areas.
- Resources consumption (e.g. time use, costs)
  - intervention costs: tools, training, start-up;
  - time used for recording and reporting (e.g. time-motion study) [19];
  - cost-effectiveness per unit of additional improvement in outcomes of interest.

It is worthwhile to note that outcomes that do not relate to data quality and use will be assessed using additional data collection tools (e.g. survey questionnaires), which are the same for intervention and control health facilities. Hence, the effects of the intervention cannot be attributed to the changes in performance of the paper tools routinely used to record health care events in intervention and control health facilities, which are different by design.

For peer review only

In addition, we will consider 'explanatory outcomes' that will help to understand how the measured effects have taken place and why. We will look at the details of the interplay between the intervention, the system, the users and the context. Process indicators will be based on the documented activities that have taken place, from the conception of the intervention, up to its implementation, monitoring and evaluation. Process indicators may include: intervention set up and implementation, monitoring of the use of the intervention, special activities targeted at vulnerable populations, district reactions related to the intervention, handling of data coming from the new system, sustainability based on costs information and perceptions, alignment with national health policies and donor priorities. We will also explore health care services characteristics looking at generic indicators from health facilities, such human resources profiles and relations with the communities, population characteristics and system and context characteristics captured in early stages of the project, where data are available.

Sample size calculations

The required sample sizes for each primary outcome were determined using simulation to incorporate the clustering easily (Table 1) and to take the baseline and end-line surveys into account. Briefly, we simulated 1000 trials with variation between them caused by drawing different samples from the same distributions. We then used the regression models detailed in the data section to analyse each of the simulated trials and estimate the power as the proportion of trials which detected the effect of the intervention as significant. The simulation code was written in R (supplementary files 1 and 2).

For each country, we require the probability of α, a type I error (rejecting the null hypothesis when it is actually true) to be less 0.05 and the power to be at least 80%.

For vaccination adherence, using a sample size of 35 HF per arm, we would have 80% power in each country to detect as significant a difference between a proportion of due vaccines given from 75% in the control to 85% in the intervention arm, assuming one child per household, 30 households per HF

For peer review only

and a between-HF variation equivalent to a k of 0.25, where k is equal to the standard deviation divided by the mean. The value of k is unknown, but was chosen in line with general observations by Hayes and Bennet [20].

For data quality outcomes, with 35 HF per arm we would be able to detect as significant a difference from a ratio of 0.7 (reported : recorded) vaccinations in the control arm to 0.8 with the intervention with 80% power, assuming 100 recorded vaccinations per HF and a standard deviation of 0.25 in the ratios between HF.

In terms of diagnostic scope, we would be able to detect an increase in the proportion of child-visits with more than one diagnosis from 30% to 35% with 80% power with 35 HF per arm, 60 records per HF and assuming a k of 0.25.

We would be able to detect as significant an increase from 50% of treatments having a corresponding appropriate diagnosis to 60% with 80% power assuming 35 HF per arm, 1 treatment per child, 25 children per HF and variation between HF corresponding to k = 0.25.

For the outcome related to health workers' satisfaction, we would be able to detect as significant an increase from 50% of health workers satisfied to 75%, with 80% power assuming 35 HF, three health workers per HF and a variation between HF equivalent to k = 0.25. Since this variable is measured in the end-line survey only, we used the formula in Hayes and Bennet [20].

In summary, in each country we require 35 HF per arm, three HW per HF, 100 vaccination records per HF, 60 sick child records per health facility and 30 children per health facility catchment area.

Data collection and management

Data collection took place at baseline and will take place again at the end of the study. Data is collected from health facilities, from the households in the catchment areas of the included health facilities and also from district offices.

For data quality and data use outcomes, HF registers, tallies and reports will be scrutinised. For population based outcomes, we carry out household surveys at baseline and at end-line. We use

For peer review only

standard approaches for these types of surveys [21]. Households are visited, the research project is briefly introduced and consent requested. Ideally, mothers of alive children or women in child-bearing age were interviewed in order to obtain information on living children (i.e. vaccination history) and death events, respectively, using home-based records if available and accessible. Patients' satisfaction will be assessed using the PSQ-18 satisfaction questionnaire [22,23,24]. Essentially, the tool enables practitioners to investigate the extent to which their health care service meets the perceived needs of their client group and pinpoint areas for improvement [24]. The interview will be conducted with consenting patients as close to their care encounter as possible [25]. Data tools are translated into the official languages of the study countries and pilot tested for consistent meaning and relevance to the setting. Data collectors are also able to communicate in local languages. The Satisfaction of Employees in Health Care (SEHC) survey is a validated tool to assess staff satisfaction. It was first developed and validated in a low-income country (Ethiopia) [26] and later successfully validated in a high-income country (USA) [27].

We use a mix of paper and electronic data (ODK [28]) collection tools. Data collectors are trained to minimise error. Tools are piloted before implementing. ODK data is regularly stored and sent to secure servers, as soon as data collectors reach their office base. Data from paper tools is double entered and compared and sent to secure servers. Each data collection tool has its corresponding electronic database that is cleaned and submitted to the analyses. All data is anonymised at the point of data collection or as soon as possible in the data management process. Data is labelled with an arm code (e.g. 'A' or 'B') without any further information allowing to disclose which data items belong to the intervention or to the control arms, ensuring blinding during data analyses.

Quality will be assured through several mechanisms: piloting of data collection tools; thorough training of field workers; checking missing data related; double, independent data entry from papers into digital databases; early descriptive analyses to detect potential outliers; fieldworkers tracking and supervision.

For peer review only

Data analysis

The analysis will be carried out for each country separately, and based on intention-to-treat.

Baseline population and health facility characteristics (i.e. basic demographic characteristics of population and health workers, professional profile of health workers, health facility size and services) will be summarised. If large imbalances are observed at baseline, the variables can be used to adjust the effect estimate comparisons [29,30].

The analyses vary for the different primary outcomes due to the unit of measurement and levels of clustering, the type of variable, and whether measurements were taken at baseline and endpoint or endpoint only. We use regression models to allow us to estimate the effect of the outcome while flexibly accounting for these issues and allowing adjustment for potential confounders.

Logistic regression will be used for the binary variables: vaccine adherence is measured by determining whether each vaccine due was received, and treatment appropriateness by whether each treatment was correctly prescribed. Data concordance and diagnostic scope are count variables and may be analysed with Poisson regression, depending on their distribution. The regression model for HW satisfaction will depend on how it is distributed.

The outcomes have different levels of clustering (children or consultations, HW, HF). We will account for these levels of clustering by including random effects in the regression models.

Four of the primary outcomes are measured at baseline and end-line. The effect of the intervention will be estimated using an interaction term between arm and survey in the regression models: i.e. is the change in the outcome between baseline and follow-up in the intervention arm different to the change between baseline and follow-up in the control arm? The effect of HW satisfaction, measured only at end-line, will be estimated as the difference between the intervention and control arm.

All estimates for the effect of the intervention will be presented with 95% confidence intervals. The analyses will be carried out using R [31].

For peer review only

Measures to minimise bias

Statistical analyses will be carried out blindly, without knowledge of what health facilities or population in the catchment area belong to the intervention or control groups. Only when the analysis code is considered as definitive and fixed, will results be shared with the wider investigators team and the arms for health facilities and population will be disclosed.

Outcome measurement bias may take place where data from the HIS, which is the focus of the intervention, is used to measure outcomes. However, we will minimise this by assessing population based outcomes at household level.

Contamination (i.e. the intervention affects individuals or units assigned to the control arm) may happen via the exchanges between health workers from health facilities in both arms; for example: in monthly district data quality meetings, managerial meetings; or through inputs from supervisors who influence control health facilities with intervention tips encountered in health facilities of the intervention arms. One mechanism to address this issue is using a district-based cluster randomisation scheme. However, we consider that (i) contamination, despite increasing the awareness of health works in control health facilities, will hardly influence the decision making mechanisms that the HIS intervention focuses on; and (ii) randomisation at the level of district poses additional challenges that are not worth the marginal benefit of reducing a doubtful contamination [32].

The spill-over effect (i.e. benefits of the intervention extend beyond their direct recipients) [33] may take place in higher levels of the health systems; e.g. district data managers and programme managers may experience the benefits of better structured and more timely data produced in health facilities in the intervention arms. The trial will have no capacity to quantitatively account for spill overs at higher levels of the system, due to the limited number of higher level administrative areas that will be involved in the trial. However, through process indicators, we will consider potential benefits and harms of the intervention at higher levels of the system.

For peer review only

A challenge is the Hawthorne effect (i.e. observer effect). Both health workers in the intervention and in the control sites will have an awareness of being observed as data collection activities will be at the same level of intensity in the two arms. Therefore, there should be no differential effect.

Analyses will be based on the intention-to-treat. It is important to closely monitor if the intervention HFs consistently use the new HIS tools and approaches. The data collection team and the trial monitoring team will check if old forms are still being used in the intervention health facilities. However, we do not expect health facilities to migrate between intervention and control arms, or vice versa, due to feasibility issues. On the other hand, some household members in a given catchment area may decide to seek for health care in a health facility belonging to another trial arm. In these cases, households will be analysed as belonging to the original trial arm.

Ethics and dissemination

Ethics committees in Côte d'Ivoire, Mozambique and Nigeria approved the study in their respective countries. To date, some modifications to the protocol have taken place. In Côte d'Ivoire, we decided to select study areas close to the research institution base on logistics and practical reasons, instead of selecting an area in the north of the country, where poorer health indicators have been described. In Mozambique, the low density of HF per population implied extremely vast distances between HF and this, coupled with the rainy season, made the trial unfeasible in the originally selected Nampula province. After consultations, we decided to move the trial to the province of Inhambane and cancel the household survey. The allocation of HF to the intervention and control arms was completed using random number generation.

We plan to disseminate the findings of the trials as one of the few examples of studies assessing the effects of health information systems interventions using experimental study designs [34]. Most of the experimental studies on HIS are circumscribed to specific health care areas (e.g. tuberculosis, vaccination, cardiovascular disease) and very few have a system-wide approach (e.g. PHC) [34]. Experimental studies for health systems interventions are sometimes dismissed because of their

For peer review only

limited capacity to provide reliable explanations of complex health system issues [35]. While we acknowledge these limitations, there is also a need for more robust evidence on the effects of these types of health system interventions [36] and there are also good examples of experimental studies reporting findings that can make it to the policy arena [37]. When embarking on this research, we considered from the outset the type of evidence required to be disseminated and included into systematic reviews [38], guidance development [39] and eventually recommendations for policy and practice [40].

We acknowledge the challenges of carrying out research on health care provided to remote, rural communities (in this case in Sub-Saharan Africa). However, it is only in these remote areas where research about their specific problems and needs can take place. Challenges included long distances, poor conditions of roads, unreliable communications and limited food and accommodation services, all of them to be proactively handled to keep the quality of work and the morale of researchers and collaborators. We expect that the dissemination of findings in meetings, conferences and publications will contribute to a better understanding of what it takes to make research in challenging contexts.

The engagement and ownership of partners within this research has also been instrumental in order to plan and implement the CRCT. The intervention actually targets a governmental sub-system (the HIS) for which we required more than permission but also endorsement and active support. We achieved this level of collaboration by ensuring the participation of key stakeholders in key phases of the whole project, from inception till the implementation of the last phases, through frequent communication and workshops. The PHISICC programme includes targeted activities to keep decision-makers engaged and we are planning to share the findings through workshops as well as online and face-to-face events to disseminate the lessons learned from the trial and the whole research.

For peer review only

We also expect that the dissemination of our findings among partners and competitors will contribute to the current debates on the digitalisation of health information systems. WHO recommendations on the matter are weak since the underlying evidence to support these recommendations is inconsistent [41]. The principles and methodological approaches in PHISICC can be applied to the development of any technological solution, being on paper, digital or mixed.

Finally, we expect that the results of the trials, both quantitative and qualitative, will be able to inform policies on how to make HIS responsive to providers' decision-making needs, particularly in health services where the most vulnerable live.

For peer review only

Authors' contributions

XBC, AOI, AM, RBY and CAU prepared the proposal for the funding agency, conceived the study and produced the data collection tools. SG ensured the regulatory, ethical and trial monitoring components. AR developed the analytical approaches and made the sample size calculations. RBY, MS and SB adapted the protocol to the context of Côte d'Ivoire and managed the administrative and ethical approvals in the country; AOI, NE, ON, ANN and ABG likewise in Nigeria; AM, SML and GM, in Nampula province (Mozambique); JS and TM adapted the protocol and acquired ethical and administrative clearances for Inhambane province (Mozambique). DB is chair of the PHISICC Technical Advisory Group (TAG) and has coordinated multiple formal and informal inputs. LKK and DB have advised on the adequacy of the study protocol within the overall PHISICC proposal and TAG advices. All country teams participated in PHISICC workshops and ensured that the protocol was suitable to countries realities; developed data collection tools and training materials. They are responsible for the implementation of the trial in each country. XBC drafted the first version of the manuscript. All authors commented on several versions of the manuscript.

Acknowledgements

Members of the PHISICC Technical Advisory Group, chaired by David Brown: Blanche Anya (WHO AFRO), Abdallah Bchir (former Gavi), Marta Gacic-Dobo (WHO HQ), Richard Greffé (AIGA), Pamela Mitula (WHO AFRO), Sandy Oliver (UCL Institute) and Chris Wolf (BMGF).

Research collaborators: Momade Ali, Celso Belo, Bassirou Bonfoh, Lisa Diallo, John Ferreira, Bernard Guessanbi, Caitlin Jarrett, Inza Koné, Felix Malé, Kouadio M'bra, David O'Donnell, Damaris Rodríguez (Sonder Collective), Melanie Wendland and Meike Zuske (Swiss TPH).

Stakeholders from Côte d'Ivoire, Mozambique and Nigeria, participating in the consultation processes.

For peer review only

Data sharing statement

We report on a protocol of three Cluster Randomised Controlled Trials and therefore no data is available yet. On completion of the trials, access to data will be available from the national research institutions in Côte d'Ivoire, Mozambique and Nigeria, in publications and in the funding agency. Individual participants' data on vaccination and antenatal care outcomes as well as health workers and users perceptions will be anonymised and made available via an online data repository for any purpose and access will be granted following a review of requests by the Swiss TPH contract officer. Data, with DOIs, will be made available during the second semester of 2021.

Available documents include study protocols, analytical plan, informed consent forms and analytical code.

Ethical approvals in Côte d'Ivoire, Mozambique and Nigeria

- Comité National Ethique des Sciences de la Vie et de la Santé (CNESVS), reference: 024-19/MSHP/CNESVS-kp (Côte d'Ivoire)

- Comité Institucional de Bioética para Saúde da Universidade Lúrio, reference: 16.2/Julho/CBISUL/19 (Mozambique)

- Secretary, Government of Cross River State of Nigeria, Ministry of Health, Calabar Health Research Ethics Committee, reference: CRS/MH/HREC/018/Vol. V1/151 (Nigeria)

- Ethikkommission Nordwest- und Zentralschweiz (EKNZ), reference: 2018-01059 (Switzerland).

Funding

Funded by the Bill & Melinda Gates Foundation, grant number INV-010193 / OPP1135947.

For peer review only

Abbreviations

BMGF Bill & Melinda Gates Foundation

CRCT Cluster Randomised Controlled Trial

sd Standard deviation

HF Health Facility

HW Health worker

WHO World Health Organisation

Additional files

- Additional file 1: CONSORT statement checklist.

Competing interests

No, there are no competing interests for any author.

For peer review only

References

1 WHO. Monitoring results with health facility information systems: A technical consultation Glion-sur-Montreux, Switzerland, 11–12 June 2014. WHO 2014.

2 WHO. Health facility & community data toolkit. WHO 2014.

3 Bosch-Capblanch X, Ronveaux O, Doyle V, Remedios V, Bchir A. Accuracy and quality of immunization information systems in forty-one low income countries. Trop Med Int Health. 2009 Jan;14(1):2-10. doi: 10.1111/j.1365-3156.2008.02181.x. PMID: 19152556.

4 Loder E. Promises, promises. BMJ. 2017 Mar 23;356:j1446. doi: 10.1136/bmj.j1446. PMID: 28336513.

5 Wenger N, Méan M, Castioni J, Marques-Vidal P, Waeber G, Garnier A. Allocation of Internal Medicine Resident Time in a Swiss Hospital: A Time and Motion Study of Day and Evening Shifts. Ann Intern Med. 2017 Apr 18;166(8):579-586. doi: 10.7326/M16-2238. Epub 2017 Jan 31. PMID: 28135724.

6 Bosch-Capblanch X, Zuske M, Auer C, Amadasoun S. Effects of interventions to improve 'health information use systems' on the quality and use of information. National Institute for Health Research. PROSPERO - https://www.crd.york.ac.uk/prospero/display_record.php?RecordID=35218.

7 Bosch-Capblanch X, Zuske M, Auer C. Making information support systems relevant to decision-makers: an effectiveness systematic review and a framework synthesis. Abstracts of the 25th Cochrane Colloquium, Edinburgh, UK. Cochrane Database of Systematic Reviews 2018;(9 Suppl 1). https://doi.org/10.1002/14651858.CD201801.

8 Schulz KF, Altman DG, Moher D, for the CONSORT Group. CONSORT 2010 Statement: updated guidelines for reporting parallel group randomised trials.

9 Campbell MK, Piaggio G, Elbourne DR, Altman DG; for the CONSORT Group. Consort 2010 statement: extension to cluster randomised trials. BMJ. 2012;345:e5661.

10 R Core Team. R: A Language and Environment for Statistical Computing [Internet]. Vienna, Austria; 2020. Available from: https://www.R-project.org/

11 Bostoen K, Chalabi Z. Optimization of household survey sampling without sample frames. Int J Epidemiol. 2006 Jun;35(3):751-5. doi: 10.1093/ije/dyl019. Epub 2006 Feb 15. PMID: 16481364.

12 Johnson, Sandhu, Tyler, "The Next Step for Human-Centered Design in Global Public Health" Stanford Social Innovation Review, Oct. 22, 2019.

13 WHO. Global Vaccine Action Plan 2011-2020. WHO 2013.

For peer review only

14 Carroli G, Villar J, Piaggio G, Khan-Neelofur D, Gülmezoglu M, Mugford M, Lumbiganon P, Farnot U, Bersgjø P; WHO Antenatal Care Trial Research Group. WHO systematic review of randomised controlled trials of routine antenatal care. Lancet. 2001 May 19;357(9268):1565-70. doi: 10.1016/S0140-6736(00)04723-1. PMID: 11377643.

15 Alpern R, Canavan ME, Thompson JT, McNatt Z, Tatek D, Lindfield T, Bradley EH. Development of a brief instrument for assessing healthcare employee satisfaction in a low-income setting. PLoS One. 2013 Nov 5;8(11):e79053. doi: 10.1371/journal.pone.0079053. PMID: 24223878; PMCID: PMC3818514.

16 Doll WJ and Torkzadeh G. The measurement of end-user computing satisfaction. MIS Quarterly 1988;12(2):259–74; 19.

17 Ilias A, Yasoa MR, Razaka MZA and Rahmana RA. The study of end-user computing satisfaction (EUCS) on computerised accounting system (CAS) among Labuan F.T. Government sectors: a case study in the responsibility centres. Labuan e-Journal of Muamalat and Society 2007;1:1–13.

18 Arifeen SE, Hoque DM, Akter T, Rahman M, Hoque ME, Begum K, Chowdhury EK, Khan R, Blum LS, Ahmed S, Hossain MA, Siddik A, Begum N, Sadeq-ur Rahman Q, Haque TM, Billah SM, Islam M, Rumi RA, Law E, Al-Helal ZA, Baqui AH, Schellenberg J, Adam T, Moulton LH, Habicht JP, Scherpbier RW, Victora CG, Bryce J, Black RE. Effect of the Integrated Management of Childhood Illness strategy on childhood mortality and nutrition in a rural area in Bangladesh: a cluster randomised trial. Lancet. 2009 Aug 1;374(9687):393-403. doi: 10.1016/S0140-6736(09)60828-X. PubMed PMID: 19647607.

19 Tilahun H, Fekadu B, Abdisa H, Canavan M, Linnander E, Bradley EH, Berman P. Ethiopia's health extension workers use of work time on duty: time and motion study. Health Policy Plan. 2017 Apr 1;32(3):320-328. doi: 10.1093/heapol/czw129. PubMed PMID: 27658649.

20 Hayes RJ, Bennett S. Simple sample size calculation for cluster-randomized trials. Int J Epidemiol. 1999 Apr;28(2):319-26.

21 The DHS program. Demographic and Health surveys. https://dhsprogram.com/.

22 Thayaparan, A. a. (2013). The Patient Satisfaction Questionnaire Short Form (PSQ-18) as an adaptable, reliable, and validated tool for use in various settings. Med Educ Online, 18.

23 The Health Foundation. (2013, June). Measuring patient experience (no.18). Retrieved Nov 6, 2018, from www.health.org.uk: https://www.health.org.uk/publication/measuring-patient-experience.

For peer review only

24 Grogan, S. C. (2000). Validation of a questionnaire measuring patient satisfaction with general practitioner services. Quality in Health Care, 9, 210-215.

25 LaVela, S. L. (2014). Evaluation and measurement of patient experience. Patient Experience Journal, 1(1), Article 5.

26 Alpern, R. C. (2013, November). Development of a Brief Instrument for Assessing Healthcare Employee Satisfaction in a Low-Income Setting. PLOS ONE, 8(11), e79053.

27 Chang, E. C. (2017). Measuring job satisfaction among healthcare staff in the United States: a confirmatory factor analysis of the Satisfaction of Employees in health Care (SEHC) survey. International Journal for Quality in health Care, 29(2), 262-268.

28 Open Data Kit. https://opendatakit.org/. Accessed 5th February 2021.

29 Pocock S, et al. Subgroup analysis, covariate adjustment and baseline comparisons in clinical trial reporting: current practice and problems. Stat Med. 2002;21:2917-30.

30 The CONSORT Group. CONSORT Statement [Internet]; item 15.

31 R Core Team. R: A language and environment for statistical computing. R Foundation for Statistical Computing, Vienna, Austria. URL https://www.R-project.org/.

32 Torgerson David J. Contamination in trials: is cluster randomisation the answer? BMJ 2001; 322:355.

33 Benjamin-Chung J, Arnold BF, Berger D, Luby SP, Miguel E, Colford JM Jr, Hubbard AE. Spillover effects in epidemiology: parameters, study designs and methodological considerations. Int J Epidemiol. 2018 Feb 1;47(1):332-347. doi: 10.1093/ije/dyx201. PubMed PMID: 29106568; PubMed Central PMCID: PMC5837695.

34 Leon N, Balakrishna Y, Hohlfeld A, Odendaal WA, Schmidt BM, Zweigenthal V, Anstey Watkins J, Daniels K. Routine Health Information System (RHIS) improvements for strengthened health system management. Cochrane Database Syst Rev. 2020 Aug 13;8:CD012012. doi: 10.1002/14651858.CD012012.pub2. PMID: 32803893.

35 Allanson ER, Tunçalp Ö, Vogel JP, Khan DN, Oladapo OT, Long Q, Gülmezoglu AM. Implementation of effective practices in health facilities: a systematic review of cluster randomised trials. BMJ Glob Health. 2017 Jul 20;2(2):e000266. doi: 10.1136/bmjgh-2016-000266. PMID: 29081997; PMCID: PMC5656132.

For peer review only

36 Brown DW, Bosch-Capblanch X, Shimp L. Where Do We Go From Here? Defining an Agenda for Home-Based Records Research and Action Considering the 2018 WHO Guidelines. Glob Health Sci Pract. 2019 Mar 29;7(1):6-11. doi: 10.9745/GHSP-D-18-00431. PMID: 30877139; PMCID: PMC6538131.

37 Joos O, Silva R, Amouzou A, Moulton LH, Perin J, Bryce J, et al. Evaluation of a mhealth data quality intervention to improve documentation of pregnancy outcomes by health surveillance assistants in Malawi: a cluster randomized trial. PloS One 2016;11(1):e0145238. PMID: 26731401. DOI: https://doi.org/10.1371/journal.pone.0145238

38 Langlois EV, Ranson MK, Bärnighausen T, Bosch-Capblanch X, Daniels K, El-Jardali F, Ghaffar A, Grimshaw J, Haines A, Lavis JN, Lewin S, Meng Q, Oliver S, Pantoja T, Straus S, Shemilt I, Tovey D, Tugwell P, Waddington H, Wilson M, Yuan B, Røttingen JA. Advancing the field of health systems research synthesis. Syst Rev. 2015 Jul 10;4:90. doi: 10.1186/s13643-015-0080-9. PMID: 26159806; PMCID: PMC4498528.

39 Bosch-Capblanch X, Lavis JN, Lewin S, Atun R, Røttingen JA, Dröschel D, Beck L, Abalos E, El-Jardali F, Gilson L, Oliver S, Wyss K, Tugwell P, Kulier R, Pang T, Haines A. Guidance for evidence-informed policies about health systems: rationale for and challenges of guidance development. PLoS Med. 2012;9(3):e1001185. doi: 10.1371/journal.pmed.1001185. Epub 2012 Mar 6. PMID: 22412356; PMCID: PMC3295823.

40 WHO. WHO recommendations on home-based records for maternal, newborn and child health. WHO 2018.

41 WHO. Recommendations on digital interventions for health system strengthening. WHO guideline. WHO 2019.

For peer review only

Tables and Figures

Table 1. Comparison of new (intervention) and current (control) tools.

Characteristics	New (PHISICC intervention) tools	Old (control tools)
Development approach	Human Centred Design, co-creation with users	Centrally done, based on data and information experts
Visual language	Standardised across tools	No visual elements
Information structure	Following clinical processes	Tabular form, following reporting requirements
Decision aids	Icons representing mild, moderate and severe conditions	Not available
Register books layout	Landscape, DIN-A3	Depending on health care area; often much larger than DIN-A3
Tally sheets to aid counting events	For each health care area, to be filled as health care events take place	Only for vaccination, to be filled as vaccinations take place
Reporting	Integrated with tallying / counting	Requires revisiting register books at the end of the month

For peer review only

Table 2. Outcomes and parameters used to estimate the sample sizes.

	Outcome name	Subjects	Definition	Baseline estimate	Expected change	Comments
1	Vaccination adherence	Children under-1 in (sample of households in catchment areas)	Number of vaccines given in the previous calendar year over the number of vaccine due in the same period	75 given per 100 due	Increase of 10 per 100	Vaccines are clustered within children, and children within HFs
2	Data concordance	Recording tools in health facilities (samples of records)	Number of health care events (e.g. vaccinations, antenatal care consultations) recounted in the previous calendar year versus the number of health care events reported in the same time period	7 recounted for each 10 reported [3]	Increase of 1 recounted	A single estimate can be obtained in each HF or by time periods (no clustering)
3	Diagnostic scope	Records of sick child consultations (samples of records)	Number of diagnosis in each sick child consultation during the previous calendar year	30% with more than 1 diagnosis	35% with more than 1 diagnosis	Individual consultations are clustered within HF
4	Treatment appropriateness	Records of sick child consultations (samples of records)	Number of treatments correctly prescribed in each sick child consultation during the previous calendar year	Half appropriate over all consultation s	Increase to 60%	Individual consultations are clustered within HF (one treatment per child)
5	Health workforce satisfaction	Health workers (all health workers form include health facilities)	Degree (score) of satisfaction across all health facilities in each arm, with the intervention	50% satisfied	75% satisfied	Maybe two or three health workers can be approached in each health facility

Figure 1. PHISICC research programme structure, processes, deliverables and flow of evidence.

Footnote to Figure 1. WS: work stream. Timelines are approximate.

Figure 2. CONSORT diagram: trial flow chart.

Separate file.

For peer review only

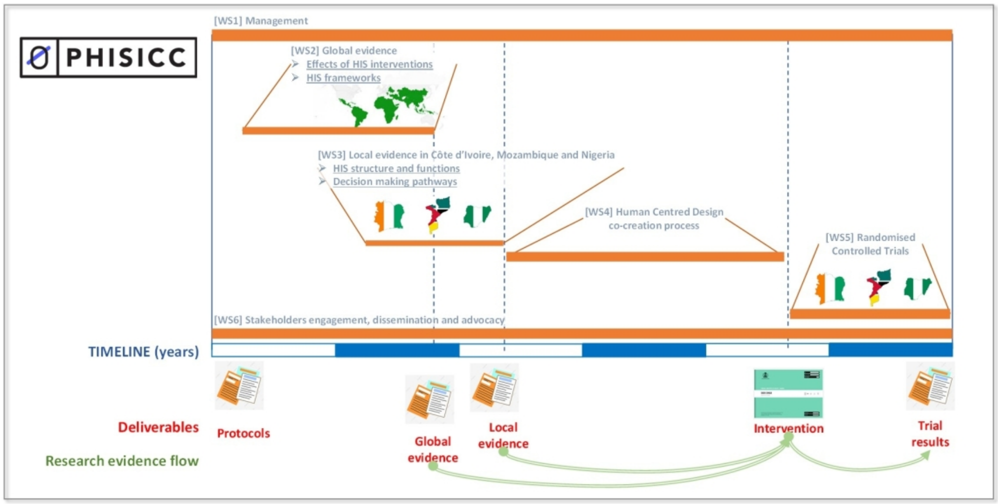

Figure 1. PHISICC research programme structure, processes, deliverables and flow of evidence.

312x157mm (150 x 150 DPI)

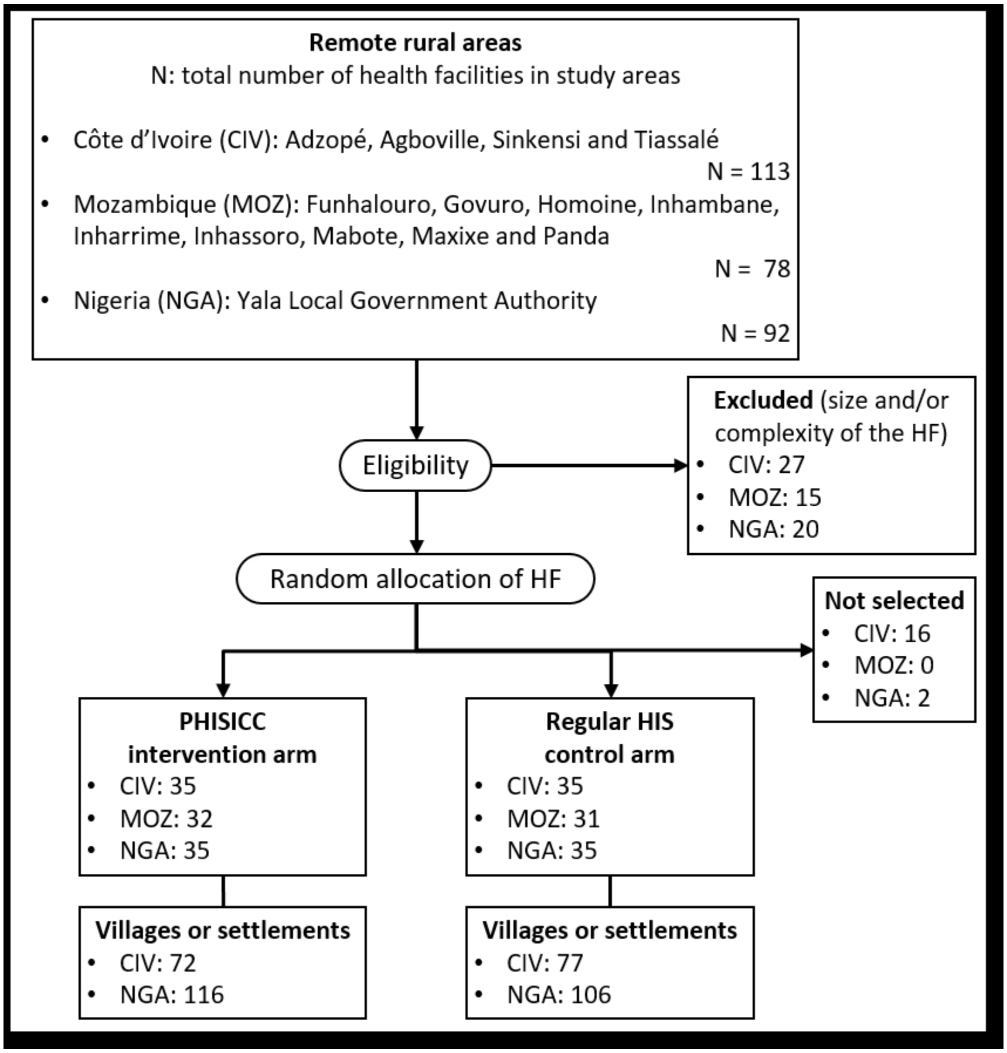

Figure 2. CONSORT diagram: trial flow chart.

144x150mm (150 x 150 DPI)

```
#
# clustersampleSize_proportions_baseline&endline.r
# get power of cluster randomised trial for binary outcomes (baseline and
endline surveys)
# 2 groups (control & intervention)
# clustered within HF


rm(list=ls())

# if the package lme4 is not already installed (needed for regression with
random effects)
# install.packages(lme4)
require(lme4)
#install.packages("reshape")
library(reshape)


# INPUTS
numGroups<-2
numHFPerGroup<-35
numTrialsToSimulate<-100
# numTrialsToSimulate: use 10 to test that the script runs, use 100 or 1000 for
precise estimate of power



# choose input set and remove #s to run

# inputs for 'treatments with appropriate diagnosis'
 pInterv<-0.60
 pControl<-0.50
 sdHFcluster<-0.55
# for k=0.1, 0.20; for k=0.25, 0.55
 numObsPerHF<-25

# inputs for vaccination adherence
# proportions in interventions and control groups
# pInterv<-0.8
# pControl<-0.75
# sdHFcluster<-2.63
# numObsPerHF<-30


# inputs for 'more than one diagnosis'
# pInterv<-0.35
# pControl<-0.30
# sdHFcluster<-0.39
# for k=0.1, 0.16; for k=0.25, 0.39
# numObsPerHF<-60


# NB getsd is a function at the bottom of the script to turn k into sdHFcluster
(sdHFcluster is on the logit scale)
```

For peer review only

```
# --- simulation ----

  # SET UP DATA STRUCTURE (intervention, HF)
  totNumHF <- numHFPerGroup*numGroups
  HFList<-seq(1:totNumHF)
  interv<- rep(c(0,1),each=(totNumHF/2) )
  intervEffect<-rep( c(0,(log(pInterv/(1-pInterv)) -
log(pControl/(1-pControl))) ), each=(totNumHF/2) )

  xtemp<-cbind(interv,HFList,intervEffect)

  # SET UP STORE FOR PVALUES AND PRECISION
  storeResults<-array(-9,dim=c(numTrialsToSimulate,3))
  colnames(storeResults)<-c("pvalue","coeff","stderr")


  # LOOP THROUGH THE SIMULATIONS

  for (i in 1:numTrialsToSimulate) {

    # simulate the HF cluster effects
      HFEffect<-rnorm(totNumHF,mean=0,sd=sdHFcluster)
      xtemp2a<-cbind(xtemp, HFEffect)
      xtemp2a<-data.frame(xtemp2a)

      # get expected proportions (pre and post)
      xtemp2a$expectedprelogodds<-log(pControl/(1-pControl)) + xtemp2a$HFEffect

      xtemp2a$expectedpostlogodds<-log(pControl/(1-pControl)) +
xtemp2a$intervEffect + xtemp2a$HFEffect

xtemp2a$expectedpre<-exp(xtemp2a$expectedprelogodds)/(1+exp(xtemp2a$expectedpre
logodds))

xtemp2a$expectedpost<-exp(xtemp2a$expectedpostlogodds)/(1+exp(xtemp2a$expectedp
ostlogodds))

      # expand by the number of observations per HF
      xtemp2b<-untable(xtemp2a, num=numObsPerHF)
      numObs<-dim(xtemp2b)[1]

    # simulate individual observations from cluster mean rates
      simObsPost<-rep(0,numObs)
      simObsPre<-rep(0,numObs)
      for (j in 1:numObs) {
         simObsPost[j]<-rbinom(n=1, size=1,prob=xtemp2b$expectedpost[j])
         simObsPre[j]<-rbinom(n=1, size=1,prob=xtemp2b$expectedpre[j])
      }
      # drop variables not needed further
      xtemp2b$expectedpostlogodds<-NULL; xtemp2b$expectedprelogodds<-NULL
```

For peer review only

```
    # stack pre and post observations
      # get post
      xtemp3<-cbind(xtemp2b,simObsPost)
      xtemp3<-data.frame(xtemp3)
      xtemp3$simObs<-xtemp3$simObsPost
      xtemp3$simObsPost<-NULL
      xtemp3$post<-1
      # get pre
      xtemp4<-cbind(xtemp2b,simObsPre)
      xtemp4<-data.frame(xtemp4)
      xtemp4$simObs<-xtemp4$simObsPre
      xtemp4$simObsPre<-NULL
      xtemp4$post<-0
      xtemp4$interv<-0
      xtemp5<-rbind(xtemp3,xtemp4)


     # carry out analysis for individual trial
      m <- glmer(simObs ~ as.factor(interv) +  post + (1 | HFList),
data<-xtemp5, family=binomial)

     # store result of individual trial in storeResults (p-value, coefficient
and std error)
          out1<-summary(m)$coefficients
          storeResults[i,2]<-out1[2,1]
          storeResults[i,3]<-out1[2,2]
          storeResults[i,1]<-out1[2,4]

   print(i)

  }  # End of loop

  # calculate power
  pvalue<-storeResults[,1]
  power<-length(pvalue[pvalue<0.05])/length(pvalue)

  cat("power ", power, "\n")



# -------- run to here -----







# ------
# getsd: function to estimate between-cluster variation from k (Hayes and
Bennet sd/mean) and input base proportion (base0p)
```

For peer review only

```
getsd<-function(base0p,k){
     sdcluster<-k*base0p
     clusterEffect<-rnorm(1000,mean=0,sd=sdcluster)
     expectedp<-base0p + clusterEffect
     expectedp[expectedp>1]<-0.9999
     expectedp[expectedp<0]<-0.0001
     logitexpectedp<-log((expectedp)/(1-expectedp))
     sdlog<-sd(logitexpectedp)
     cat("estimated sdlog ", sdlog, "\n")
}

# example
getsd(0.30,0.25)

getsd(0.50, 0.25)
```

For peer review only

```
#
# clusterSampleSize_concordance.r
# get power of cluster randomised trial
# ratios (outcome is continuous)
# fixed to 2 groups
# records and reports clustered within HF
#


# if the package lme4 is not already installed (needed for regression with
random effects)
# install.packages(lme4)
# install.packages(lmerTest)
require(lme4)
require(lmerTest)


# EXAMPLE INPUTS
numGroups<-2
numHFPerGroup<-35
numReportedPerHF<-100
# assuming equal numbers of vaccinations per HF
numTrialsToSimulate<-100
# 100 or 1000 needed for precision of the power estimate, use 10 for test runs

ratioControl<-0.7
ratioInterv<-0.8
sdHFcluster<-0.25*0.8
# sdHFcluster is on the log scale, calculated using k=0.25



# --- run simulation from here ----

  # SET UP DATA STRUCTURE (intervention, HF)
  totNumHF<-numGroups*numHFPerGroup
  HFList<-rep(seq(1:(numHFPerGroup*numGroups)),each=1)
  interv<-c( rep(c(0,1),each=(totNumHF/2)))
  intervEffect<-rep( c(0,(ratioInterv - ratioControl )), each=(totNumHF/2) )
  xtemp<-cbind(interv,HFList,intervEffect)

  # SET UP STORE FOR PVALUES AND PRECISION
  storeResults<-array(-9,dim=c(numTrialsToSimulate,3))
  colnames(storeResults)<-c("pvalue","coeff","stderr")


  # LOOP THROUGH THE SIMULATIONS

  for (i in 1:numTrialsToSimulate) {

    # simulate the HF cluster effects

      HFEffect<-rnorm(numHFPerGroup*numGroups,mean=0,sd=sdHFcluster)
      xtemp2<-cbind(xtemp, HFEffect)
```

For peer review only

```
      # get expected ratios (pre and post)
       expectedpreratio<-ratioControl + HFEffect
       expectedpostratio<-ratioControl + intervEffect + HFEffect
       expectedpreratio[expectedpreratio<0.0001]<-0.0001
       expectedpostratio[expectedpostratio<0.0001]<-0.0001

      # simulate individual observations as poisson rate of number reported per
 1 recorded
       simObsPost<-rep(0,length(expectedpostratio))
       simObsPre<-rep(0,length(expectedpreratio))
       for (j in 1:length(expectedpostratio)) {
           simObsPost[j]<-rpois(n=1,expectedpostratio[j]*numReportedPerHF)
           simObsPre[j]<-rpois(n=1,expectedpreratio[j]*numReportedPerHF)
       }


     # stack pre and post observations
       # post
       xtemp3<-cbind(xtemp2,simObsPost)
       xtemp3<-data.frame(xtemp3)
       xtemp3$simObs<-xtemp3$simObsPost
       xtemp3$simObsPost<-NULL
       xtemp3$post<-1
       # pre
       xtemp4<-cbind(xtemp2,simObsPre)
       xtemp4<-data.frame(xtemp4)
       xtemp4$simObs<-xtemp4$simObsPre
       xtemp4$simObsPre<-NULL
       xtemp4$post<-0
       xtemp4$interv<-0
       # stack pre and post
       xtemp5<-rbind(xtemp3,xtemp4)
         xtemp5$distanceToOne<-abs(1-(xtemp5$simObs/numReportedPerHF))


       # carry out analysis for individual trial
       m <- lmer(distanceToOne ~ as.factor(interv) +  post + (1|HFList),
 data=xtemp5)

       # store result of individual trial in storeResults (p-value, coefficient
 and std error)
        out1<-summary(m)$coefficients
        # estimate
        storeResults[i,2]<-out1[2,1]
        # se
        storeResults[i,3]<-out1[2,2]
        # p-value
        storeResults[i,1]<-out1[2,5]

      print(i)

   }  # End of loop
```

For peer review only

```
  # calculate power
  pvalue<-storeResults[,1]
  power<-length(pvalue[pvalue<0.05])/length(pvalue)

  cat("power ", power, "\n")


# -------------
```

For peer review only

Table 1: CONSORT 2010 checklist of information to include when reporting a cluster randomised trial

Section/Topic	Item No	Standard Checklist item	Extension for cluster designs	Page No *
**Title and abstract**				
	1a	Identification as a randomised trial in the title	Identification as a cluster randomised trial in the title	1
	1b	Structured summary of trial design, methods, results, and conclusions	See table 2	5
**Introduction**				
**Background and objectives**	2a	Scientific background and explanation of rationale	Rationale for using a cluster design	4
	2b	Specific objectives or hypotheses	Whether objectives pertain to the cluster level, the individual participant level or both	5
**Methods**				
**Trial design**	3a	Description of trial design (such as parallel, factorial) including allocation ratio	Definition of cluster and description of how the design features apply to the clusters	6
	3b	Important changes to methods after trial commencement (such as eligibility criteria), with reasons		Not applicable
**Participants**	4a	Eligibility criteria for participants	Eligibility criteria for clusters	6
	4b	Settings and locations where the data were collected		6
**Interventions**	5	The interventions for each group with sufficient details to allow replication, including how and when they were actually administered	Whether interventions pertain to the cluster level, the individual participant level or both	8
**Outcomes**	6a	Completely defined pre-specified primary and secondary outcome measures, including how and when they were assessed	Whether outcome measures pertain to the cluster level, the individual participant level or both	10 and Table 1
	6b	Any changes to trial outcomes after the trial commenced, with reasons		Not applicable
**Sample size**	7a	How sample size was determined	Method of calculation, number of clusters(s) (and whether equal or unequal cluster sizes are assumed), cluster size, a coefficient of intracluster correlation (ICC or *k*), and an indication of its uncertainty	12
	7b	When applicable, explanation of any interim analyses and stopping guidelines		Not yet applicable

Section/Topic	Item No	Standard Checklist item	Extension for cluster designs	Page No *
**Randomisation:**				
**Sequence generation**	8a	Method used to generate the random allocation sequence		7, 8
	8b	Type of randomisation; details of any restriction (such as blocking and block size)	Details of stratification or matching if used	Not applicblae
**Allocation concealment mechanism**	9	Mechanism used to implement the random allocation sequence (such as sequentially numbered containers), describing any steps taken to conceal the sequence until interventions were assigned	Specification that allocation was based on clusters rather than individuals and whether allocation concealment (if any) was at the cluster level, the individual participant level or both	7
**Implementation**	10	Who generated the random allocation sequence, who enrolled participants, and who assigned participants to interventions	Replace by 10a, 10b and 10c	7
	10a		Who generated the random allocation sequence, who enrolled clusters, and who assigned clusters to interventions	7
	10b		Mechanism by which individual participants were included in clusters for the purposes of the trial (such as complete enumeration, random sampling)	10
	10c		From whom consent was sought (representatives of the cluster, or individual cluster members, or both), and whether consent was sought before or after randomisation	8
**Blinding**	11a	If done, who was blinded after assignment to interventions (for example, participants, care providers, those assessing outcomes) and how		8
	11b	If relevant, description of the similarity of interventions		Not applicable
**Statistical methods**	12a	Statistical methods used to compare groups for primary and secondary outcomes	How clustering was taken into account	15
	12b	Methods for additional analyses, such as subgroup analyses and adjusted analyses		15

For peer review only

Section/Topic	Item No	Standard Checklist item	Extension for cluster designs	Page No *
**Results**				Not yet applicable (protocol manuscript)
**Discussion**				
**Limitations**	20	Trial limitations, addressing sources of potential bias, imprecision, and, if relevant, multiplicity of analyses		18
**Generalisability**	21	Generalisability (external validity, applicability) of the trial findings	Generalisability to clusters and/or individual participants (as relevant)	18,19
**Interpretation**	22	Interpretation consistent with results, balancing benefits and harms, and considering other relevant evidence		Not yet applicable
**Other information**				
**Registration**	23	Registration number and name of trial registry		1
**Protocol**	24	Where the full trial protocol can be accessed, if available		1
**Funding**	25	Sources of funding and other support (such as supply of drugs), role of funders		20

** Page numbers: as seen in the document "draft_Proof_hi.pdf" (which has 34 pages)*

For peer review only

CONSORT 2010 checklist of information to include when reporting a cluster randomised trial

Section/Topic	Item No	Standard Checklist item	Extension for cluster designs	Page No *
**Title and abstract**				
	1a	Identification as a randomised trial in the title	Identification as a cluster randomised trial in the title	1
	1b	Structured summary of trial design, methods, results, and conclusions	See table 2	5
**Introduction**				
**Background and objectives**	2a	Scientific background and explanation of rationale	Rationale for using a cluster design	4
	2b	Specific objectives or hypotheses	Whether objectives pertain to the cluster level, the individual participant level or both	5
**Methods**				
**Trial design**	3a	Description of trial design (such as parallel, factorial) including allocation ratio	Definition of cluster and description of how the design features apply to the clusters	6
	3b	Important changes to methods after trial commencement (such as eligibility criteria), with reasons		Not applicable
**Participants**	4a	Eligibility criteria for participants	Eligibility criteria for clusters	6
	4b	Settings and locations where the data were collected		6
**Interventions**	5	The interventions for each group with sufficient details to allow replication, including how and when they were actually administered	Whether interventions pertain to the cluster level, the individual participant level or both	8
**Outcomes**	6a	Completely defined pre-specified primary and secondary outcome measures, including how and when they were assessed	Whether outcome measures pertain to the cluster level, the individual participant level or both	10 and Table 1
	6b	Any changes to trial outcomes after the trial commenced, with reasons		Not applicable
**Sample size**	7a	How sample size was determined	Method of calculation, number of clusters(s) (and whether equal or unequal cluster sizes are assumed), cluster size, a coefficient of intracluster correlation (ICC or *k*), and an indication of its uncertainty	12
	7b	When applicable, explanation of any interim analyses and stopping guidelines		Not yet applicable

For peer review only - http://bmjopen.bmj.com/site/about/guidelines.xhtml

Section/Topic	Item No	Standard Checklist item	Extension for cluster designs	Page No *
**Randomisation:**				
**Sequence generation**	8a	Method used to generate the random allocation sequence		7, 8
	8b	Type of randomisation; details of any restriction (such as blocking and block size)	Details of stratification or matching if used	Not applicable
**Allocation concealment mechanism**	9	Mechanism used to implement the random allocation sequence (such as sequentially numbered containers), describing any steps taken to conceal the sequence until interventions were assigned	Specification that allocation was based on clusters rather than individuals and whether allocation concealment (if any) was at the cluster level, the individual participant level or both	7
**Implementation**	10	Who generated the random allocation sequence, who enrolled participants, and who assigned participants to interventions	Replace by 10a, 10b and 10c	7
	10a		Who generated the random allocation sequence, who enrolled clusters, and who assigned clusters to interventions	7
	10b		Mechanism by which individual participants were included in clusters for the purposes of the trial (such as complete enumeration, random sampling)	10
	10c		From whom consent was sought (representatives of the cluster, or individual cluster members, or both), and whether consent was sought before or after randomisation	8
**Blinding**	11a	If done, who was blinded after assignment to interventions (for example, participants, care providers, those assessing outcomes) and how		8
	11b	If relevant, description of the similarity of interventions		Not applicable
**Statistical methods**	12a	Statistical methods used to compare groups for primary and secondary outcomes	How clustering was taken into account	15
	12b	Methods for additional analyses, such as subgroup analyses and adjusted analyses		15

For peer review only

Section/Topic	Item No	Standard Checklist item	Extension for cluster designs	Page No *
**Results**				Not yet applicable (protocol manuscript)
**Discussion**				
**Limitations**	20	Trial limitations, addressing sources of potential bias, imprecision, and, if relevant, multiplicity of analyses		18
**Generalisability**	21	Generalisability (external validity, applicability) of the trial findings	Generalisability to clusters and/or individual participants (as relevant)	18,19
**Interpretation**	22	Interpretation consistent with results, balancing benefits and harms, and considering other relevant evidence		Not yet applicable
**Other information**				
**Registration**	23	Registration number and name of trial registry		1
**Protocol**	24	Where the full trial protocol can be accessed, if available		1
**Funding**	25	Sources of funding and other support (such as supply of drugs), role of funders		20

For peer review only
